# Experimental realization of the topologically nontrivial phase in monolayer Si_2_Te_2_

**DOI:** 10.1038/s41467-026-77669-9

**Published:** 2026-09-11

**Authors:** Xiaochun Huang, Lingxiao Zhao, Rui Xiong, Wenbin Li, Bao-Tian Wang, Baisheng Sa, Matthias Bode

**Affiliations:** 1 Physikalisches Institut, Experimentelle Physik 2, Universität Würzburg, Würzburg, Germany; 2 Quantum Science Center of Guangdong-Hong Kong-Macao Greater Bay Area (Guangdong), Shenzhen, China; 3 Multiscale Computational Materials Facility, Key Laboratory of Eco-materials Advanced Technology, College of Materials Science and Engineering, Fuzhou University, Fuzhou, China; 4 The Institute for Solid State Physics, The University of Tokyo, Chiba, Japan; 5 Institute of High Energy Physics, Chinese Academy of Sciences, Beijing, China; 6 Spallation Neutron Source Science Center, Dongguan, China; 7 Wilhelm Conrad Röntgen-Center for Complex Material Systems (RCCM), Universität Würzburg, Würzburg, Germany; 8 Present Address: Department of Chemistry, National University of Singapore, Singapore, Singapore

**Keywords:** Topological insulators, Surfaces, interfaces and thin films, Electronic properties and materials

## Abstract

Quantum spin Hall insulators, with dissipationless edge channels protected by time-reversal symmetry, are promising for low-power and quantum devices. However, experimentally realized examples with sizable nontrivial gaps remain scarce. Monolayer Si_2_Te_2_ has been theoretically predicted to host a room-temperature quantum spin Hall phase, but the absence of a bulk analog has hindered its experimental realization. Here we show that HfTe_2_ provides an ideal van der Waals template for the epitaxial growth of strain-free monolayer Si_2_Te_2_ while preserving its nontrivial topological phase. Scanning tunneling microscopy and spectroscopy reveal an intact (1×1) lattice and a bulk band gap of  ~ 300 meV, consistent with first-principles calculations. Moreover, pronounced edge states extending ~ 2.0 nm from the island boundary are observed in monolayer Si_2_Te_2_, exhibiting characteristics expected for topological edge modes. Our results establish monolayer Si_2_Te_2_ as a large-gap two-dimensional topological-insulator platform for topological phenomena and device concepts at elevated temperatures.

## Abstract (web summary)

Band topology can be highly sensitive to lattice strain. Here, the authors experimentally realize a strain-free monolayer Si_2_Te_2_ on a HfTe_2_ substrate with a large bandgap and pronounced in-gap edge-state features consistent with topological edge states.

## Introduction

Quantum spin Hall (QSH) insulators, also known as two-dimensional (2D) topological insulators (TIs), host one-dimensional (1D) helical edge states with Dirac-like linear dispersion dictated by bulk band topology^1–4^. Protected by time-reversal symmetry (TRS), these edge states are immune to backscattering, offering potential applications in low-energy consumption devices^3–5^. While 2D TI phases have been experimentally demonstrated in HgTe/CdTe and InAs/GaSb quantum wells^6,7^, their small bulk band gaps—typically on the order of meV—limit both further experimental studies and practical applications. In recent years, extensive efforts have been devoted to identify new 2D TIs with sizable band gaps, tapping into the vast potential of emerging 2D materials^8–10^.

A common strategy is to realize 2D TI phases by reducing three-dimensional (3D) materials to the monolayer (ML) limit, as demonstrated in WTe_2_^11–13^, ZrTe_5_^14,15^, and Bi_4_Br_4_ monolayers^16,17^, though with limited success. Alternatively, a variety of 2D TIs with artificial lattice structures have been theoretically proposed, enabled by specific lattice symmetries and strong spin-orbit coupling (SOC), thereby broadening the range of potential candidates^18^. However, among these, only a few, primarily graphene-like elementary 2D materials, such as germanene^19^, stanene^20,21^, and bismuthene^22–24^, have been successfully synthesized and confirmed as topologically nontrivial. Furthermore, substantial substrate interactions are often necessary to stabilize their artificial lattices^25,26^.

Monolayer Si_2_Te_2_ (ML-Si_2_Te_2_) possesses an artificial lattice structure and is predicted to exhibit a room-temperature QSH phase^27,28^. This 2D material features a hexagonal ($P\overline{3}$ m1) symmetry with a characteristic Te–Si–Si–Te stacking sequence, suggesting that only weak van der Waals (vdW) interactions with the substrate may be required^28^. Density functional theory (DFT) calculations indicate that free-standing ML-Si_2_Te_2_ has a topologically nontrivial band gap of 220 meV, while its band topology is highly sensitive to lattice strain^28,29^. Since ML-Si_2_Te_2_ has no 3D counterpart, it cannot be obtained via mechanical exfoliation from a bulk material. Recently, we successfully achieved the epitaxial growth of ML-Si_2_Te_2_ on an Sb_2_Te_3_ substrate^29,30^. Nevertheless, the large lattice mismatch between free-standing ML-Si_2_Te_2_ and Sb_2_Te_3_ induces significant interfacial strain, driving the system into a trivial semiconducting phase^29,30^.

In this work, by combining DFT, molecular beam epitaxy (MBE), and scanning tunneling microscopy/spectroscopy (STM/STS), we experimentally realize strain-free (1 × 1) ML-Si_2_Te_2_ on a HfTe_2_ substrate. The sizable bulk band gap predicted for ML-Si_2_Te_2_ by DFT is corroborated by differential tunneling conductivity (d*I*/d*V*) measurements. Most importantly, we observe pronounced in-gap edge-state features with boundary-localized character and a characteristic spatial extent of ≈ 1.5 − 2.0 nm, consistent with the theoretically predicted topological edge states in ML-Si_2_Te_2_. These results establish ML-Si_2_Te_2_ as a promising large-gap 2D TI candidate and provide a platform for further investigation of QSH physics at elevated temperatures.

## Results and Discussion

### Substrate screening

**Fig. 1:**
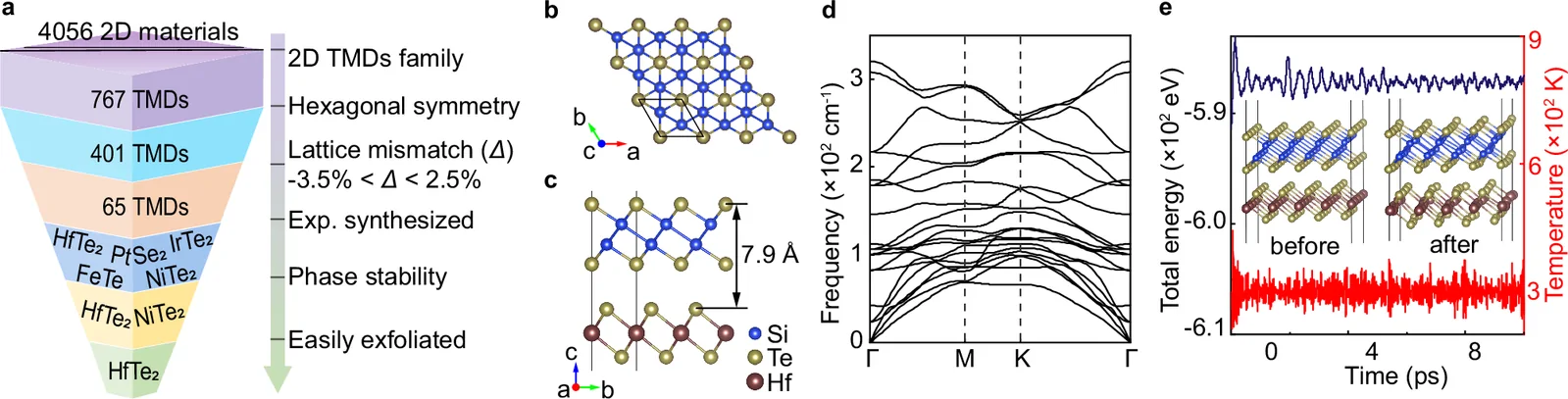
Monolayer Si_2_Te_2_ (ML-Si_2_Te_2_) on HfTe_2_. **a** Screening process of two-dimensional (2D) transition metal chalcogenides (TMDs) as substrates for the epitaxial growth of ML-Si_2_Te_2_. **b**, **c** Top and side views, respectively, of the structural model of ML-Si_2_Te_2_ on HfTe_2_, with the unit cell outlined by a diamond (top view) and vertical lines (side view). Colored arrows indicate the crystallographic *a*-, *b*-, and *c*-axis directions. The double-headed arrow marks the interlayer spacing between ML-Si_2_Te_2_ and HfTe_2_. **d** Calculated phonon dispersion curves of the ML-Si_2_Te_2_/HfTe_2_ heterostructure. **e** Main panel: The dark blue and red curves show the evolution of the total energy and temperature, respectively, during ab initio molecular dynamics (AIMD) simulations of the ML-Si_2_Te_2_/ML-HfTe_2_ heterostructure using a 4 × 4 supercell. Inset: Schematic views of the heterostructure before and after 10 ps of simulation at 300 K. Vertical lines indicate the supercell boundaries.

To identify a suitable substrate, we screened candidates from the Computational 2D Materials Database (C2DB)^31,32^, which contains 4056 2D materials, see Fig. 1a. Transition metal chalcogenides (TMDs) were chosen as the initial screening criterion due to their structural diversity. Among the 767 TMDs, 401 exhibit trigonal (or hexagonal) symmetry, matching that of ML-Si_2_Te_2_. Our previous work shows that maintaining the topological phase of ML-Si_2_Te_2_ requires an in-plane strain within −3% to 2% relative to the free-standing case (*a*_0_ = *b*_0_ = 389 pm)^29^. To account for vdW interactions, which can enhance lattice mismatch tolerance, we adopted a slightly broader criterion of −3.5 to 2.5%, defined as (*a*_0_ − *a*_TMD_)/*a*_0_, yielding 65 candidates.

Filtering for materials that have been successfully synthesized narrows the selection down to five candidates: IrTe_2_, NiTe_2_, FeTe, PtSe_2_, and HfTe_2_. IrTe_2_ transitions to a triclinic phase below ≈ 270 K^33,34^, and hexagonal FeTe is stable only above 983 K^35^, making both unsuitable for low-temperature STM/STS measurements. PtSe_2_ was excluded to prevent potential Se–Te substitution or alloying during Si_2_Te_2_ growth. To ensure an atomically flat surface via cleaving in ultrahigh vacuum (UHV), we evaluated the exfoliation energies of NiTe_2_ and HfTe_2_. As shown in Supplementary Fig. 1, HfTe_2_ exhibits an apparently lower exfoliation energy than NiTe_2_, indicating easier cleavage. Based on these considerations, we selected HfTe_2_ as the substrate.

### DFT validation of ML-Si_2_Te_2_/HfTe_2_ heterostructures

The structural stability of ML-Si_2_Te_2_ on HfTe_2_ was investigated via DFT calculations employing the Vienna ab initio simulation package (VASP)^36^ (Methods). Figure 1b and 1c illustrate the ML-Si_2_Te_2_/ML-HfTe_2_ heterostructure, which serves as the model configuration for our calculations. After full structural relaxation, the in-plane lattice constants of ML-Si_2_Te_2_ remain at *a*_DFT_ = *b*_DFT_ = 389 pm, identical to those of the free-standing case, with an interlayer spacing of 790 pm. The calculated binding energy of  −0.296 eV per unit cell indicates a typical vdW interaction (see Supplementary Information).

Figure 1d presents the phonon dispersion curves, where the absence of imaginary frequencies confirms the dynamic stability of the heterojunction. Further ab initio molecular dynamics (AIMD) simulations^37^ demonstrated the thermal stability of the ML-Si_2_Te_2_/ML-HfTe_2_ heterostructure. As shown in Fig. 1e and Supplementary Fig. 2, the total energy fluctuates only within a small range, while the lattice structure remains stable during AIMD simulations at 300 K for up to 100 ps. We note that AIMD simulations are inherently limited to relatively short timescales. Additional discussions on the structural and electronic stability of ML-Si_2_Te_2_ on HfTe_2_, together with supporting experimental observations, are provided below.

Next, electronic-structure calculations were performed using the Heyd–Scuseria–Ernzerhof (HSE06) hybrid functional to investigate the band topology of ML-Si_2_Te_2_ on HfTe_2_^38^. Because the band ordering and topological properties of heavy-element telluride systems can be sensitive to the choice of the exchange-correlation functional, we first compared the electronic structures of free-standing ML-Si_2_Te_2_ obtained from HSE and GW calculations (see Supplementary Information). Both approaches yield the same SOC-induced band inversion at the *Γ* point, indicating that the nontrivial band topology is robust with respect to the computational approach. The free-standing system also serves as a useful reference for assessing the influence of the HfTe_2_ substrate.

**Fig. 2:**
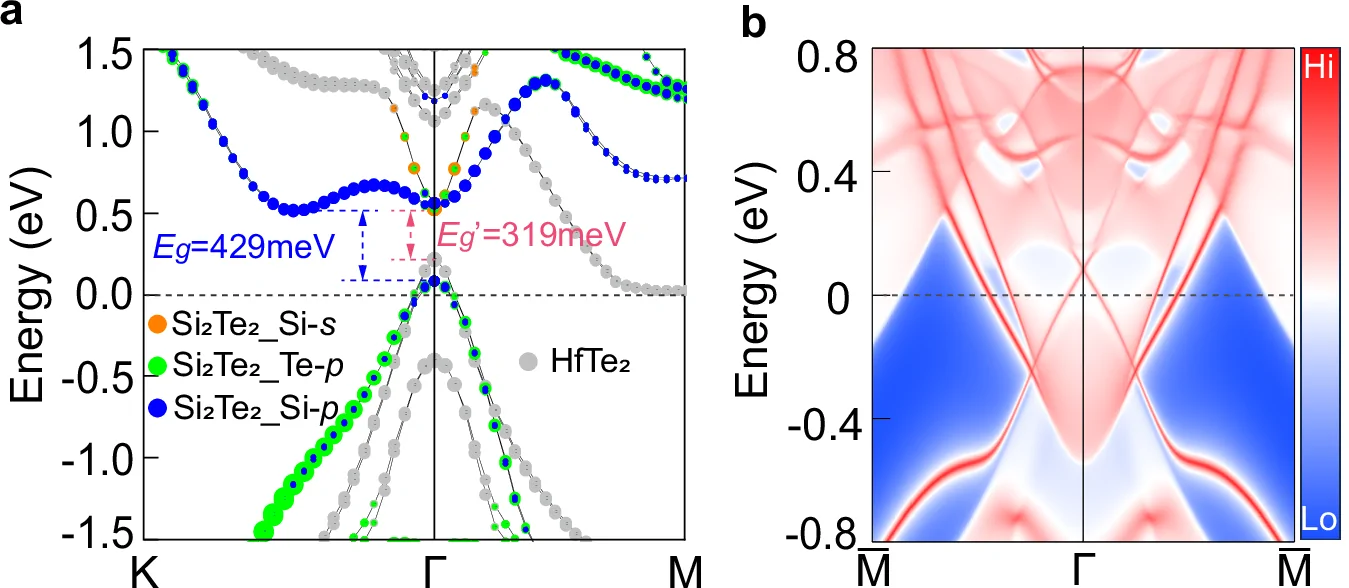
Calculated band structures of a ML-Si_2_Te_2_/ML-HfTe_2_ heterostructure with spin-orbit coupling (SOC). **a** Projected band structure. Solid lines represent the calculated band dispersion, while the symbol size indicates the orbital contribution. Orange, green, and blue symbols denote Si-*s*, Te-*p*, and Si-*p* orbital contributions from ML-Si_2_Te_2_, respectively, while gray symbols indicate contributions from HfTe_2_. Blue and pink double-headed arrows mark the corresponding band gaps, *E*_*g*_ and ${E}_{g}^{{\prime} }$, respectively. **b** Spectral density of the edge states. Gray dashed lines in (**a**) and (**b**) indicate the Fermi levels. The color scale represents the spectral density.

Figure 2a and Supplementary Fig. 3 present the calculated decomposed band structures of the ML-Si_2_Te_2_/ML-HfTe_2_ heterostructure. As shown, the projected band dispersion of ML-Si_2_Te_2_ in the heterojunction closely resembles that of the free-standing case and preserves the SOC-induced band inversion at the *Γ* point, indicating that the topologically nontrivial phase remains intact. Notably, substrate interactions shift the conduction band minimum (CBM) of ML-Si_2_Te_2_ upward, enhancing its nontrivial band gap to 429 meV. Given that the valence band maximum (VBM) of ML-HfTe_2_ lies 110 meV above that of ML-Si_2_Te_2_, the experimentally observed band gap at the *Γ* point is expected to be ≈ 319 meV. Besides, the Fermi level (*E*_F_) is shifted downwards below the VBM of ML-Si_2_Te_2_ because of the higher work function of ML-HfTe_2_ relative to ML-Si_2_Te_2_ (see Supplementary Information for details).

Figure 2b presents the calculated local density of states (LDOS) for the edge states, revealing a linear dispersion at *Γ*. Comparison with the free-standing case (Supplementary Fig. 5) confirms their Dirac-like dispersion. The nontrivial topology is further verified by calculating the *Z*_2_ invariant via the evolution of Wannier charge centers (WCCs)^39,40^ (see Methods and Supplementary Information for computational details). As shown in Supplementary Fig. 5, the WCC evolution curves for both the heterostructure and free-standing ML-Si_2_Te_2_ cross an arbitrary horizontal reference line an odd number of times, confirming *Z*_2_ = 1 in both cases. To validate the Wannier representation used in WCC calculations, the Wannier-interpolated band structures were compared with the directly calculated HSE+SOC band structures. As shown in Supplementary Fig. 6, good agreement is obtained within the energy window between ±3.0 eV, supporting the reliability of the subsequent WCC and topological-invariant calculations. The above DFT results indicate that strain-free ML-Si_2_Te_2_ with a QSH phase can be realized on a HfTe_2_ substrate.

### Epitaxial growth of ML-Si_2_Te_2_ on HfTe_2_

High-quality HfTe_2_ single crystals, with lateral dimensions of several millimeters, were synthesized via chemical vapor transport, using I_2_ as the transport agent, in a two-zone furnace (see Methods and Supplementary Fig. 7 for details). Epitaxial growth and electronic characterization of ML-Si_2_Te_2_ on HfTe_2_ were performed in a two-chamber UHV-MBE system with a base pressure *p* < 3 × 10^−11 ^Torr and an integrated home-built low-temperature STM. In-situ STM/STS measurements were conducted at 5.8 K with W-tips (Methods). Atomically smooth HfTe_2_ surfaces were obtained by cleaving the crystals under UHV conditions (see Supplementary Fig. 8). Monolayer Si_2_Te_2_ was fabricated by co-evaporating high-purity Si (99.999%) and Te (99.9999%) onto the HfTe_2_ substrate at 200 ^∘^C, followed by post-annealing at elevated temperature.

Annealing studies detailed in Supplementary Fig. 9 show that high-quality ML-Si_2_Te_2_ is achieved at 385 ^∘^C, although a significant reduction in coverage is observed above 320 ^∘^C—well below the crystallization threshold of ML-Si_2_Te_2_. To counteract the decomposition of SiTe_*x*_ and maintain sufficient precursor material on the substrate, we continuously supplied both Si and Te fluxes exceeding 20 times the nominal co-evaporation rate during annealing, thereby enabling the successful growth of ML-Si_2_Te_2_ (see Methods for details). To increase the coverage of ML-Si_2_Te_2_ islands, repeated growth/annealing cycles were performed on the same substrate. The persistence of ML-Si_2_Te_2_ islands under these repeated high-temperature processing steps suggests good structural stability of the heterostructure. In addition, after growth, many samples were kept in the UHV chamber at room temperature for more than 24 hours before being transferred to the low-temperature STM system, while maintaining stable morphology and reproducible spectroscopic characteristics. These experimental observations support the structural stability of ML-Si_2_Te_2_ on HfTe_2_ at room-temperature under vacuum conditions.

**Fig. 3:**
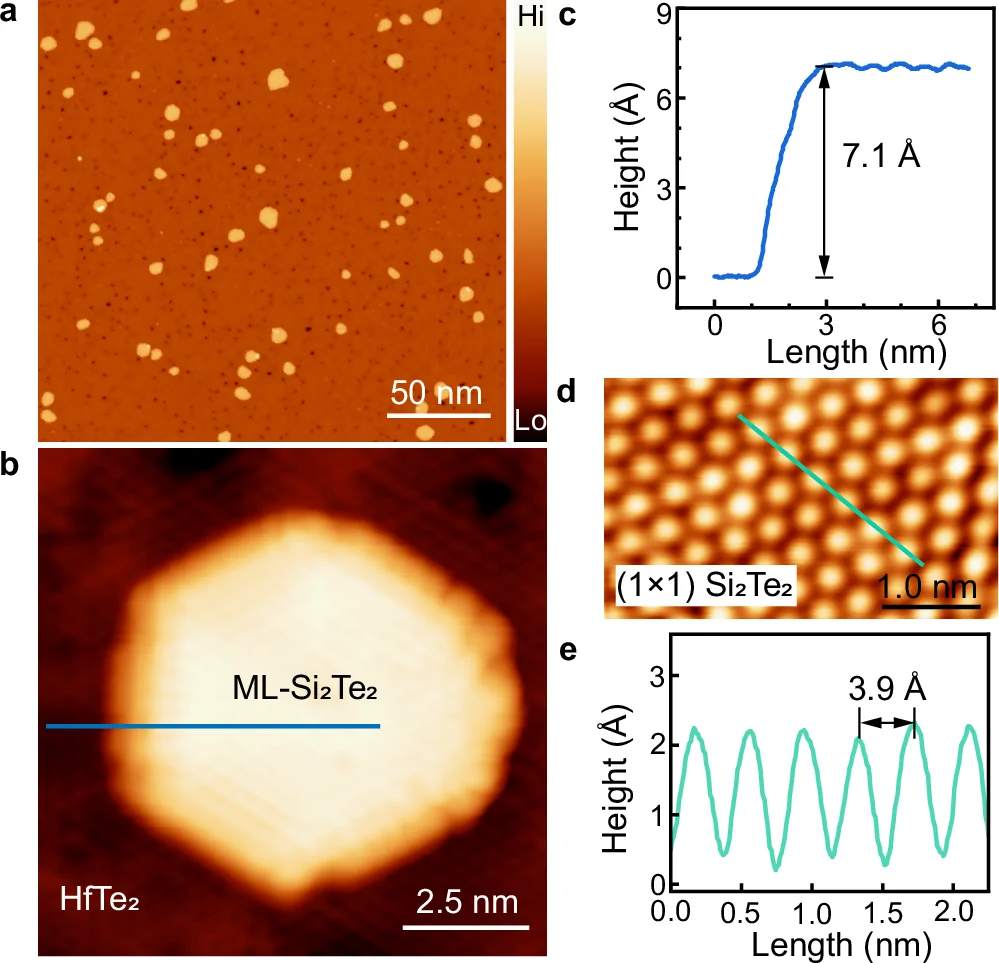
Morphology of ML-Si_2_Te_2_ grown on HfTe_2_. **a** Large-scale STM image (*V* = 1.0 V, *I* = 10 pA) of ML-Si_2_Te_2_ grown on a HfTe_2_ substrate. The color scale represents apparent height. **b** High-resolution STM image (*V* = 0.5 V, *I* = 200 pA) showing the surface lattice structure of both ML-Si_2_Te_2_ and the HfTe_2_ substrate simultaneously. **c** Height profile taken along the blue line in (**b**). The perpendicular double-headed arrows mark the step height. **d** Atomic-resolution STM image (*V* = 0.5 V, *I* = 100 pA) of ML-Si_2_Te_2_. The observed lattice corresponds to the (1 × 1) ML-Si_2_Te_2_ structure. **e** Line profile, taken along the green line in (**d**). The horizontal double-headed arrows mark the measured in-plane lattice constant.

Figure 3a shows a large-scale STM image of the as-grown sample, providing an overview of its morphology. A close-up STM image of an island (Fig. 3b) reveals the hexagonal surface structure of both Si_2_Te_2_ and the underlying HfTe_2_ substrate simultaneously. The step height of 710 pm, extracted from the line profile in Fig. 3c, is in reasonable agreement with the calculated interlayer spacing^29,41^. Atomic-resolution STM imaging (Fig. 3d) yields in-plane lattice constants of ML-Si_2_Te_2_ (${a}_{\exp }={b}_{\exp }=390$ pm), in good agreement with theoretical values (*a*_DFT_ = *b*_DFT_ = 389 pm). Systematic measurements across multiple samples consistently reveal a well-defined epitaxial relationship, where ML-Si_2_Te_2_ islands align with the HfTe_2_ substrate in a hollow-site configuration, consistent with DFT calculations (see Supplementary Figs. 10 and 11). These results show clear evidence for the experimental realization of strain-free (1 × 1) ML-Si_2_Te_2_.

### Electronic structure of ML-Si_2_Te_2_ on HfTe_2_

**Fig. 4:**
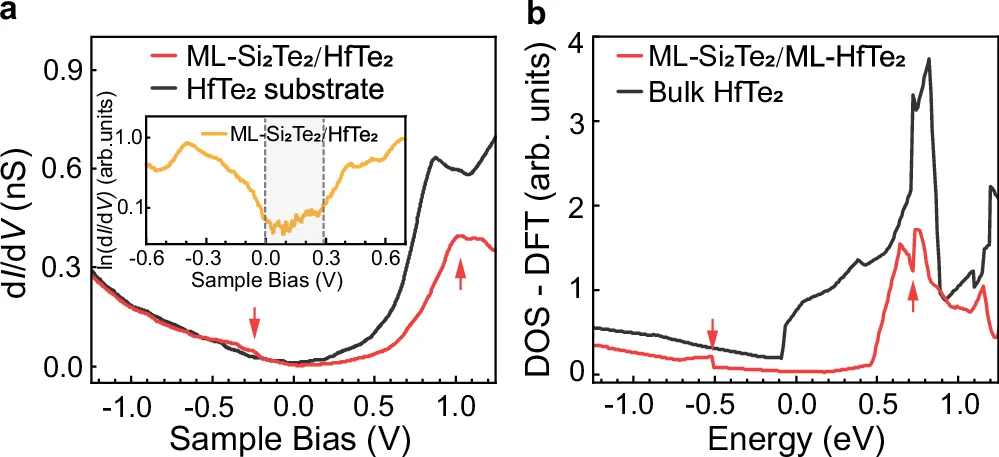
Local density of states of ML-Si_2_Te_2_ on HfTe_2_. **a** Main panel: Local d*I*/d*V* spectra (*V*_stab_ = − 1.5 V, *I*_stab_ = 200 pA, and *V*_mod_ = 10 mV) measured on ML-Si_2_Te_2_ and the HfTe_2_ substrate. The red arrows indicate two peak features in the spectrum measured on ML-Si_2_Te_2_. Inset: Logarithmic plot of a high-resolution d*I*/d*V* spectrum (*V*_stab_ = − 1.0 V, *I*_stab_ = 100 pA, and *V*_mod_ = 10 mV) acquired on ML-Si_2_Te_2_. The gray dashed lines mark the measured conduction and valence band edges, and the light blue region highlights the energy range between them. **b** Density functional theory (DFT)-calculated projected density of states (DOS) for ML-Si_2_Te_2_ in ML-Si_2_Te_2_/ML-HfTe_2_ and total DOS of bulk-HfTe_2_. The red arrows indicate two peak features in the calculated DOS of ML-Si_2_Te_2_ on ML-HfTe_2_.

We investigated the electronic structure of strain-free ML-Si_2_Te_2_ by combining STS with DFT calculations. Figure 4a presents representative d*I*/d*V* spectra acquired on ML-Si_2_Te_2_ and on the HfTe_2_ substrate. The ML-Si_2_Te_2_ spectrum is characterized by two prominent peaks separated by ~ 1.25 eV (marked by red arrows). As shown in Fig. 4b, DFT calculations reproduce this feature, albeit with an overall energy shift of ~ 0.25 eV. This shift is attributed to a substrate-induced doping effect which is not fully captured by DFT^22,29^. The good agreement between the experimental and theoretical results confirms the formation of a well-defined band structure in the synthesized ML-Si_2_Te_2_ islands.

Since a bulk band gap is essential for the QSH phase^13,22^, the experimental confirmation of the band gap in ML-Si_2_Te_2_ grown on HfTe_2_—predicted theoretically in Fig. 2a—is of particular importance. In Fig. 4a, the red curve exhibits a smooth, low-intensity region spanning from  − 100 to 300 meV, indicating the presence of a sizable band gap in ML-Si_2_Te_2_. However, the exact energy positions of the conduction and valence band edges appear smeared due to the metallic HfTe_2_ substrate. To better resolve the band edges, we acquired high-resolution d*I*/d*V* spectra on ML-Si_2_Te_2_ (see inset in Fig. 4a and Supplementary Fig. 12c), from which the VBM (≈ − 10 meV) and CBM (≈ 290 meV) can be readily identified (gray dashed lines), defining a band gap of ≈ 300 meV. This value is consistent with the DFT-calculated gap of 319 meV (Fig. 2a) and corroborated by systematic measurements on 20 ML-Si_2_Te_2_ islands (See Supplementary Fig. 13). The sizable bulk gap in ML-Si_2_Te_2_ suggests that the underlying nontrivial band topology is likely to remain robust against thermal excitations at room temperature.

### Topological edge states in ML-Si_2_Te_2_

**Fig. 5:**
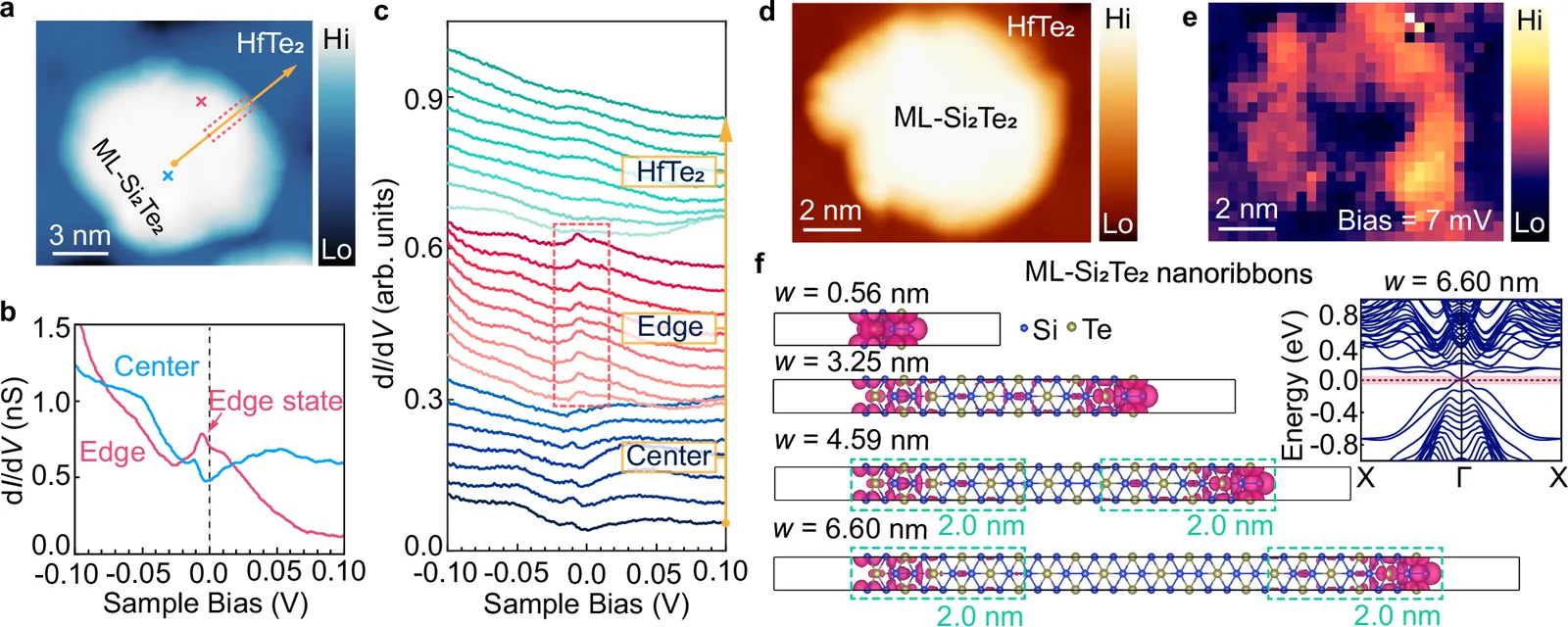
Identification of the edge states. **a** STM image (*V* = − 1.0 V, *I* = 10 pA) of a typical monolayer Si_2_Te_2_ (ML-Si_2_Te_2_) island. **b** d*I*/d*V* spectra (*V*_stab_ = − 0.1 V, *I*_stab_ = 100 pA, *V*_mod_ = 2.0 mV) measured at the edge (red) and center (blue) of the island (positions marked by crosses in (**a**). **c** d*I*/d*V* spectra recorded along the orange arrow in (**a**) with 0.36 nm spacing. Spectra are vertically offset for clarity. Measurement positions of edge spectra are marked by a red dashed rectangle in (**a**). **d** STM image (*V* = 1.0 V, *I* = 10 pA) of another ML-Si_2_Te_2_ island. **e** d*I*/d*V* map (*V*_stab_ = 0.2 V, *I*_stab_ = 100 pA, *V*_mod_ = 2.0 mV) acquired at +7 mV. **f** Right: DFT-calculated band structure of a free-standing ML-Si_2_Te_2_ nanoribbon with a width of *w* = 6.60 nm. The pink shading highlights the band dispersion within the energy window *E* = *E*_F_ ± 50 meV. Left: real-space charge-density distributions (shown as magenta isosurfaces) of the topological edge states within the same energy window for ribbons with widths of *w* = 0.56, 3.25, 4.59, and 6.60 nm. The isosurface value was set to 0.0001 *e*/Å^3^. For *w* = 4.59 and 6.60 nm, the green boxes mark the 2.0 nm spatial extent of edge-state charge density from each ribbon edge. SOC is included in all calculations.

A hallmark of QSH systems is the presence of topological edge states within the bulk band gap, which manifest as enhanced d*I*/d*V* intensity at step edges^13,22,42–45^. Figure 5a, b show representative d*I*/d*V* spectra acquired at the step edge (red curve) and the center (blue) of a ML-Si_2_Te_2_ island. The center spectrum exhibits a V-shaped dip between  ± 50 mV with a weak shoulder at about −10 meV. In contrast, the edge spectrum displays a pronounced peak at *E*_F_, providing direct evidence of in-gap edge states. These features are reproducibly observed across multiple ML-Si_2_Te_2_ islands and step geometries; see Supplementary Fig. 14, indicating their robust nature.

To examine the nature of the observed edge-state feature, we acquired a series of d*I*/d*V* spectra along a line spanning from the island center to the HfTe_2_ substrate, crossing the step edge (Fig. 5c). Spectra obtained at the island center exhibit a minimum around *E*_F_. As the measurement position approaches the step edge, this minimum remains essentially unchanged (blue curves) until it is overtaken by a pronounced peak located slightly below *E*_F_ that emerges within approximately 2.0 to 3.0 nm of the edge (red curves) and vanishes on the substrate side (green curves). As discussed in Supplementary Information, the peak-like spectral feature is compatible with the predicted edge-state electronic structure of ML-Si_2_Te_2_, with possible contributions from the momentum-dependent tunneling scenario and van-Hove-singularity-like effects associated with the edge-state dispersion. We note that the weak shoulder feature near −10 meV observed in Fig. 5b is also present in the spectra acquired at the island center in Fig. 5c. As shown in Fig. 2a, the projected band structure of ML-Si_2_Te_2_ on HfTe_2_ reveals that the substrate contributes intrinsic electronic states near *E*_F_, including the valence band maximum at the *Γ* point and a relatively flat band near the *M* point. These substrate-derived states may contribute to the weak shoulder feature observed near  −10 meV, suggesting a possible substrate-related origin.

Further insight into the spatial localization of the edge-state feature is provided by 2D d*I*/d*V* mapping. Figure 5d shows another representative ML-Si_2_Te_2_ island. As shown in Supplementary Figs. 15 and 16, the spectra acquired at both the island center and the island boundaries exhibit the same overall characteristics as those shown in Fig. 5b,c. For this island, the edge-state peak is located at approximately +7 mV. Correspondingly, the d*I*/d*V* map acquired at +7 mV reveals a pronounced boundary-localized feature along the island edge (Fig. 5e). The spatial extent of the edge-state distribution is estimated to be 1.5 − 2.0 nm. Similar boundary-localized features are reproduced in d*I*/d*V* maps acquired at nearby energies (Supplementary Fig. 17), consistent with the edge-state feature extending over a finite energy range around the peak position. At +50 mV, well away from the edge-state peak, the boundary-localized feature becomes increasingly obscured by the growing contribution of metallic states from the HfTe_2_ substrate. The local suppression of signal intensity at the bottom part of the island may reflect variations in local tunneling conditions^45,46^ and residual tip-related effects (see Supplementary Information for details).

The extended spatial profile of the edge-state features is consistent with the DFT-calculated charge-density distribution of the topological edge states in ML-Si_2_Te_2_ nanoribbons. As shown in Supplementary Figs. 18, 19, and Fig. 5f, finite-size-induced inter-edge hybridization prevents the formation of a gapless Dirac-cone dispersion for ribbon widths below 6.6 nm. By contrast, a clear Dirac-cone-like crossing develops at the *Γ* point for *w *≥ 6.60 nm while remaining energetically separated from bulk-derived states near *E*_F_. As expected, the charge-density distributions of narrow nanoribbons (*w* = 0.56 and 3.25 nm) exhibit substantial overlap between opposite edges, indicating strong inter-edge coupling. By contrast, for *w* = 4.59 and 6.60 nm, the Dirac-cone-related edge states within *E* = *E*_F_ ± 50 meV remain localized near the ribbon boundaries, with a spatial extent of  ≈ 2.0 nm and negligible charge density at the ribbon center. This localization length agrees well with the experimentally observed value of 1.5 − 2.0 nm in ML-Si_2_Te_2_ islands. Notably, clear boundary-localized edge states already emerge for *w* = 4.59 nm, despite the presence of a finite-size gap in the Dirac-cone dispersion (Supplementary Fig. 18), indicating that the well-defined edge-state features can remain observable even in relatively small ML-Si_2_Te_2_ islands.

Together, our STM/STS measurements and DFT calculations show that the boundary-localized states in ML-Si_2_Te_2_ exhibit key characteristics expected for topological edge states in QSH materials. We note, however, that although these states show a characteristic spatial extent and appear along edge segments with different local geometries, their protection by TRS has not been directly established in the present work. Further experiments, for example transport measurements, will be important for directly testing this protection. Notably, the DFT calculations show that the topological edge states remain energetically separated from bulk-derived states near *E*_F_ (Supplementary Fig. 18), while electron-phonon coupling (EPC) calculations indicate relatively weak EPC in ML-Si_2_Te_2_ (Supplementary Fig. 20). These results suggest that transport measurements may provide a particularly promising route for probing the intrinsic properties of the topological edge states. The successful realization of strain-free ML-Si_2_Te_2_ provides a promising platform for such studies.

In summary, by combining high-throughput computational screening with band structure calculations, we identified HfTe_2_ as a suitable substrate for the epitaxial growth of ML-Si_2_Te_2_, while preserving its predicted topological characteristics. Subsequent MBE successfully yielded strain-free (1 × 1) ML-Si_2_Te_2_ on HfTe_2_ substrates with vdW-type interfacial interactions. Using STM/STS, we observed spectroscopic signatures of a sizable bulk gap together with distinct edge-state features, consistent with the predicted topological electronic structure of ML-Si_2_Te_2_. As ML-Si_2_Te_2_ is an artificial 2D material without a bulk analog, our findings pave the way for further investigations into its topological electronic properties and potential applications in spintronic devices. More broadly, this work highlights the potential of a material-by-design approach in the study of 2D materials and heterostructures.

## Methods

### Density functional theory calculations

Density functional theory (DFT) calculations were performed using the Vienna ab initio simulation package (VASP)^36^ with the projector augmented wave (PAW) method^47,48^. Calculation models and results were processed via the ALKEMIE platform^49^. The generalized gradient approximation (GGA) in the form of the Perdew, Burke, and Ernzerhof (PBE) functional^50^ was used to treat the exchange-correlation functional. A plane-wave cutoff energy of 600 eV was employed, and the Brillouin zone was sampled using a *Γ*-centered 11 × 11 × 1 Monkhorst-Pack **k**-point mesh^51^. All structures were fully optimized until the total energy and atomic forces converged to 10^−5^ eV and 0.01 eV/Å, respectively. To minimize spurious interactions between adjacent layers, a vacuum layer of 30 Å was introduced, and long-range van der Waals (vdW) interactions were accounted for using the DFT-D3 method^52^.

Phonon spectra of the ML-Si_2_Te_2_/ML-HfTe_2_ heterostructure were obtained using density functional perturbation theory (DFPT) calculations implemented in Phonopy^53^. The thermodynamic stability of the heterostructure was evaluated via ab initio molecular dynamics (AIMD) simulations within the canonical ensemble using a Nose-Hoover thermostat (NVT)^37^. To achieve accurate electronic structure calculations, the Heyd-Scuseria-Ernzerhof (HSE06) hybrid functional was employed to determine the band gaps^38^.

The “spectral density of the edge states” in Fig. 2b refers to the edge spectral function calculated using WannierTools^39,40^. Specifically, a tight-binding Hamiltonian was constructed from maximally localized Wannier functions, and then the edge state spectrum was obtained via the iterative Green’s function method. To ensure the reliability of the Wannier representation, we constructed maximally localized Wannier functions (MLWFs) from physically motivated atomic orbitals. For free-standing ML-Si_2_Te_2_, the Bloch states were projected onto the *s* and *p* (*p*_*x*_, *p*_*y*_, and *p*_*z*_) orbitals of Si and Te atoms, yielding 32 MLWFs in total (including SOC). For the ML-Si_2_Te_2_/ML-HfTe_2_ heterostructure, 58 MLWFs were constructed by including Hf-*d* orbitals (${d}_{{z}^{2}}$, *d*_*xz*_, *d*_*yz*_, ${d}_{{x}^{2}-{y}^{2}}$, and *d*_*xy*_), together with the relevant *s* and *p* orbitals of Si and Te atoms to account for the hybridized interfacial bands. While an 11 × 11 × 1 grid was used for the initial Wannierization, the WCC evolution was calculated using dense sampling of 101 **k**-point strings to ensure smooth Wannier-center evolution and numerical stability (see Supplementary Information for more details).

### Synthesis and characterization of HfTe_2_ single crystals

HfTe_2_ single crystals were grown by the chemical vapor transport (CVT) method. Stoichiometric amounts of high-purity Hf (99.5%) and Te (99.9999%) powders were mixed and sealed in an evacuated quartz tube, with iodine (*I*_2_, 99.999%) at a concentration of 5 mg/ml serving as the transport agent. A temperature gradient of 900 ^∘^C (source) to 800 ^∘^C (sink) was maintained during crystal growth. After two weeks, large, flat, golden single crystals of HfTe_2_ were obtained (see Supplementary Fig. 7).

### Scanning tunneling microscopy/spectroscopy measurements

Scanning tunneling microscopy/spectroscopy (STM/STS) measurements were performed at 5.8 K using an ultrahigh vacuum (UHV) home-built low-temperature STM system (*p* < 9 × 10^−11 ^Torr). Electrochemically etched W-tips coated with gold were used for the measurements. Differential tunneling conductance (d*I*/d*V*) spectra were acquired using the lock-in technique in open feedback mode, with a modulation voltage (*V*_mod_) at a frequency of 987.5 Hz. STM image processing was performed using the WSxM software^54^.

### Epitaxial growth of ML-Si_2_Te_2_ on HfTe_2_

Monolayer Si_2_Te_2_ was fabricated by co-evaporating high-purity Si (99.999%) and Te (99.9999%) onto the HfTe_2_ substrates held at 200 ^∘^C, followed by post-annealing at 385 ^∘^C for 5 min. Si evaporation was carried out using a commercial e-beam evaporator (FOCUS), while Te was evaporated from a standard Knudsen cell (CREATEC). During growth, the Si flux, monitored at 9 nA, was significantly lower than the Te flux, with the Te source temperature maintained at 270 ^∘^C, ensuring a Te–to–Si flux ratio exceeding 20 : 1. Excess Te that did not react with Si desorbed from the heated HfTe_2_ substrate, so that the nominal growth rate was determined by the Si flux (approximately 1ML/hour). During post-annealing, the Te source temperature was raised to 320 °C, and the Si flux was increased to 70 nA to compensate for the decomposition of SiTe_*x*_.

## Supplementary information

Supplementary Information

Peer Review file

## Source data

Source Data

## Data Availability

The source data generated in this study have been deposited in the WueData research data repository of the University of Würzburg and are available at https://doi.org/10.58160/a21cayaxuk0rzq02. Source data are provided with this paper.
