# Supplementary material for: Experimental realization of the topologically nontrivial phase in monolayer Si2Te2
Source: Nat Commun. 2026 Sep 11;17:9741. doi: 10.1038/s41467-026-77669-9 (PMC13569488; doi:10.1038/s41467-026-77669-9)
Supplement: Supplementary file 1 [file 41467_2026_77669_MOESM1_ESM.pdf]

Supplementary Information for:
Experimental realization of the topologically nontrivial phase in monolayer Si_2Te_2

Xiaochun Huang[†,*,1] Lingxiao Zhao[†,2] Rui Xiong[*,3] Wenbin Li,[4,5]
Bao-Tian Wang,[6,7] Baisheng Sa[*,3] and Matthias Bode[1,8]

[1] *Physikalisches Institut, Experimentelle Physik II, Universität Würzburg, Am Hubland, 97074 Würzburg, Germany*

[2] *Quantum Science Center of Guangdong-Hong Kong-Macao Greater Bay Area (Guangdong), Shenzhen, China*

[3] *Multiscale Computational Materials Facility, Key Laboratory of Eco-materials Advanced Technology, College of Materials Science and Engineering, Fuzhou University, Fuzhou, PR China*

[4] *The Institute for Solid State Physics, The University of Tokyo; Chiba 277-8581, Japan*

[5] *Present Address: Department of Chemistry, National University of Singapore, Singapore 117543, Singapore.*

[6] *Institute of High Energy Physics, Chinese Academy of Sciences, Beijing 100049, China*

[7] *Spallation Neutron Source Science Center, Dongguan 523803, China*

[8] *Wilhelm Conrad Röntgen-Center for Complex Material Systems (RCCM), Universität Würzburg, Am Hubland, 97074 Würzburg, Germany*

(Dated: August 15, 2026)

Exfoliation energy of $HfTe_2$ and $NiTe_2$

The exfoliation energy of $HfTe_2$ and $NiTe_2$ monolayers was calculated from a 6-layer (6L) slab of $HfTe_2$ and $NiTe_2$, respectively. The exfoliation energy is defined as:

$$E_{\mathrm{exf}} = \frac{E_{\mathrm{6L}} - E_{(6-1)\mathrm{L}}}{A}, \tag{S1}$$

where E_{6L} is the total energy of the 6-layer slab, $E_{(6-1)\mathrm{L}}$ is the total energy of the system after a monolayer is exfoliated from the remaining slab by a certain distance, and A is the cross-sectional area of the slab. As shown in Fig. S1, $HfTe_2$ exhibits a significantly lower exfoliation energy than $NiTe_2$ over the entire range of $(d - d_0)$, indicating that $HfTe_2$ can be more easily cleaved than $NiTe_2$.

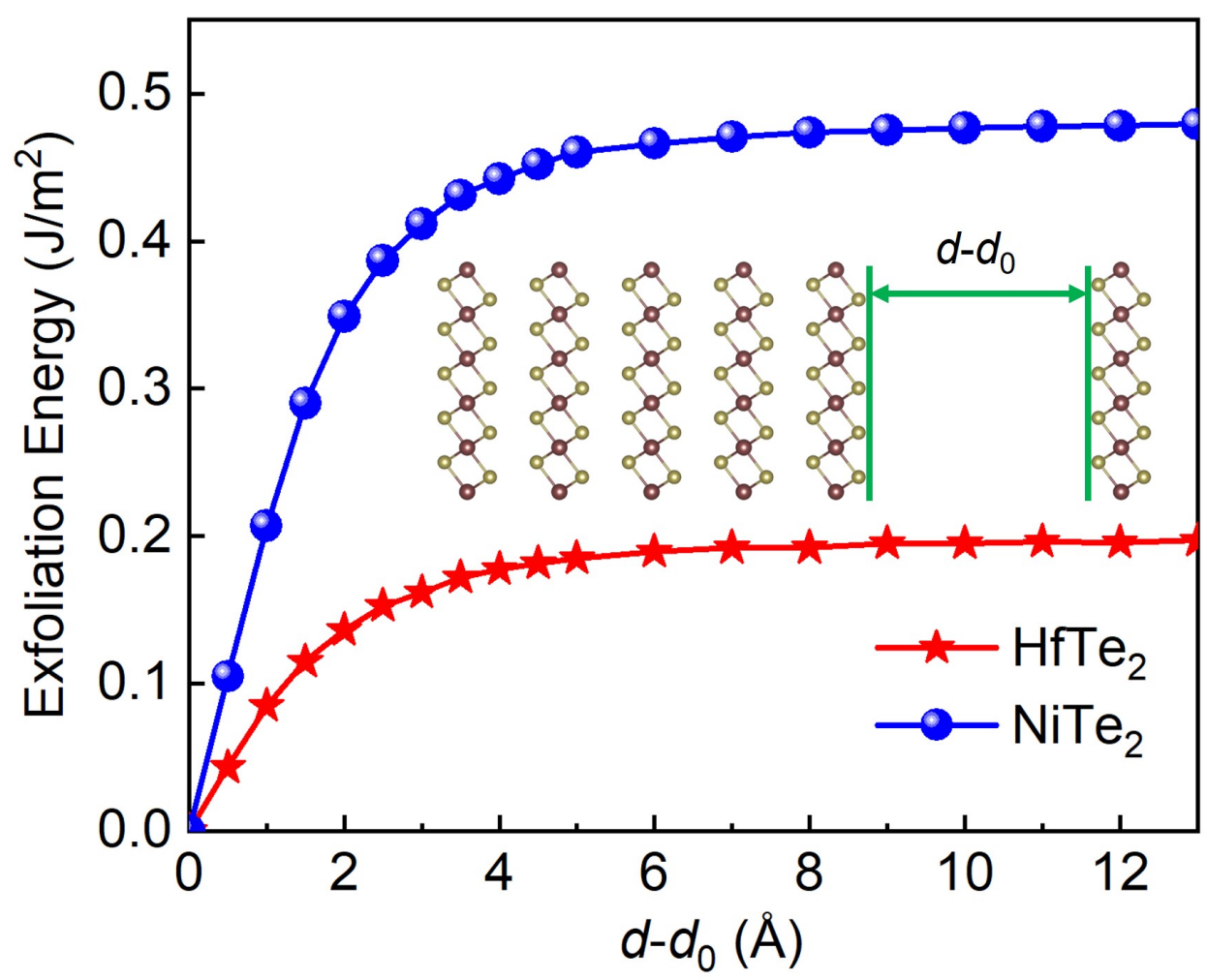

FIG. S1. Main panel: DFT-calculated exfoliation energy for $HfTe_2$ and $NiTe_2$. Inset: Schematic of the interlayer spacing parameter $(d - d_0)$, where d_0 is the equilibrium interlayer distance.

van der Waals interaction between ML-Si_2Te_2 and ML-$HfTe_2$

The interfacial interaction between ML-Si_2Te_2 and ML-$HfTe_2$ is quantitatively evaluated by the binding energy, defined as:

$$E_b = \frac{E_{Si_2Te_2/HfTe_2} - E^{fix}_{Si_2Te_2} - E^{fix}_{HfTe_2}}{A}, \tag{S2}$$

where $E_{Si_2Te_2/HfTe_2}$ is the total energy of the ML-Si_2Te_2/ML-$HfTe_2$ heterostructure, $E^{fix}_{Si_2Te_2}$ and $E^{fix}_{HfTe_2}$ are the total energies of the isolated ML-Si_2Te_2 and ML-$HfTe_2$ respectively, with their lattice constants constrained to those of the heterostructure. A denotes the area of the two-dimensional (2D) unit cell. According to Eq. S2, the calculated binding energy between ML-Si_2Te_2 and ML-$HfTe_2$ is -0.296 eV/unit cell, indicating a typical vdW interaction [S1].

AIMD simulations of a ML-Si_2Te_2/ML-$HfTe_2$ heterostructure at 300 K for 100 ps

We performed *ab initio* molecular dynamics (AIMD) simulations of a ML-Si_2Te_2/ML-$HfTe_2$ heterostructure at 300 K for 100 ps (Fig. S2), which yielded results consistent with those shown in Fig. 1e, with no apparent structural reconstruction or degradation observed throughout the simulation time.

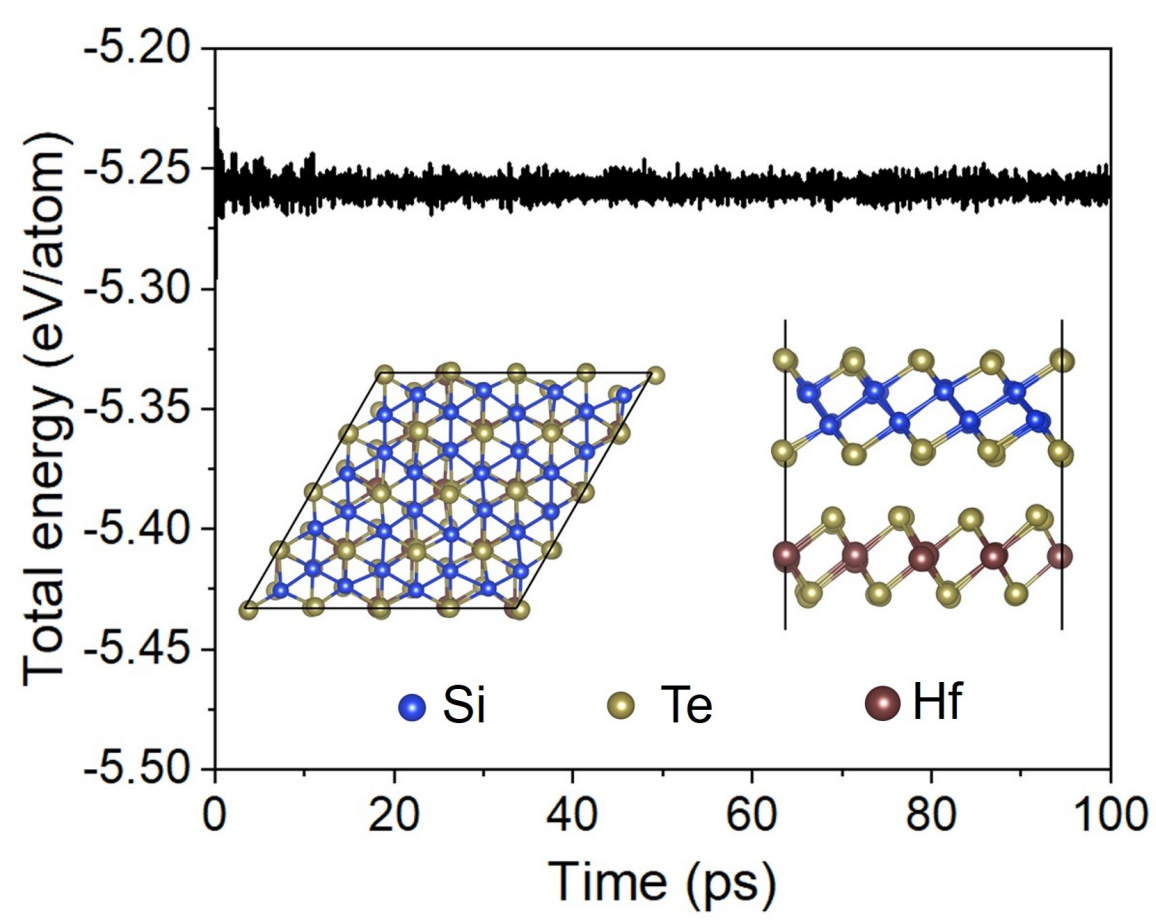

FIG. S2. Time evolution of the total energy during AIMD simulations of the ML-Si_2Te_2/ML-$HfTe_2$ heterostructure using a 4×4 supercell. The simulations were performed for 100 ps with a time step of 1 fs. Insets: Structural snapshots of a heterostructure after 100 ps of AIMD simulation.

DFT-calculated band structures of free-standing ML-Si_2Te_2 and the ML-Si_2Te_2/ML-$HfTe_2$ heterostructure

The band structures of free-standing ML-Si_2Te_2 and the ML-Si_2Te_2/ML-$HfTe_2$ heterostructure were calculated using the Heyd–Scuseria–Ernzerhof (HSE06) hybrid functional, both with and without spin-orbit coupling (SOC). As shown in Fig. S3a,b, the SOC induces a band inversion at the Γ point in free-standing ML-Si_2Te_2, consistent with previous studies [S2–S4]. A comparison between Fig. S3a and c reveals that the projected band dispersion of ML-Si_2Te_2 in the heterojunction (without SOC) closely resembles that of the free-standing ML-Si_2Te_2. The same conclusion holds when SOC is included (see Fig. S3b and Fig. 2a).

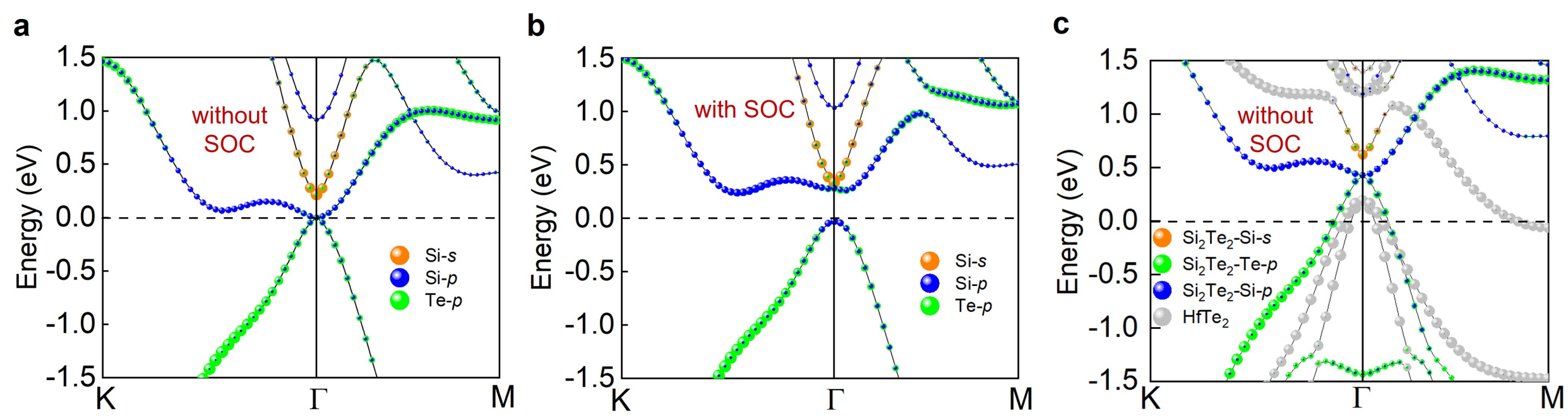

FIG. S3. The DFT-calculated band structures. **a**,**b**, Decomposed band structure of free-standing ML-Si_2Te_2 without (**a**) and with (**b**) spin-orbit coupling (SOC). **c**, Decomposed band structure of ML-Si_2Te_2/ML-$HfTe_2$ without SOC. Solid lines represent the calculated band dispersion. Orange, green, and blue symbols denote Si-*s*, Te-*p*, and Si-*p* orbital contributions from ML-Si_2Te_2, respectively, while gray symbols indicate contributions from $HfTe_2$. The size of the colored spheres in (**a**), (**b**) and (**c**) represents the orbital weight.

SOC-induced band inversion in free-standing ML-Si_2Te_2 obtained from HSE and GW calculations

In heavy-element telluride systems, the band ordering and topological properties can be sensitive to the choice of exchange-correlation functional. To examine the robustness of our conclusions, we performed GW calculations for free-standing ML-Si_2Te_2 and compared the band evolution obtained from both HSE and GW calculations with and without SOC

(Fig. S4). In both approaches, the same SOC-driven band inversion is found at the Γ point. Specifically, without SOC, the conduction- and valence-band-edge states are energetically close and show strong hybridization between Te-p and Si-p orbitals. Upon including SOC, the relative band ordering and orbital character near the band edges evolve in a qualitatively identical manner in both HSE and GW calculations, indicating that the nontrivial topological band inversion is robust against the choice of exchange-correlation functional within the present level of theory. Moreover, the HSE results are consistent with a previous theoretical study of ML-Si_2Te_2 [S3].

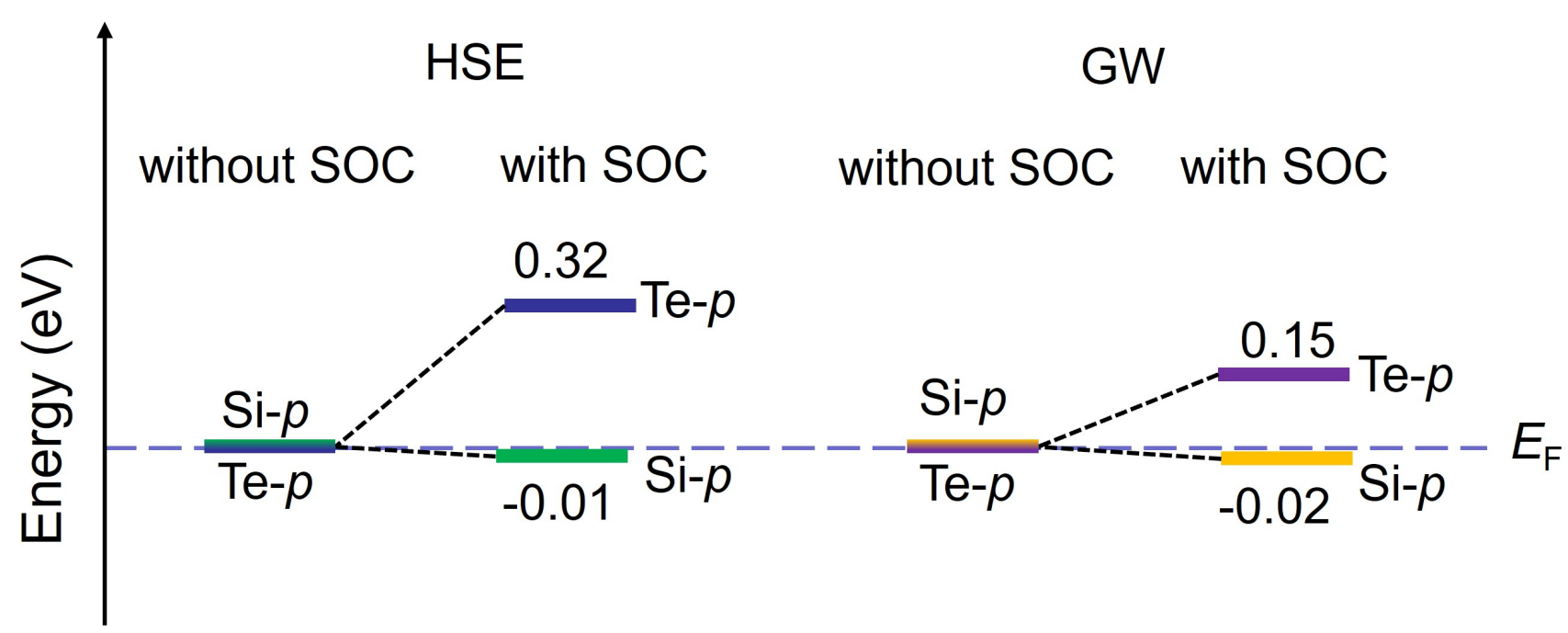

FIG. S4. Schematic illustration of the spin-orbit coupling (SOC)-induced band evolution and band inversion at the Γ point in free-standing ML-Si_2Te_2, as obtained from (Heyd–Scuseria–Ernzerhof) HSE and GW calculations. The dashed line indicates the Fermi level (E_F). Colored bars denote the dominant orbital character (Te-p and Si-p) of the band-edge states.

Electronic work functions of ML-Si_2Te_2 and ML-$HfTe_2$

The electronic work function φ is determined by the energy difference between the Fermi energy (E_F) and the vacuum potential (E_{vac}), and is given by:

$$\varphi = E_{vac} - E_F, \tag{S3}$$

The calculated work functions of ML-Si_2Te_2 and ML-$HfTe_2$ are 3.93 eV and 4.49 eV, respectively. When the ML-Si_2Te_2 and ML-$HfTe_2$ come into contact to form the ML-Si_2Te_2/ML-$HfTe_2$ heterostructure, the difference in work functions facilitates the migration of free holes from ML-$HfTe_2$ to ML-Si_2Te_2 until the E_F reach equilibrium.

Edge states and Wannier charge centers in the ML-Si_2Te_2/ML-$HfTe_2$ heterostructure and free-standing ML-Si_2Te_2

In this work, the Wannier charge center (WCC) evolution was calculated using the WannierTools package based on the tight-binding Hamiltonian constructed from Wannier90 [S5, S6]. Based on the validated Wannier representation (see following), the evolution of the WCC for the ML-Si_2Te_2/ML-$HfTe_2$ heterostructure and free-standing ML-Si_2Te_2 is studied (Fig. S5a and c). As shown, the WCC curves cross any arbitrary horizontal reference line an odd number of times, confirming $Z_2 = 1$ for both systems. Furthermore, the Dirac-cone-like band structure of the one-dimensional (1D) topological edge state of free-standing ML-Si_2Te_2 is visualized in Fig. S5b.

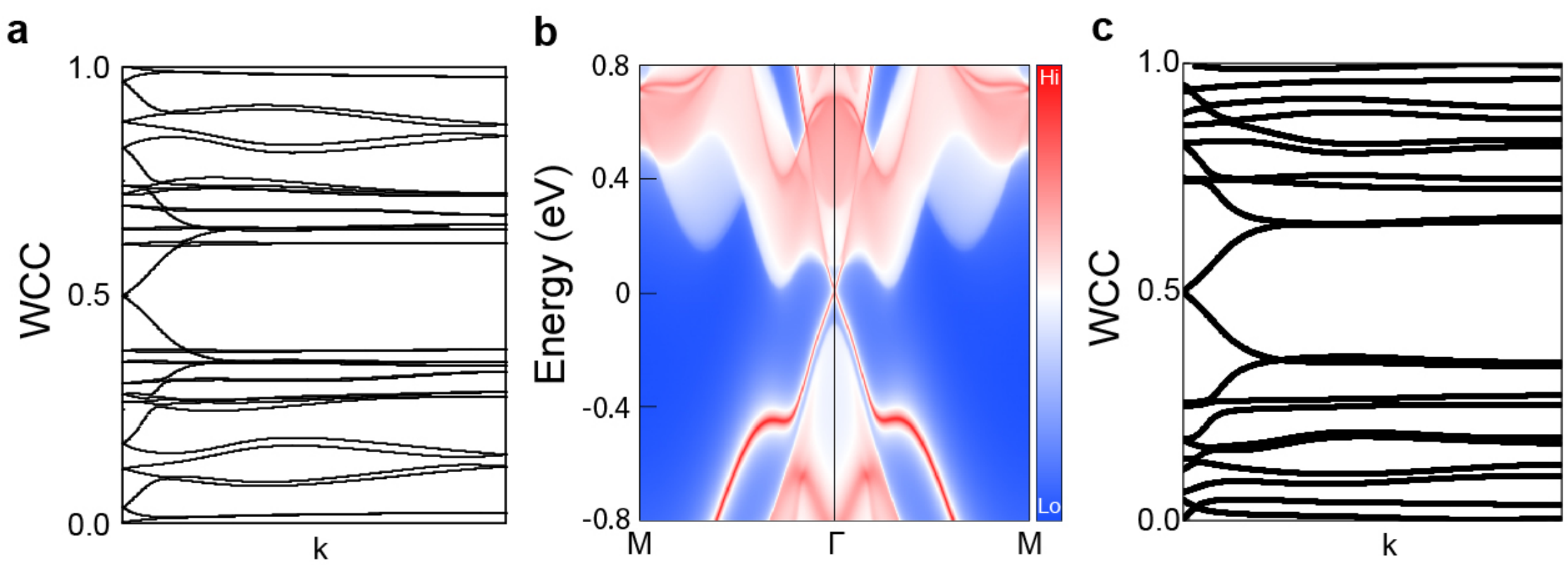

FIG. S5. The DFT-calculated Wannier charge centers (WCCs) and edge states with SOC. **a**, Evolution of the WCCs for the ML-Si_2Te_2/ML-$HfTe_2$ heterostructure. **b**, Spectral density of the edge states of free-standing ML-Si_2Te_2. The color scale represents the spectral density. **c**, Evolution of the WCCs for free-standing ML-Si_2Te_2.

The reliability of the WCC evolution and the resulting topological invariant (Z_2) depends on the quality of the Wannier representation and the convergence of the relevant computational parameters. To ensure the reliability of the Wannier representation, we constructed maximally localized Wannier functions (MLWFs) from physically motivated atomic orbitals. For free-standing ML-Si_2Te_2, the Bloch states were projected onto the s and p (p_x, p_y, and p_z) orbitals of Si and Te atoms, yielding 32 MLWFs in total (including SOC). These orbitals accurately capture the valence and conduction bands associated with the topological band inversion. The resulting MLWFs showed good localization quality, with Wannier centers

closely aligned with the atomic positions and stable spreads after convergence. Regarding the energy windows, the frozen window was chosen from -17.8 eV to 0.6 eV, ensuring that all occupied states together with the inverted energy manifold were faithfully reproduced by the Wannier interpolation. The outer disentanglement window was extended to 7.8 eV to provide a sufficiently large Hilbert space for the projection. For the ML-Si_2Te_2/ML-$HfTe_2$ heterostructure, 58 MLWFs were constructed by including Hf-d orbitals (d_{z^2}, d_{xz}, d_{yz}, $d_{x^2-y^2}$, and d_{xy}), together with the relevant s and p orbitals of Si and Te atoms to account for the hybridized interfacial bands. The frozen window (-15.60 eV to 2.25 eV) was carefully chosen to cover both the hybridized bands and the inverted states near E_F. Furthermore, we confirmed that the calculated Z_2 index is well-converged with respect to the **k**-point sampling. While an $11 \times 11 \times 1$ grid was used for the initial Wannierization, the WCC evolution was calculated using dense sampling of 101 **k**-point strings to ensure smooth Wannier-center evolution and numerical stability.

We examined the accuracy of the Wannier interpolation by comparing the interpolated bands with the directly calculated HSE+SOC band structures, as shown in Fig. S6. The interpolated bands (red dashed lines) show excellent agreement with the directly calculated HSE+SOC band structures (black circles) within the energy window from -3.0 to 3.0 eV, confirming the reliability of the Wannier representation for the subsequent topological analysis.

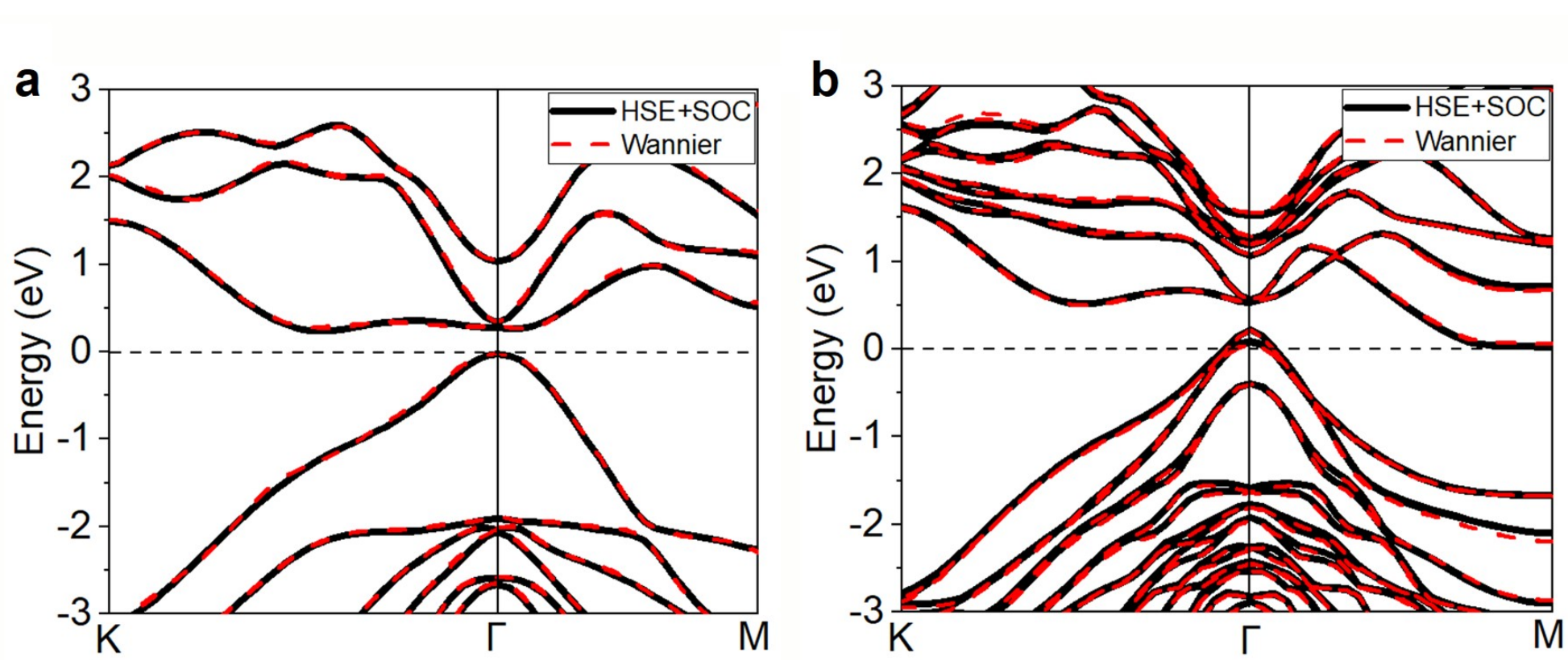

FIG. S6. Comparison of the calculated electronic band structures for (**a**) free-standing ML-Si_2Te_2 and (**b**) the ML-Si_2Te_2/ML-$HfTe_2$ heterostructure. SOC is included in all calculations. The black solid lines represent the direct HSE06 band structures, while the red dashed lines denote the Wannier-based tight-binding interpolated bands. The horizontal dashed lines indicate the E_F.

High-quality $HfTe_2$ single crystals

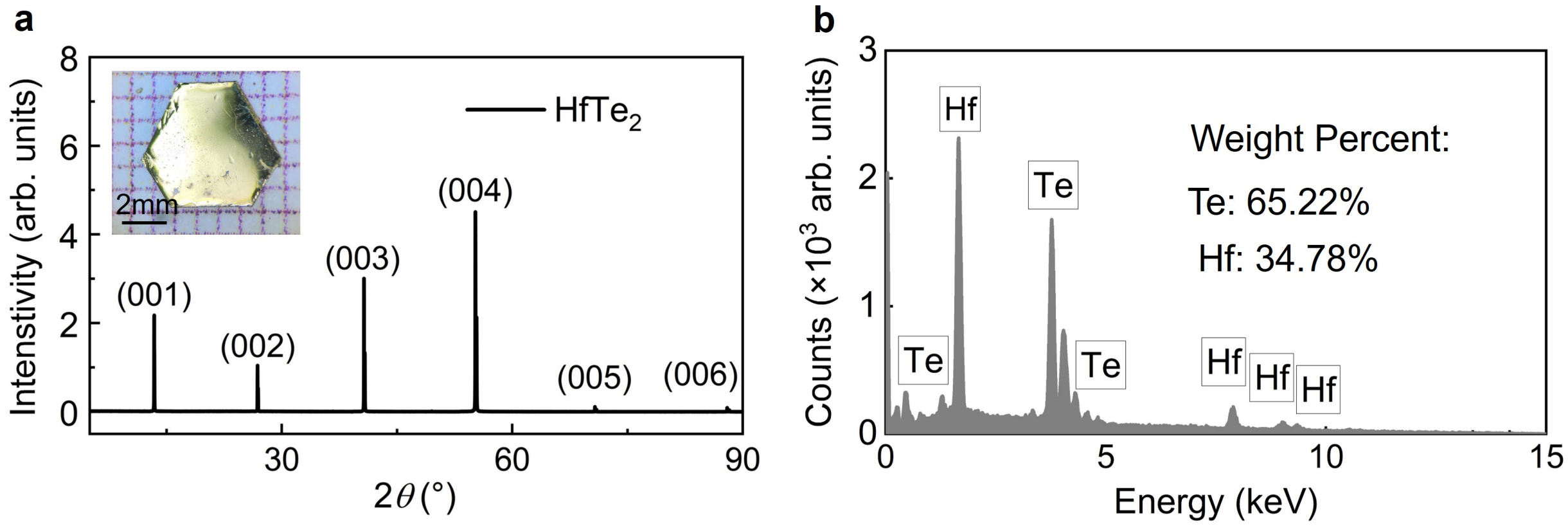

FIG. S7. High-quality $HfTe_2$ single crystal. **a**, Main panel: XRD pattern of $HfTe_2$ single crystal, showing only the $(00l)$ reflections. Inset: Optical image of a typical $HfTe_2$ sample with a lateral size of several millimeters. **b**, EDS measurement of a typical $HfTe_2$ single crystal.

Preparation and characterization of atomically clean $HfTe_2$ surfaces

$HfTe_2$ single crystals were mounted onto a Mo sample plate using Ta foil (and wire) and cleaved in UHV conditions. Fig. S8 presents both large-scale and atomic-resolution STM images of the as-cleaved $HfTe_2$ substrate, revealing an atomically smooth surface. The experimentally determined in-plane lattice constants ($a_{\mathrm{HfTe_2}} = 394\,\mathrm{pm}$) and step height ($c_{\mathrm{HfTe_2}} = 666\,\mathrm{pm}$) are in good agreement with previously reported values ($a_{\mathrm{HfTe_2,ref}} = 395\,\mathrm{pm}$, $c_{\mathrm{HfTe_2,ref}} = 666\,\mathrm{pm}$) [S7].

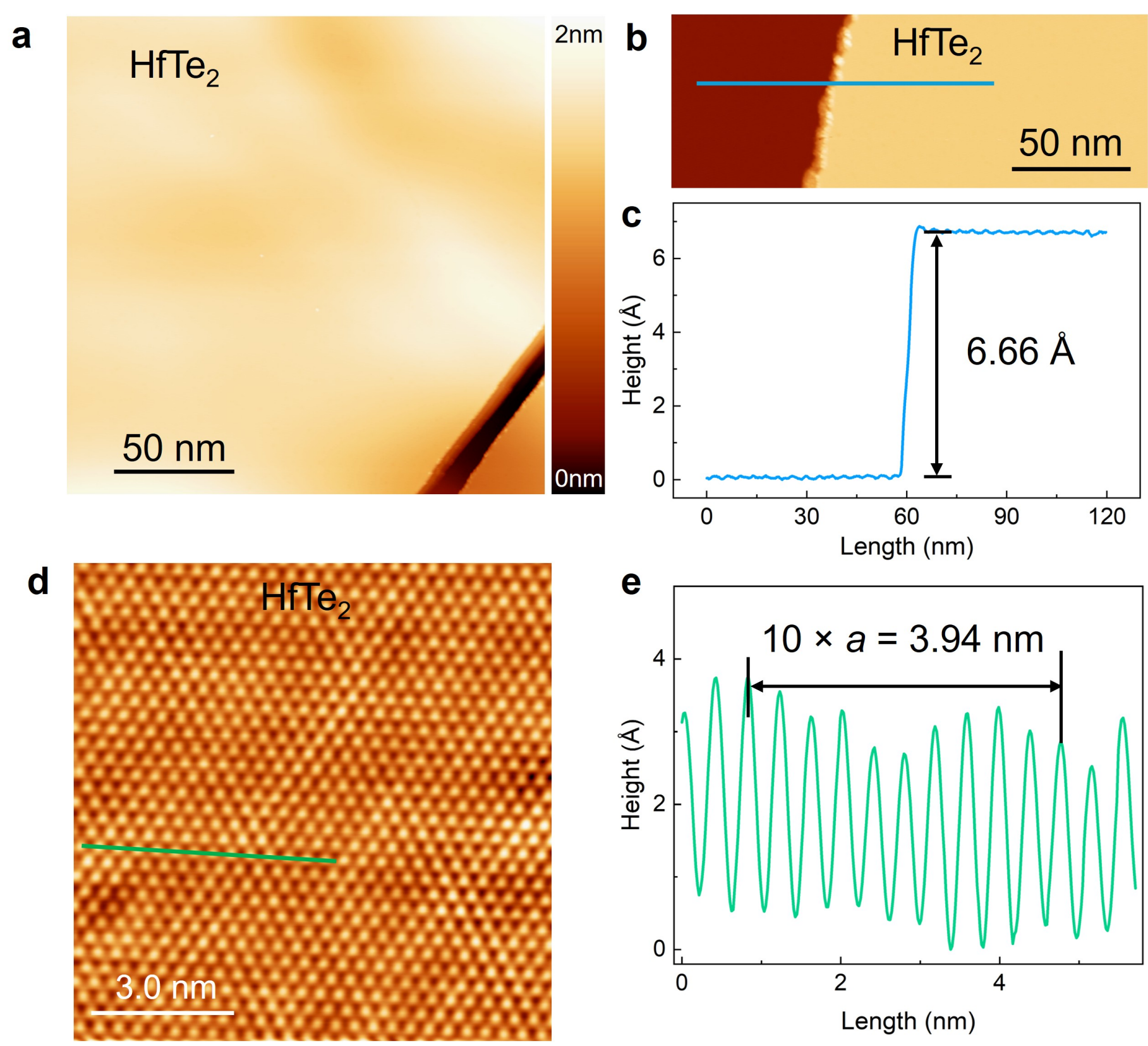

FIG. S8. Morphology of an as-cleaved $HfTe_2$ substrate. **a**, Large-scale STM image ($V = -1.0\,\mathrm{V}$, $I = 10\,\mathrm{pA}$) of $HfTe_2$ substrate. **b**, STM image ($V = -1.0\,\mathrm{V}$, $I = 10\,\mathrm{pA}$) measured at a step edge. **c**, Height profile, taken along the blue line in (b). **d**, Atomic resolution STM image ($V = -0.2\,\mathrm{V}$, $I = 900\,\mathrm{pA}$) of $HfTe_2$ substrate. **e**, Line profile, taken along the green line in (**d**). Here, a denotes the in-plane lattice constant of $HfTe_2$.

The evolution of $SiTe_x$ surface morphology with increasing annealing temperature

Figure S9 illustrates the evolution of $SiTe_x$ surface morphology with increasing annealing temperature from 200 to 385°C. At $T_{\mathrm{ann}} = 200$°C, the Si_2Te_2 film consists of spatially isolated islands on the substrate, with no observable periodic structure, as shown in Fig. S9a and b. Upon increasing the annealing temperature to $320 - 350$°C, short-range periodicity emerges in the STM images (Fig. S9c-e), revealing hexagonally arranged surface atoms in certain regions, although amorphous domains persist. At 385°C, the atomic-resolution STM image (Fig. S9f) exhibits a well-ordered hexagonal lattice, indicating the formation of crystalline Si_2Te_2. Notably, this annealing temperature exceeds that required for crystalline ML-Si_2Te_2 grown on Sb_2Te_3, as reported in our previous study [S4], which can be attributed to differing substrate interactions.

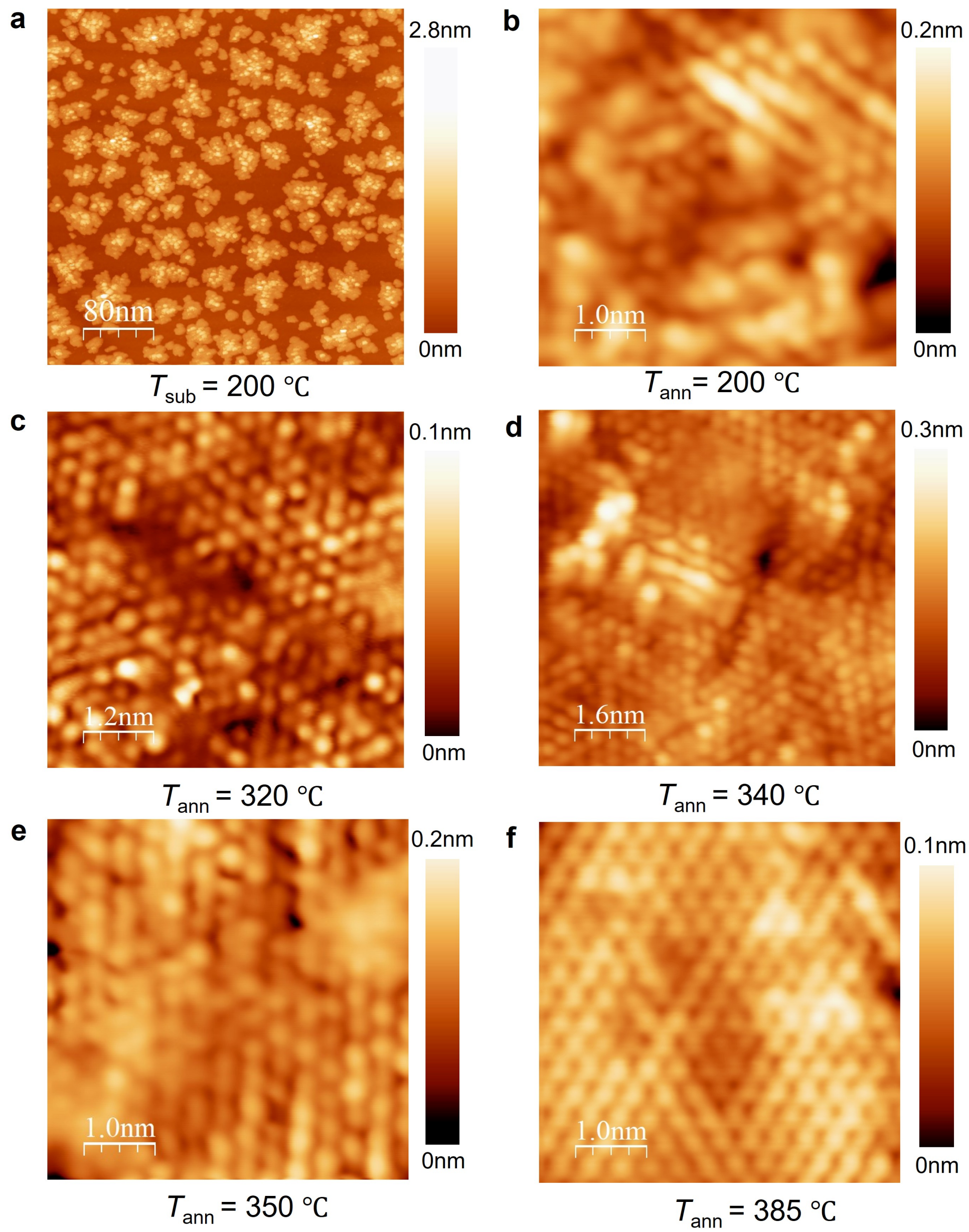

FIG. S9. Co-evaporation of Si and Te on $HfTe_2$ followed by post-annealing. **a**, Large-scale STM image of an as-grown sample annealed at $T_{sub} = 200°C$. **b-f**, Atomic-resolution STM images of samples annealed at progressively higher temperatures T_{ann}. Measurement conditions: (**a**) $V = 1.0\,V$, $I = 100\,pA$; (**b**) $V = 0.2\,V$, $I = 1000\,pA$; (**c**) $V = 0.050\,V$, $I = 1000\,pA$; (**d**) $V = 0.2\,V$, $I = 500\,pA$; (**e**) $V = 0.5\,V$, $I = 100\,pA$; and (**f**) $V = 0.2\,V$, $I = 200\,pA$.

Crystallographic alignment between ML-Si_2Te_2 islands and the $HfTe_2$ substrate

To elucidate the epitaxial relationship between ML-Si_2Te_2 and the $HfTe_2$ substrate, we performed systematic measurements across multiple samples. As shown in Fig. S10, atomic-resolution STM images consistently reveal a well-defined epitaxial relationship, with ML-Si_2Te_2 adopting a hollow-site configuration on the $HfTe_2$ substrate. DFT calculations further support this observation, as shown in Fig. S11. The total energy of the hollow-site configuration (-37.075 eV) is lower than that of the top-site configuration (-36.993 eV), indicating that the hollow-site configuration is energetically more favorable, consistent with our experimental results.

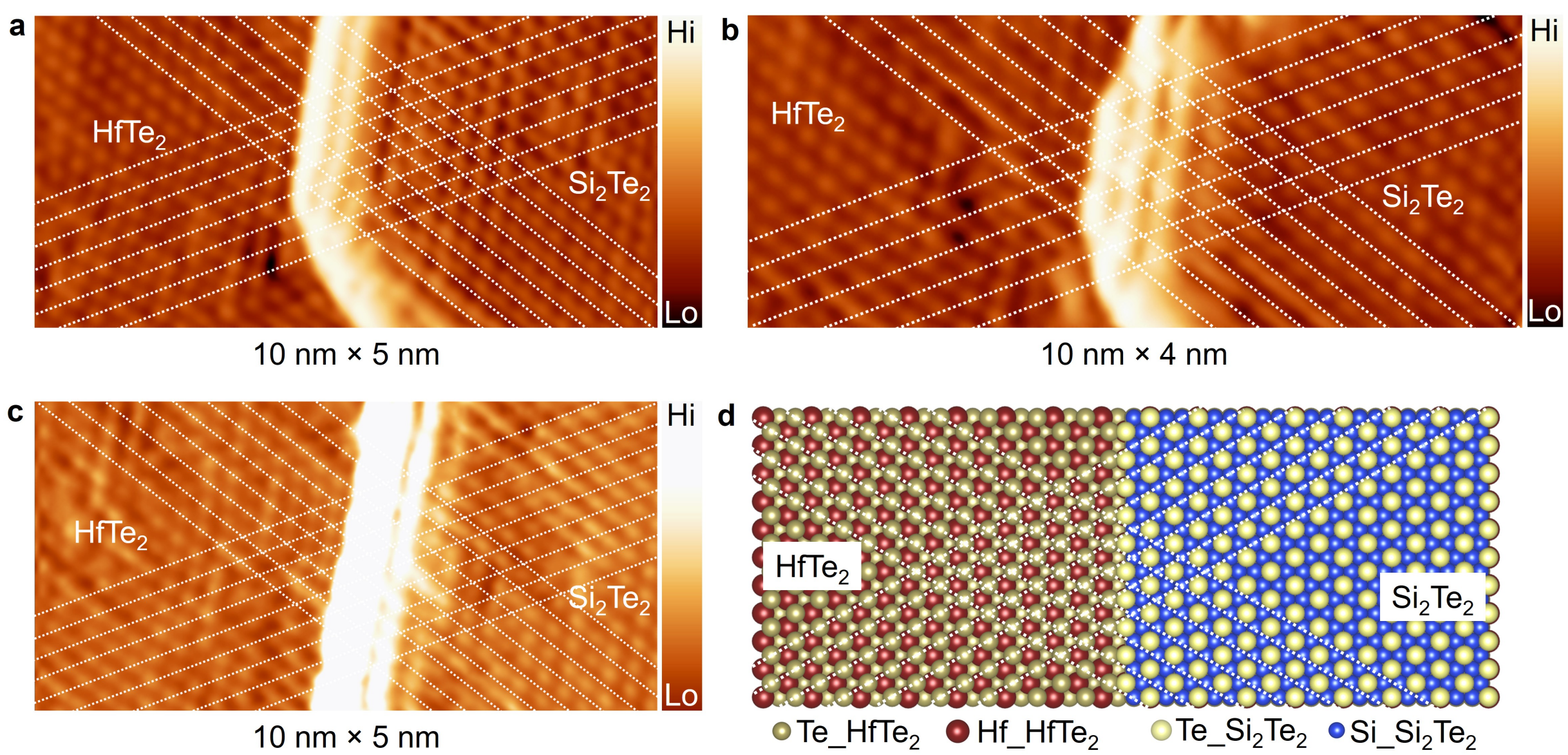

FIG. S10. Experimentally measured crystallographic orientation of ML-Si_2Te_2 islands relative to the $HfTe_2$ substrate. **a-c**, Atomic-resolution STM images $V = 0.5\,\text{V}$, $I = 200\,\text{pA}$ of three different ML-Si_2Te_2 islands, showing the surface lattice structures of both the ML-Si_2Te_2 and the $HfTe_2$ substrate simultaneously. White dashed lines are provided as guides to the eye. **d**, Top-view schematic of the ML-Si_2Te_2 grown on the $HfTe_2$ substrate.

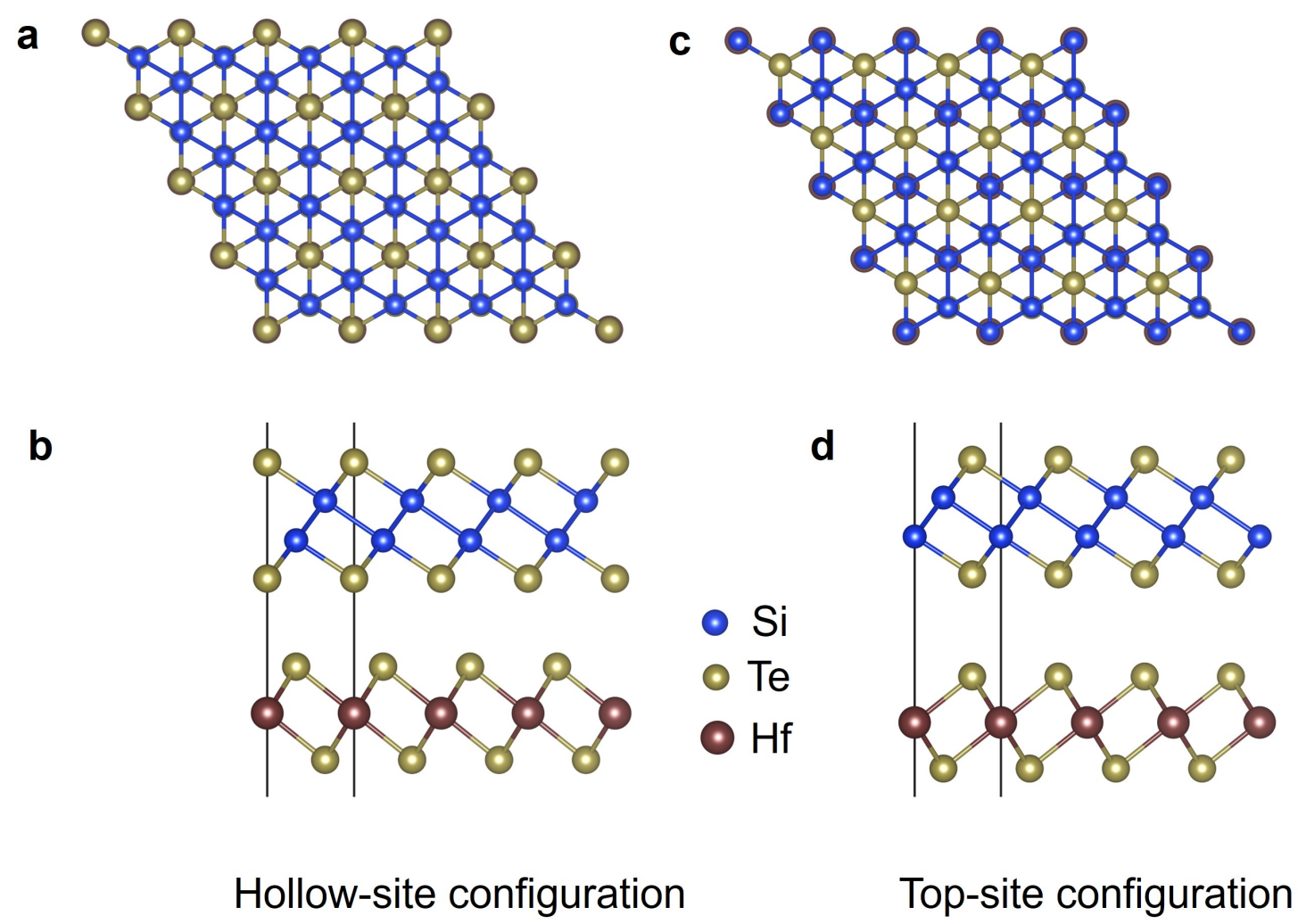

FIG. S11. DFT-calculated crystallographic orientation of ML-Si_2Te_2 islands relative to the $HfTe_2$ substrate. **a**,**b**, Top and side views of the hollow-site configuration. **c**,**d**, Top and side views of the top-site configuration. The vertical lines indicate the unit cells.

dI/dV measurements of the band edges in ML-Si_2Te_2 grown on $HfTe_2$

Determining the band edges of ML-Si_2Te_2 grown on $HfTe_2$ using dI/dV spectroscopy is challenging due to the influence of metallic substrate states on the tunneling process. To mitigate this effect, we employed a controlled tip modification procedure to achieve high-resolution dI/dV measurements (Fig. S12a). By gently tapping the tip on the sample surface, we obtained a well-defined tip apex, as evidenced by the sharp step edges in the STM image (Fig. S12b). Following tip modification, the dI/dV spectrum acquired on the $HfTe_2$ substrate exhibited oscillatory features (indicated by black arrows in Fig. S12d) that were absent in spectra obtained with the unmodified tip (Fig. 4a). These oscillations indicate that the modified tip possesses pronounced features in its local density of states, originating from atomic-scale structural modifications at the apex or localized electronic resonances. Such resonances enhance the energy selectivity of tunneling, thereby enabling the high-resolution dI/dV measurements. The band edges shown in the inset of Fig. 4a were measured using this modified tip at the position marked by the red cross in Fig. S12b. Figure S12c presents a linear plot of the dI/dV spectrum shown in the inset of Fig.4a. To verify the robustness of the extracted band gaps, we performed additional dI/dV measurements at varying tip-sample distances. As shown in Fig. S12e-i, the band gap feature remains unchanged, confirming

that the measured gap is intrinsic rather than an artifact of tip-induced band bending.

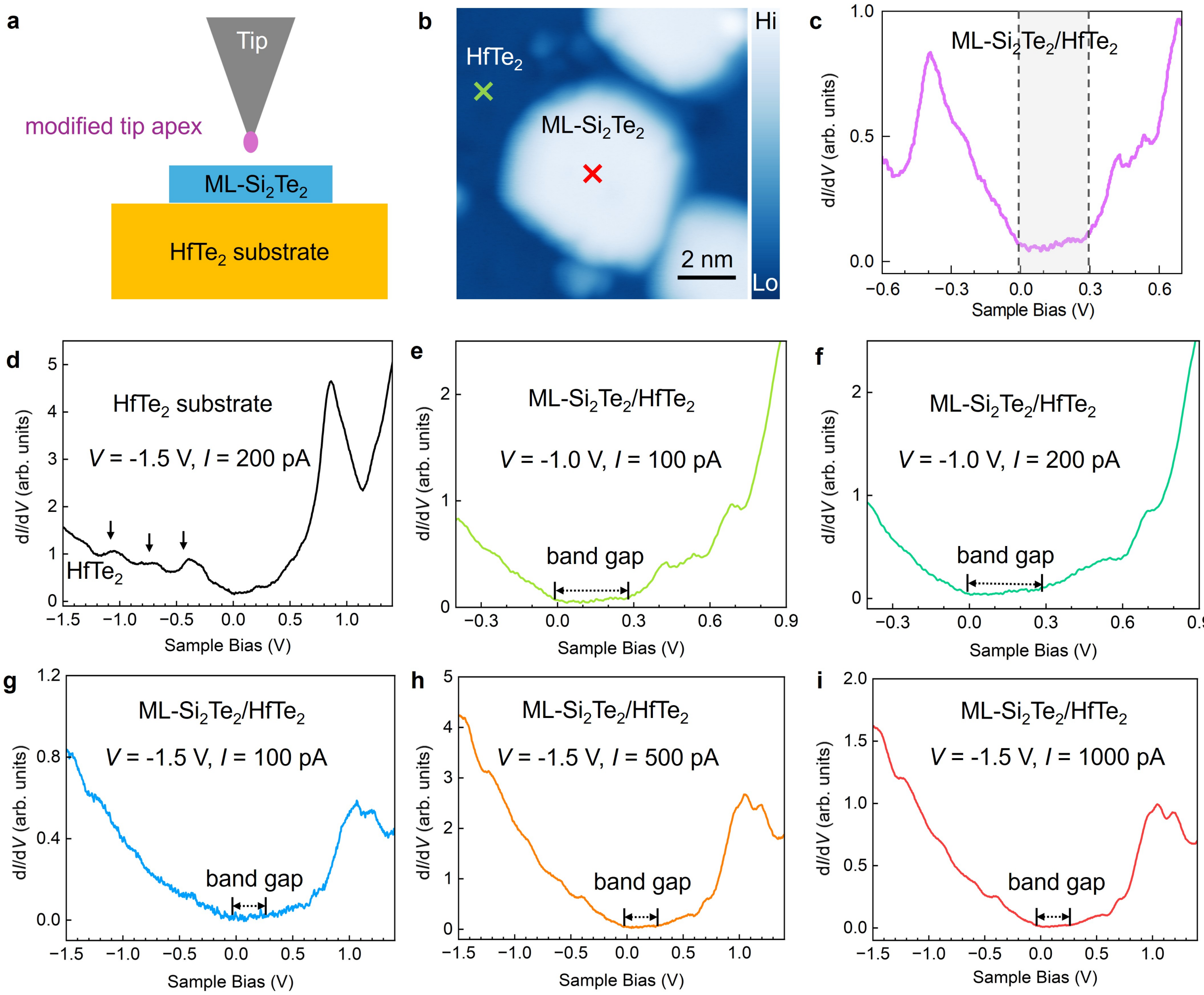

FIG. S12. High-resolution $\mathrm{d}I/\mathrm{d}V$ measurement. **a**, Schematic of the high-resolution $\mathrm{d}I/\mathrm{d}V$ measurements. **b**, STM image ($V = 1.0\,\mathrm{V}$, $I = 10\,\mathrm{pA}$) of ML-Si_2Te_2 islands acquired with a modified tip. **c**, Linear plot of the $\mathrm{d}I/\mathrm{d}V$ spectrum in the inset of Fig.4a. **d**, $\mathrm{d}I/\mathrm{d}V$ spectrum ($V_{\mathrm{stab}} = -1.5\,\mathrm{V}, I_{\mathrm{stab}} = 200\,\mathrm{pA}$) measured on the $HfTe_2$ substrate at the location indicated in (**b**). The black arrows indicate three peak features in the spectrum measured on $HfTe_2$. **e-i**, $\mathrm{d}I/\mathrm{d}V$ spectra acquired on ML-Si_2Te_2 at the same measurement location indicated in (**b**). The horizontal double-headed arrows indicate the conductance onsets used to estimate the band gap. Measurement conditions: (**e**) $V_{\mathrm{stab}} = -1.0\,\mathrm{V}, I_{\mathrm{stab}} = 100\,\mathrm{pA}$; (**f**) $V_{\mathrm{stab}} = -1.0\,\mathrm{V}, I_{\mathrm{stab}} = 200\,\mathrm{pA}$; (**g**) $V_{\mathrm{stab}} = -1.5\,\mathrm{V}, I_{\mathrm{stab}} = 100\,\mathrm{pA}$; (**h**) $V_{\mathrm{stab}} = -1.5\,\mathrm{V}, I_{\mathrm{stab}} = 500\,\mathrm{pA}$; (**i**) $V_{\mathrm{stab}} = -1.5\,\mathrm{V}, I_{\mathrm{stab}} = 1000\,\mathrm{pA}$. All spectra were measured with $V_{\mathrm{mod}} = 10\,\mathrm{mV}$.

Band gap measurements on 20 ML-Si_2Te_2 islands

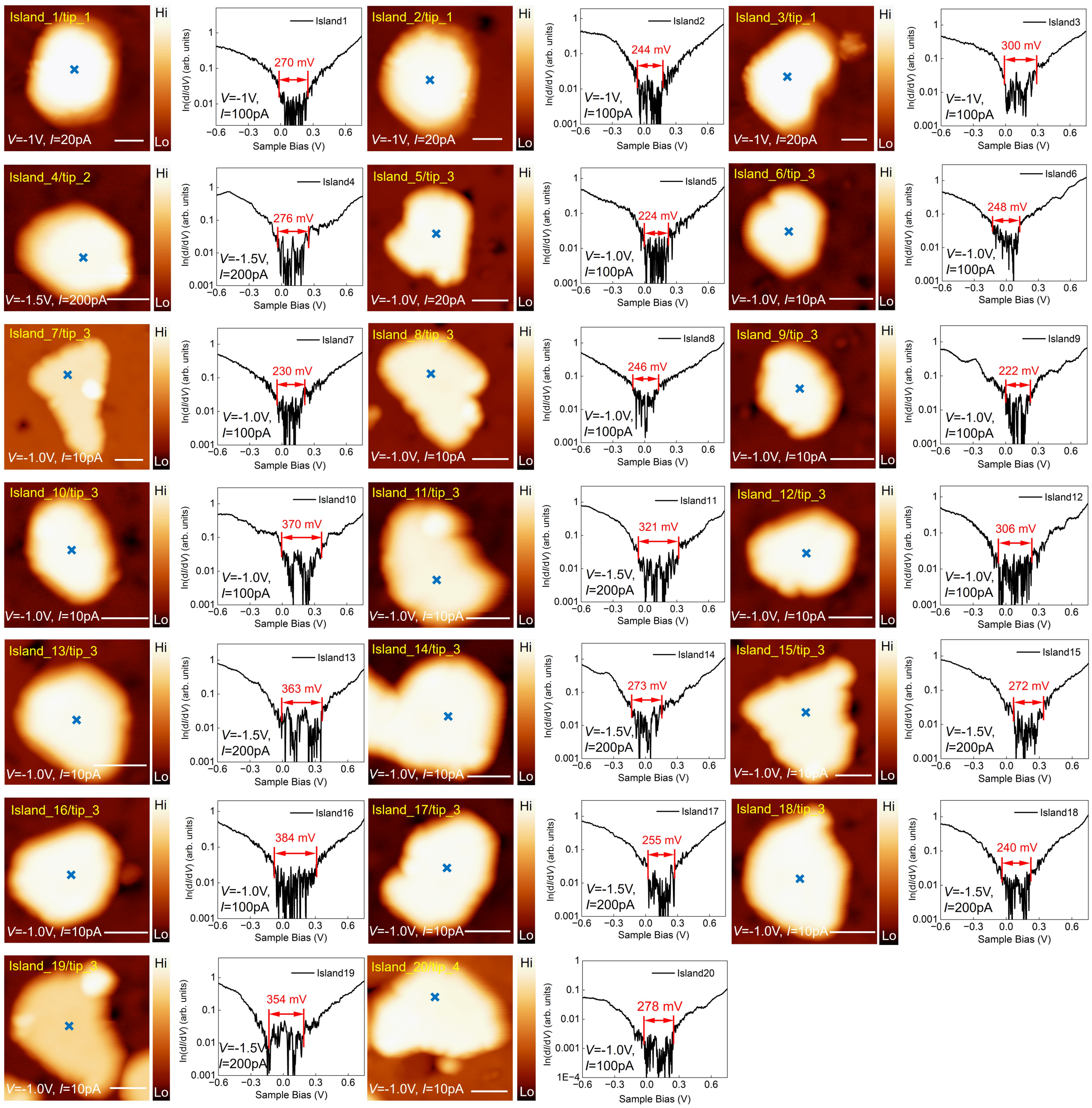

FIG. S13. dI/dV spectra acquired on 20 ML-Si_2Te_2 islands grown on $HfTe_2$ substrates using four different STM tips. Measurement positions are indicated by blue crosses in the corresponding STM images. The horizontal double-headed arrows indicate the conductance onsets used to estimate the band gap. All spectra are plotted on a logarithmic scale. A lock-in voltage modulation (V_{mod}) of 10 mV was used for all measurements.

The relatively small island size of ML-Si_2Te_2 requires careful consideration of possible finite-size effects, including edge-state hybridization and tip-related convolution effects. To evaluate the consistency of the band gap across different experimental conditions, we performed dI/dV spectroscopy on 20 additional ML-Si_2Te_2 islands using four different STM tips (see Fig. S13). The band edges were extracted from the inflection points of the corresponding $\ln(dI/dV)$ spectra. The resulting average band gap, 284 ± 51 meV, agrees well with the estimate from Fig.4a, within the experimental uncertainty, and is also reasonably consistent with the DFT-calculated value of 319 meV shown in Fig.2a. These results strongly support the intrinsic nature of the band gap in ML-Si_2Te_2 and make tip-induced artifacts unlikely.

We note that the determination of the bulk band edges was performed under stabilization conditions with relatively large bias voltages (± 1.0 to ± 1.5 V) and moderate tunneling currents (100–200 pA), corresponding to a comparatively large tip-sample distance. Under these conditions, the tunneling spectra are expected to be dominated by bulk electronic states, whereas the contribution from edge-localized states is expected to be significantly reduced. Therefore, even if residual edge-state contributions remain present owing to the finite lateral dimensions of the islands, the bulk electronic states are expected to dominate the tunneling spectra under the stabilization conditions employed here, enabling a reasonable estimation of the band-edge energies. Moreover, no clear spectroscopic signature that can be unambiguously attributed to finite-size-induced edge-state hybridization is observed in the measured spectra.

In addition, width-dependent DFT calculations of ML-Si_2Te_2 nanoribbons, presented below in Fig. S19, show that edge-state hybridization is strongly suppressed once the ribbon width reaches approximately 4.59 nm. A large fraction of the ML-Si_2Te_2 islands included in the statistical analysis shown in Fig. S13 have lateral dimensions exceeding this value, indicating that finite-size-induced edge-state hybridization is expected to be substantially reduced for most of the islands considered in the statistical analysis and is therefore unlikely to significantly affect the extracted band-gap values.

Edge states measurements on different ML-Si_2Te_2 islands

Edge-state measurements were performed on several ML-Si_2Te_2 islands. As shown in Fig. 5, Fig. S14, Fig. S15, and Fig. S16, the characteristic peak-like edge-state feature at the

island edges and the dip-like feature near the island center are consistently observed on different islands. We note that the ML-Si_2Te_2 islands investigated in this work are grown on a metallic $HfTe_2$ substrate. As a result, the $\mathrm{d}I/\mathrm{d}V$ spectra measured near the island center reflect the electronic structure of ML-Si_2Te_2 superimposed on a finite background contribution from the substrate. We therefore interpret the dip-like feature as arising from the finite background contribution of the metallic $HfTe_2$ substrate within the bulk-gap energy window of ML-Si_2Te_2. For the same reason, the tunneling conductance remains finite within the gap region and does not exhibit a fully vanishing hard-gap-like behavior.

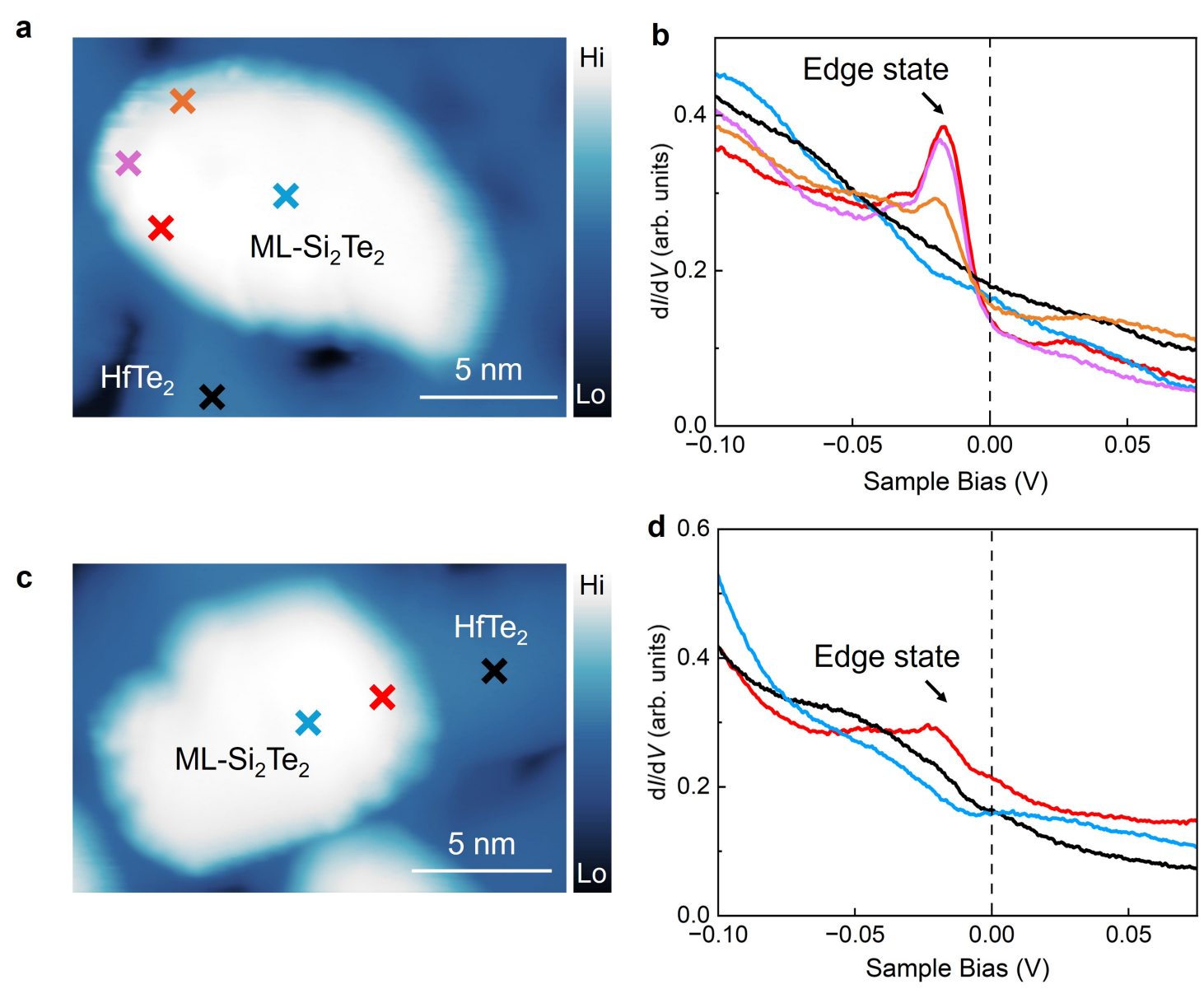

FIG. S14. Edge states measured on two ML-Si_2Te_2 islands. **a**,**c**, STM images ($V = -1.0\,\mathrm{V}$, $I = 10\,\mathrm{pA}$) of ML-Si_2Te_2 islands. **b**,**d**, $\mathrm{d}I/\mathrm{d}V$ spectra ($V_{\mathrm{stab}} = -0.10\,\mathrm{V}, I_{\mathrm{stab}} = 100\,\mathrm{pA}$, and $V_{\mathrm{mod}} = 5\,\mathrm{mV}$) acquired at the step edges and centers of the ML-Si_2Te_2 islands, as well as on the $HfTe_2$ substrate, at the positions indicated in (**a**) and (**c**). The black dashed lines mark the E_{F}.

Discussion of the peak-like edge states observed in Fig. 5b,c

In our $\mathrm{d}I/\mathrm{d}V$ measurements, the edge-state signal observed at the boundaries of ML-Si_2Te_2 islands appears as a pronounced peak-like feature. Similar spectral signatures have been frequently reported in STM/STS studies of 2D topological insulators and attributed to 1D topological edge states [S8–S10]. Although the microscopic origin may vary among differ-

ent systems, peak-like edge-state features appear to be a common characteristic of tunneling measurements on topological edge channels. Below, we discuss two possible mechanisms that may contribute to the peak-like feature observed in ML-Si_2Te_2.

In ML-Si_2Te_2, the Dirac point of the edge states is located at the Γ point (Fig. 2b), at the center of the Brillouin zone. Since STM tunneling generally favors states with small parallel momentum, states near the Γ point may have a relatively large tunneling weight in STM measurements. As the energy moves away from the Dirac point, the edge-state momentum increases, which can reduce the tunneling matrix element and naturally lead to a peak-like enhancement of the $\mathrm{d}I/\mathrm{d}V$ signal near the Dirac-point energy. Based on the linear part of the edge-state dispersion in Fig. 2b, we estimated the Fermi velocity to be $v_{\mathrm{F}} = \frac{1}{\hbar}\frac{dE}{dk} = 5.47 \times 10^5\ \mathrm{m\,s^{-1}}$, which is a typical value for topological edge states in QSH systems (10^5–$10^6\ \mathrm{m\,s^{-1}}$). This value suggests that the parallel momentum of the edge state increases appreciably as the energy moves away from the Dirac point, indicating that the momentum-dependent tunneling scenario is physically reasonable.

A second possible explanation is related to the intrinsic DOS of the edge-state bands. As shown in Fig. 5f, the calculated edge-state band structure of ML-Si_2Te_2 exhibits a Dirac-cone-like feature at the Γ point, characteristic of its topological nature. The edge states display approximately linear dispersion close to the Dirac point, resulting in a nearly constant DOS. In the present system, however, the measured $\mathrm{d}I/\mathrm{d}V$ spectra contain a finite background contribution from the metallic $HfTe_2$ substrate, which can partially obscured this weak and nearly energy-independent DOS signal. Away from the Dirac point, the edge-state bands become nonlinear and exhibit finite band curvature. Such nonlinear dispersion can give rise to an enhanced DOS and van-Hove-singularity-like spectral feature. Consequently, the experimentally observed edge-state signal is expected to be dominated by these enhanced DOS features rather than by the nearly constant DOS associated with the linear part of the edge-state dispersion. Since the nonlinear segment spans only a limited energy range, the resulting spectral feature appears within a relatively narrow energy window compared with the full gap. Furthermore, the detailed edge-state dispersion and interactions with the substrate can shift the energy position of the enhanced DOS feature away from the middle of the bulk gap. Therefore, the experimentally observed edge-state peak does not necessarily coincide with the mid-gap energy.

Real-space dI/dV mapping of the edge states

Before performing the real-space dI/dV mapping of the edge states on the representative ML-Si_2Te_2 island shown in Fig.5d,e, we first determined the energy position of the edge-state feature from point dI/dV spectra. As shown in Fig. S15, the spectrum acquired at the island center exhibits a V-shaped dip near E_F, whereas the spectrum measured near the step edge displays a pronounced peak at approximately +7 mV. To examine the reproducibility of this feature, dI/dV spectra were acquired at multiple edge segments of the same island (see Fig. S16). The edge-state peak near +7 mV is consistently observed at different edge locations, although its intensity and exact energy position show spatial variation. Such variations are likely associated with differences in the local edge geometry, atomic-scale structure, and chemical environment. Based on the energy positions identified from the spectra shown in Fig. S15 and Fig. S16, the dI/dV maps presented in Fig.5e and Fig. S17 were acquired to visualize the spatial distribution of the edge states.

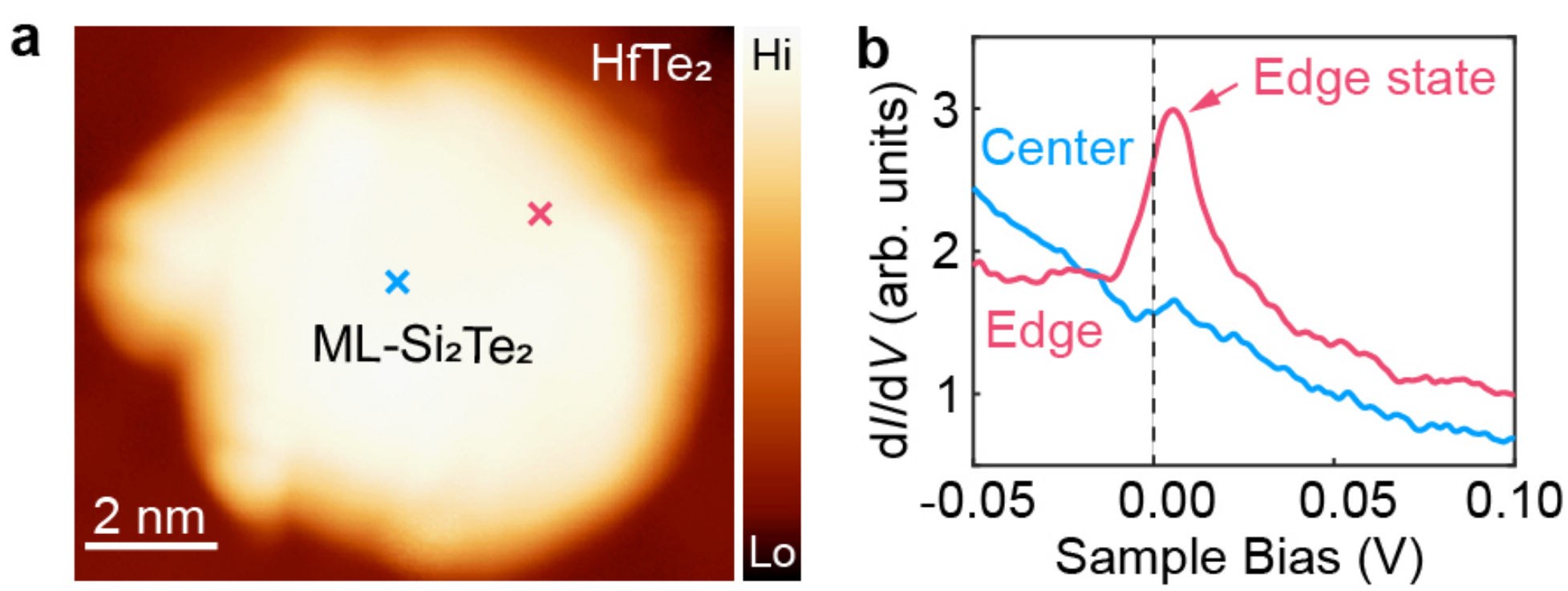

FIG. S15. **a**, STM image ($V = 1.0$ V, $I = 10$ pA) of a ML-Si_2Te_2 island. **b**, dI/dV spectra ($V_{stab} = -0.05$ V, $I_{stab} = 100$ pA, and $V_{mod} = 1$ mV) measured at the edge and center of the island at the positions indicated in (**a**).

Theoretically, topological edge states are protected by time-reversal symmetry and are expected to be robust against nonmagnetic disorder and geometric imperfections. Experimentally, however, limitations such as tip asymmetry and irregular edge morphology often make real-space visualization of continuous edge channels challenging [S8, S9]. In this context, the boundary-localized signal observed at multiple locations along the island edge in Fig.5e and Fig. S17, is consistent with the expected robustness of topological edge states, despite local variations in signal intensity.

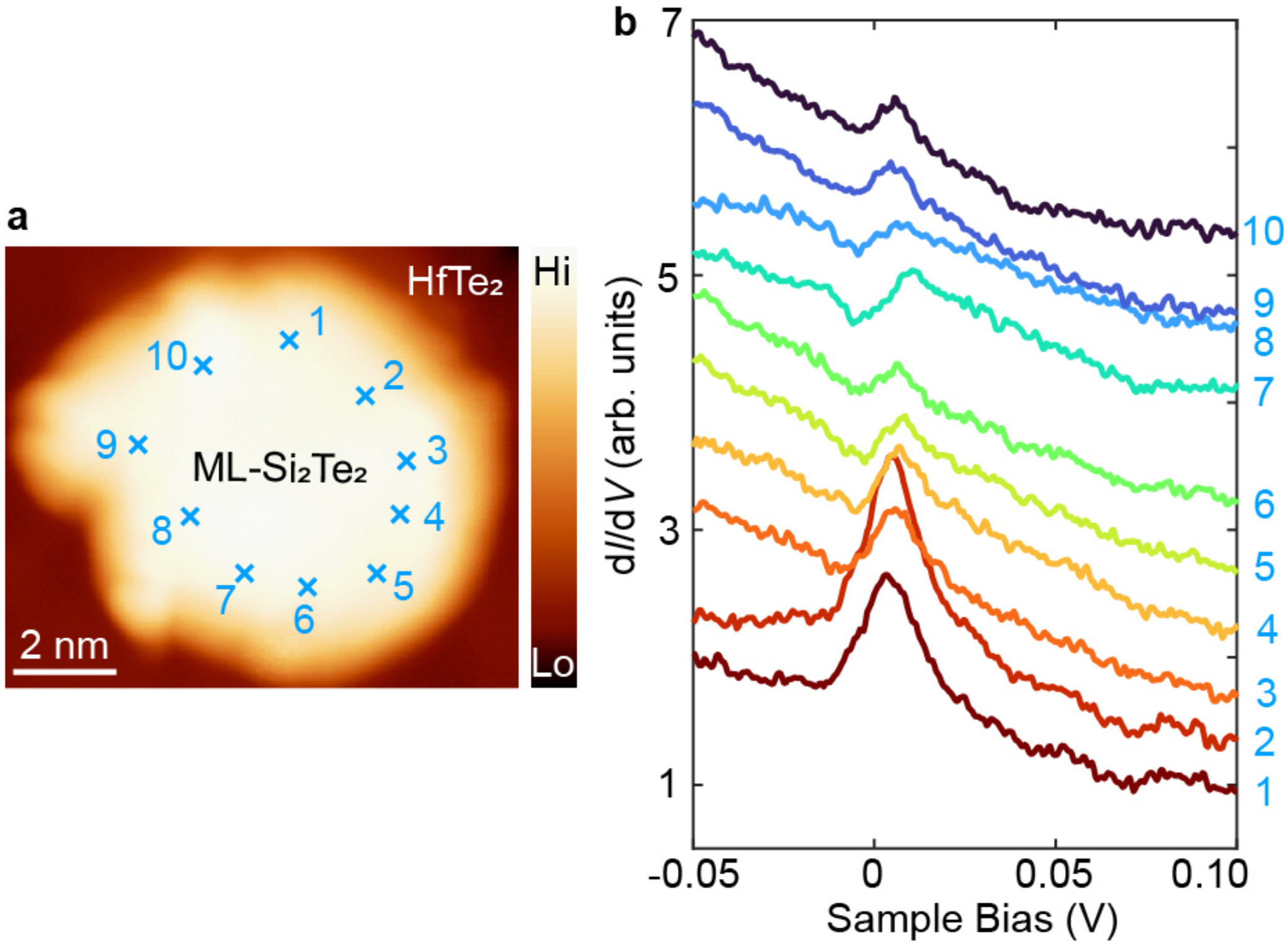

FIG. S16. Edge states measured at different edges of the ML-Si_2Te_2 island shown in Fig. 5d of the main manuscript. **a**, STM image ($V = 1.0\,\mathrm{V}$, $I = 10\,\mathrm{pA}$) of the ML-Si_2Te_2 island. **b**, $\mathrm{d}I/\mathrm{d}V$ spectra ($V_{\mathrm{stab}} = -0.05\,\mathrm{V}, I_{\mathrm{stab}} = 100\,\mathrm{pA}$, and $V_{\mathrm{mod}} = 1\,\mathrm{mV}$) acquired at different edges of the island, with the measurement positions indicated in (**a**).

We further note that the data shown in Fig.5d,e and Fig. S15 - Fig. S17 were acquired using a newly installed STM head and a different tip than those used for the measurements shown in Fig. 5a-c. The $\mathrm{d}I/\mathrm{d}V$ spectra in Fig. S15b reproduce the key spectral features observed in Fig. 5b, including the V-shaped dip and the edge-state peak. This consistency across different STM configurations and tips further supports the reproducibility of the observed edge-state signatures in ML-Si_2Te_2.

A useful comparison can be made with our previous study of ML-Si_2Te_2 grown on Sb_2Te_3 substrate [S4]. In that system, the large interfacial strain drives ML-Si_2Te_2 into a topologically trivial semiconducting phase, and no in-gap edge states were observed at the ML-Si_2Te_2/Sb_2Te_3 boundaries. In contrast, the present work realizes strain-free ML-Si_2Te_2 on $HfTe_2$ together with pronounced in-gap edge states. These comparative results provide an important control experiment supporting the interpretation of the observed edge states as

arising from the predicted topologically nontrivial phase in ML-Si_2Te_2 grown on $HfTe_2$.

Additional dI/dV maps acquired at different energies are shown in Fig. S17. At +5 mV (Fig. S17a), a pronounced boundary-localized feature is observed, consistent with the edge-state signal discussed in Fig. 5e in the main text. At +50 mV (Fig. S17b), the boundary feature remains discernible but becomes increasingly obscured by the growing contribution of metallic states from the $HfTe_2$ substrate. Figures S17c,d show dI/dV maps acquired at +5.5 and +8.5 mV, respectively, with improved spatial resolution. Both maps reproduce the boundary-localized edge-state nature and further support the spatial localization of the edge states along the island boundary.

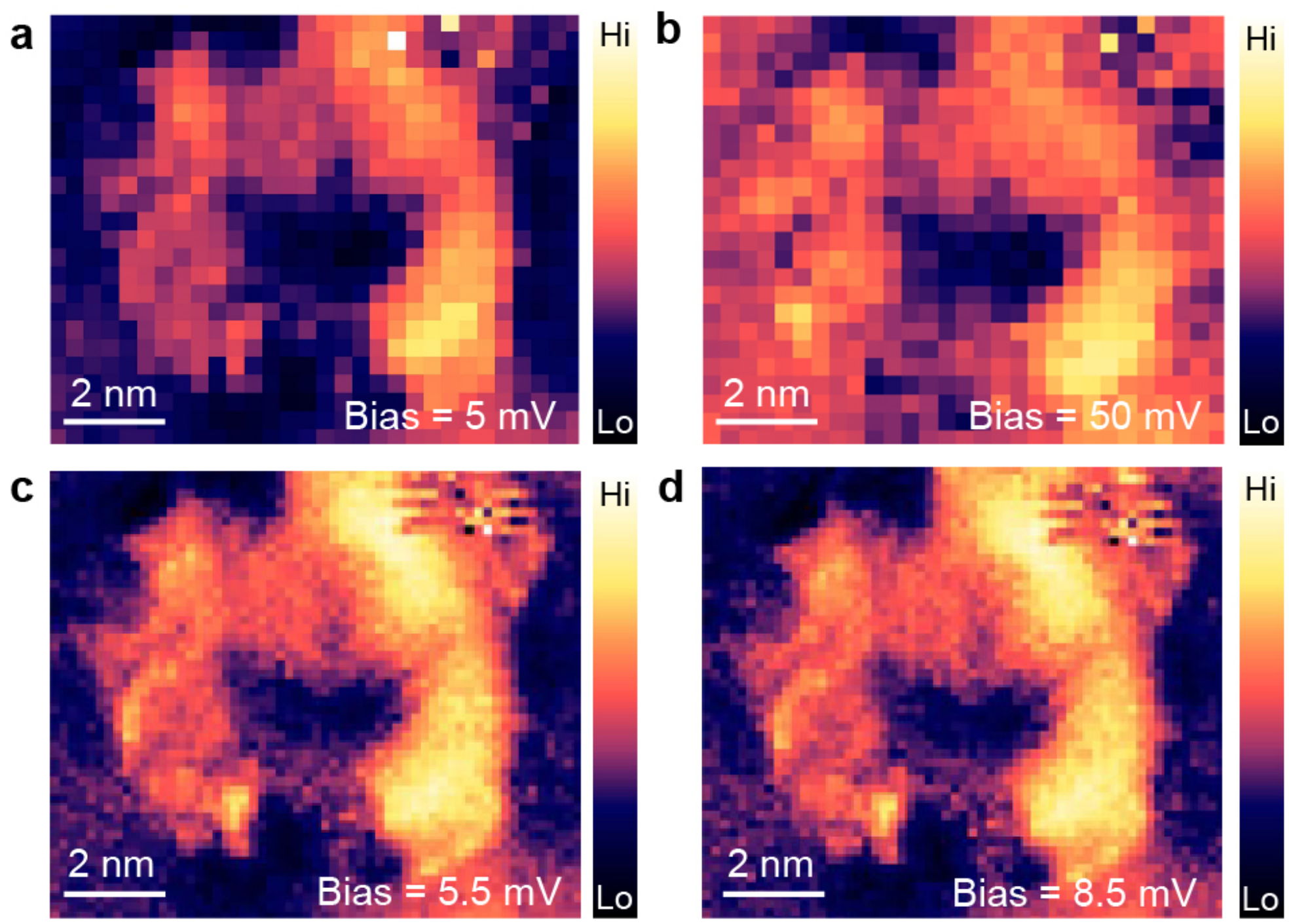

FIG. S17. dI/dV maps ($V_{stab} = 0.2$ V, $I_{stab} = 100$ pA, and $V_{mod} = 2.0$ mV) acquired at +5 mV (**a**), +50 mV (**b**), +5.5 mV (**c**), and +8.5 mV (**d**), for the area shown in Fig. 5d. Panels (**c**) and (**d**) were acquired with twice the pixel density.

Calculated electronic structures of ML-Si_2Te_2 nanoribbons with different widths

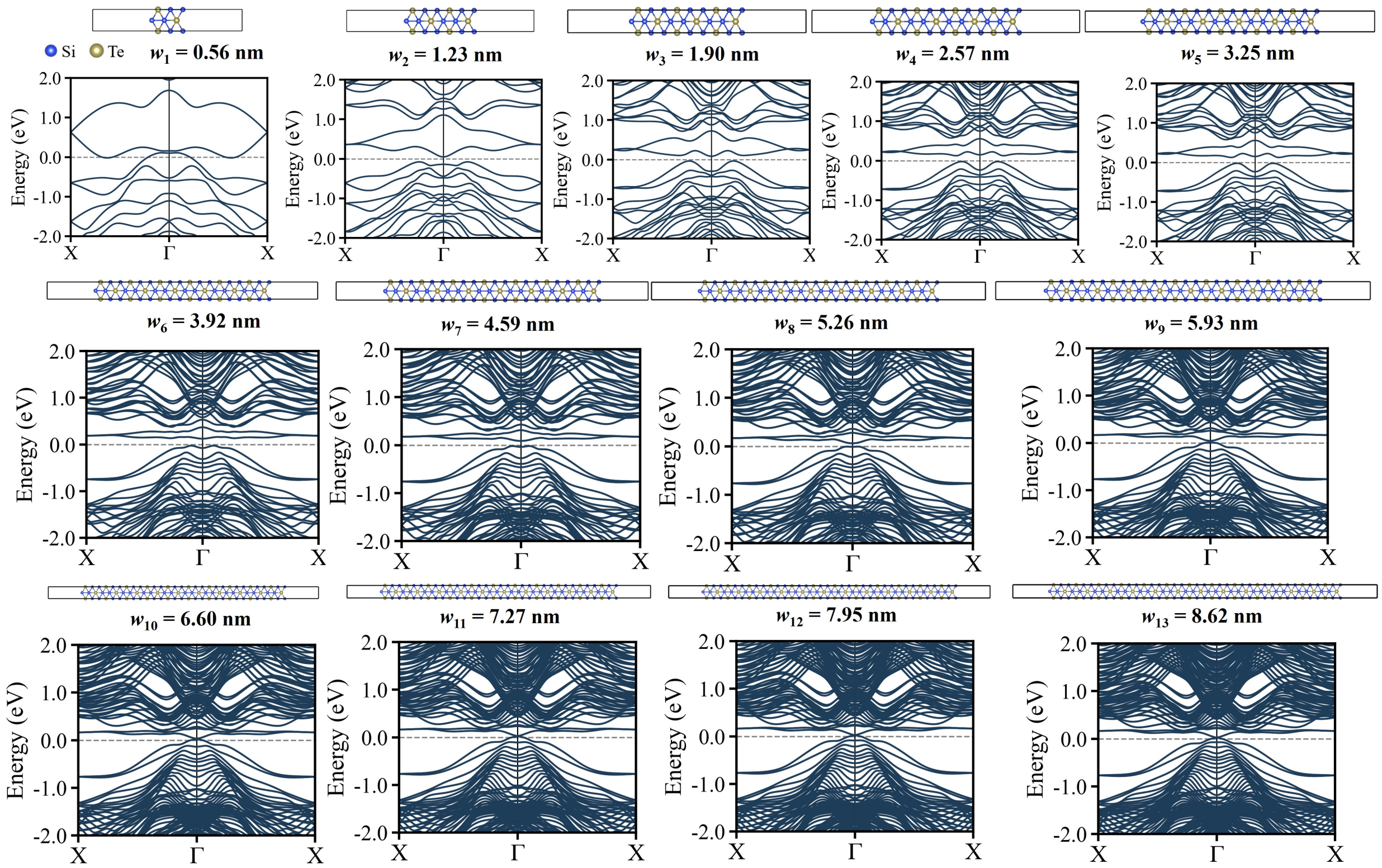

FIG. S18. DFT-calculated electronic band structures of free-standing ML-Si_2Te_2 nanoribbons with ribbon widths (w) ranging from 0.56 to 8.62 nm. Schematic structural models of each nanoribbon are shown together with the corresponding calculated electronic band structures. SOC is included in all calculations. Vacuum spacings of 20 Å were introduced along the lateral direction to suppress spurious interactions between periodically repeated nanoribbon edges. In addition, vacuum spacings of 30 Å were introduced along the out-of-plane (z) direction to eliminate spurious interactions between periodically repeated ML-Si_2Te_2 slabs. For w_n, n denotes the number of unit cells along the ribbon-width direction. The horizontal dashed lines indicate the E_F.

We systematically investigated the electronic structures of ML-Si_2Te_2 nanoribbons with different widths from 0.56 nm to 8.62 nm (Fig. S18). The calculations show that the electronic structures of relatively narrow nanoribbons are strongly influenced by finite-size effects. In ribbons with insufficient width, residual inter-edge hybridization perturbs the edge-state dispersion, such that the energetic separation between edge-derived and bulk-derived states near E_F is not yet fully developed. As the ribbon width increases, the inter-edge coupling is

progressively suppressed due to the increased separation between the two edges. For ribbon widths of 6.60 nm and larger, the edge states dispersion develops a clear Dirac-like crossing at the Γ point. At the same time, the bulk-derived bands become energetically separated from E_F, resulting in a local energy window around $E = 0$ eV in which the electronic states are dominated by the topological edge channels. These results indicate that, in sufficiently wide nanoribbons, the topological edge states remain energetically separated from bulk-derived states near E_F, which is favorable for edge-state transport. These results are consistent with the real-space charge-density analysis presented above in Figure S19 and Fig. 5f. Although boundary-localized edge states with negligible inter-edge overlap already emerge for ribbon widths of 4.59 nm (Figure S19), a fully developed Dirac-like crossing requires larger widths to completely suppress the remaining finite-size effects in the band dispersion.

Additional real-space charge-density distributions corresponding to Fig. 5f

Figure S19 presents the calculated real-space charge density of the topological edge states in ML-Si_2Te_2 nanoribbons with ribbon widths of $w = 3.92$, 4.59, 5.26, 5.93 and 8.62 nm. The $w = 4.59$ nm case is included here as a reference. For $w = 4.59$ nm, the edge-state wavefunctions are already strongly localized at the ribbon boundaries and are mainly confined within ≈ 2.0 nm inward from the boundary, with negligible amplitude at the ribbon center. Furthermore, the inferred spatial extent of ≈ 2.0 nm remains essentially unchanged for nanoribbon widths between 4.59 and 8.62 nm, indicating that the edge-state localization is already well converged in this width regime.

It is worth noting that the observed spatial extent of ≈ 2.0 nm is consistent with previous experimental and theoretical studies of topological edge states in various QSH systems, whose penetration depth is known to depend sensitively on the underlying electronic structure and can vary substantially among different material systems [S10–S12].

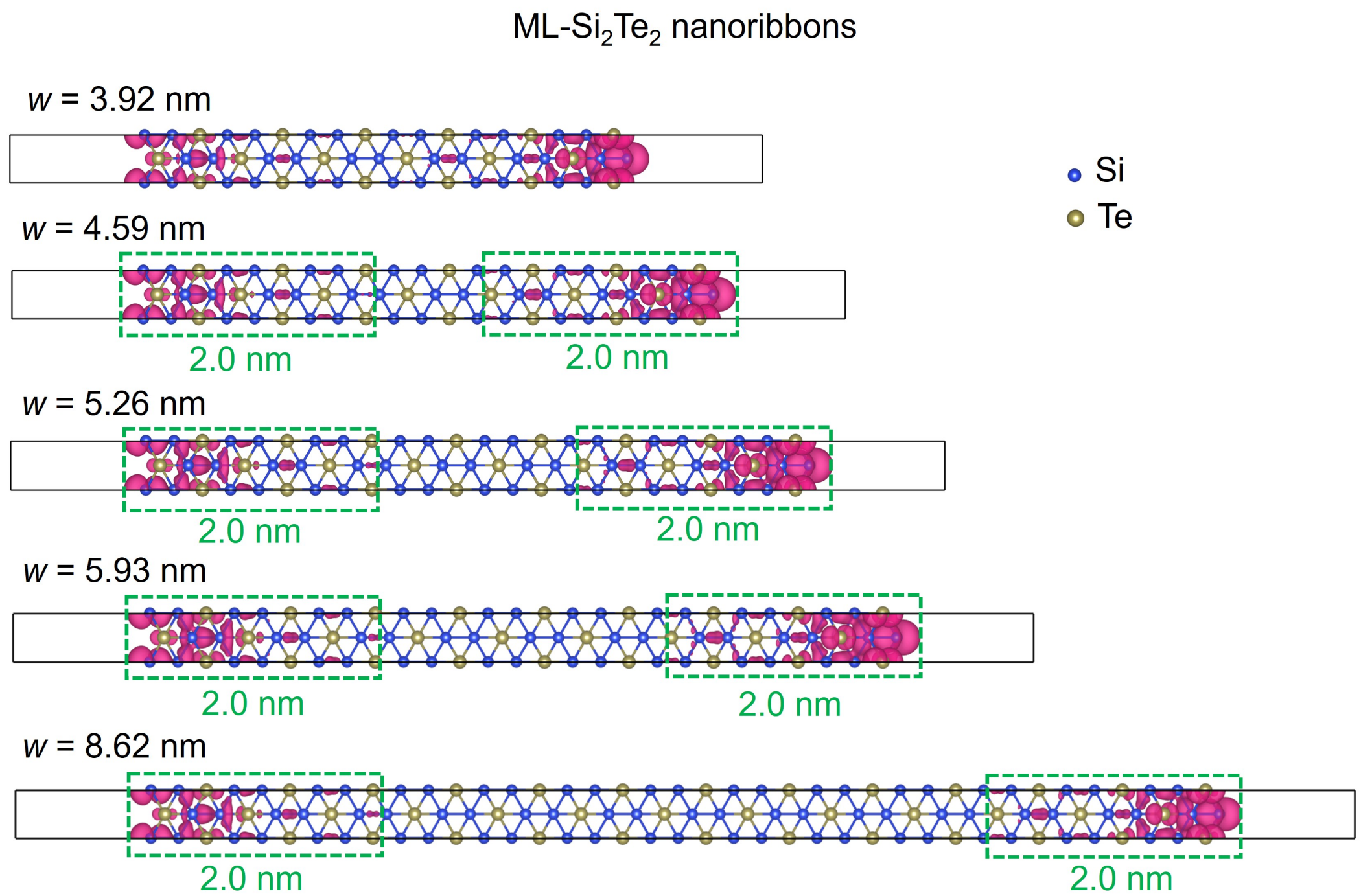

FIG. S19. Real-space charge-density distributions (shown as magenta isosurfaces) of the topological edge states in free-standing ML-Si_2Te_2 nanoribbons with ribbon widths of w = 3.92, 4.59, 5.26, 5.93 and 8.62 nm. The charge density was calculated within an energy window of ±50 meV around E_F. The isosurface value was set to 0.0001 $e/Å^3$. SOC is included in all calculations. Vacuum spacings of 20 Å were introduced along the lateral direction to suppress spurious interactions between periodically repeated nanoribbon edges. In addition, vacuum spacings of 30 Å were introduced along the out-of-plane (z) direction to eliminate spurious interactions between periodically repeated ML-Si_2Te_2 layers. The edge-state distributions are highlighted by the green dashed boxes.

Phonon-mediated scattering in ML-Si_2Te_2

Phonon-mediated scattering can play an important role in determining finite-temperature transport properties in telluride-based systems. Electron-phonon coupling (EPC) calculations were performed within the framework of density functional perturbation theory (DFPT), as implemented in the Quantum ESPRESSO package [S13, S14]. Optimized norm-conserving Vanderbilt (ONCV) pseudopotentials were employed [S15]. The plane-wave kinetic-energy cutoff and charge-density cutoff were set to 70 Ry and 300 Ry, respectively. Dynamical matrices and EPC quantities were calculated on a uniform $6 \times 6 \times 1$ **q**-point grid.

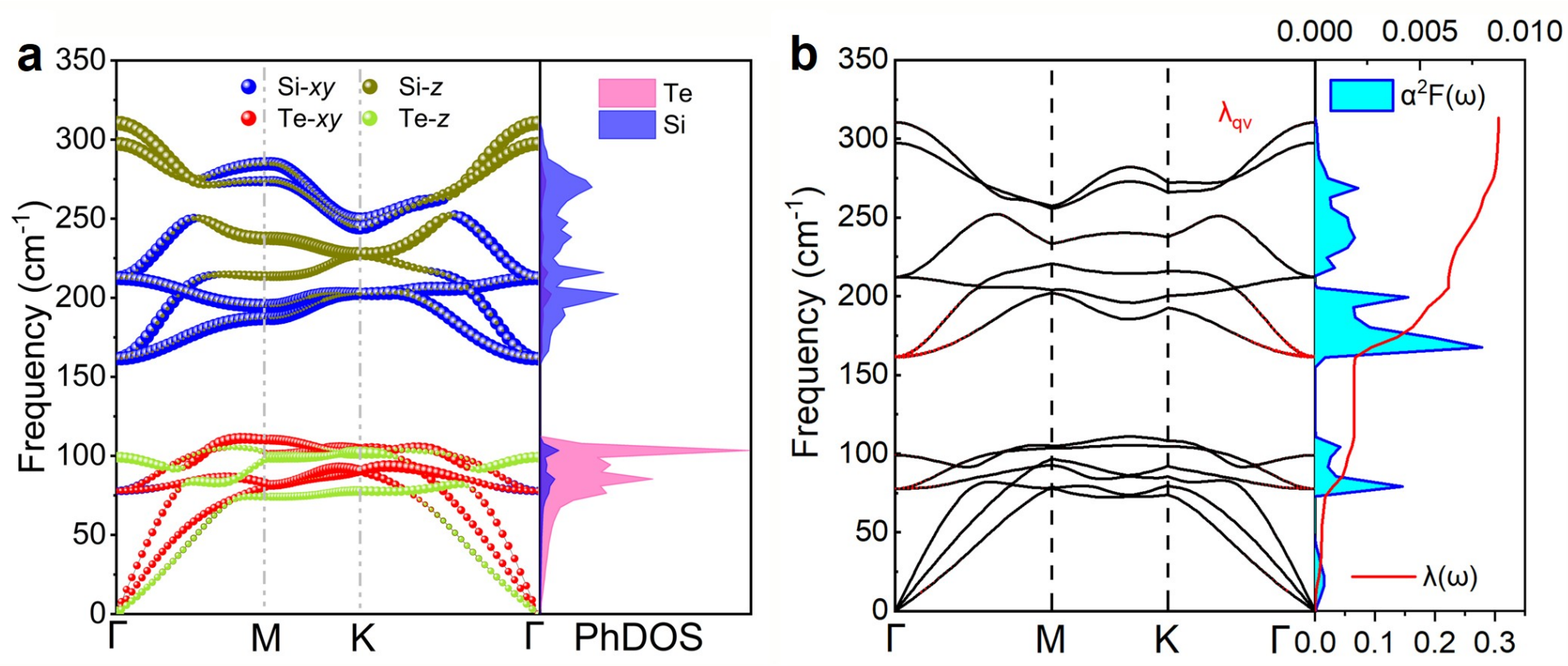

FIG. S20. Phonon spectra and EPC properties of free-standing ML-Si_2Te_2. **a**, Calculated phonon dispersion (left panel) and projected phonon density of states (PhDOS) (right panel) of ML-Si_2Te_2. The phonon branches are weighted by the atomic-projected vibrational amplitudes of Si and Te atoms. Blue and red circles correspond to the in-plane vibrational modes of Si and Te atoms (Si-xy and Te-xy), respectively, while olive and light-green circles denote the corresponding out-of-plane vibrational modes (Si-z and Te-z). **b**, Calculated EPC properties of ML-Si_2Te_2, including the mode-resolved EPC strength $\lambda_{\mathbf{q}\nu}$, Eliashberg spectral function $\alpha^2F(\omega)$, and cumulative EPC constant $\lambda(\omega)$. The blue shaded area represents $\alpha^2F(\omega)$, while the red curve denotes $\lambda(\omega)$.

The calculated phonon spectra and EPC properties are shown in Fig. S20. The PhDOS shows that the low-frequency modes are mainly associated with Te vibrations, whereas the higher-frequency modes are primarily related to in-plane Si vibrations. The mode-resolved EPC constant $\lambda_{\mathbf{q}\nu}$, Eliashberg spectral function $\alpha^2F(\omega)$, and the cumulative EPC constant $\lambda(\omega)$ [S16] are also presented in Fig. S20. The total EPC constant is $\lambda \approx 0.3$, indicating relatively weak-to-moderate EPC [S17, S18]. Importantly, the dominant EPC contributions originate from optical phonon modes in the range of 140–200 cm^{-1}, whereas the low-frequency acoustic modes, which are typically most relevant for charge-carrier scattering and transport, contribute comparatively weakly. These results suggest that phonon-mediated scattering in ML-Si_2Te_2 is expected to remain relatively weak.

***Corresponding authors**: Xiaochun Huang: xiaochun.huang@uni-wuerzburg.de,
Rui Xiong: 398523@fzu.edu.cn, Baisheng Sa: bssa@fzu.edu.cn
†**These authors contributed equally**: Xiaochun Huang, Lingxiao Zhao

[S1] Björkman, T., Gulans, A., Krasheninnikov, A. V. & Nieminen, R. M. van der Waals bonding in layered compounds from advanced density-functional first-principles calculations. *Phys. Rev. Lett.* **108**, 235502 (2012).

[S2] Sa, B., Sun, Z. & Wu, B. The development of two dimensional group iv chalcogenides, blocks for van der Waals heterostructures. *Nanoscale* **8**, 1169–1178 (2016).

[S3] Ma, Y., Kou, L., Dai, Y. & Heine, T. Proposed two-dimensional topological insulator in SiTe. *Phys. Rev. B* **94**, 201104 (2016).

[S4] Huang, X. *et al.* Experimental realization of semiconducting monolayer Si_2Te_2 films. *Adv. Funct. Mater.* **32**, 2208281 (2022).

[S5] Mostofi, A. A. *et al.* An updated version of wannier90: A tool for obtaining maximally-localised Wannier functions. *Comput. Phys. Commun.* **185**, 2309–2310 (2014).

[S6] Giustino, F. & Pasquarello, A. Mixed Wannier-Bloch functions for electrons and phonons in periodic systems. *Phys. Rev. Lett.* **96**, 216403 (2006).

[S7] Wang, X. *et al.* Identification of native defects of 1T-$HfTe_2$. *Appl. Phys. Lett.* **122**, 252101 (2023).

[S8] Drozdov, I. K. *et al.* One-dimensional topological edge states of bismuth bilayers. *Nat. Phys.* **10**, 664–669 (2014).

[S9] Pauly, C. *et al.* Subnanometre-wide electron channels protected by topology. *Nat. Phys.* **11**, 338–343 (2015).

[S10] Yang, F. *et al.* Spatial and energy distribution of topological edge states in single bi(111) bilayer. *Phys. Rev. Lett.* **109**, 016801 (2012).

[S11] Wada, M., Murakami, S., Freimuth, F. & Bihlmayer, G. Localized edge states in two-dimensional topological insulators: Ultrathin bi films. *Phys. Rev. B* **83**, 121310 (2011).

[S12] Lai, K. *et al.* Imaging of coulomb-driven quantum hall edge states. *Phys. Rev. Lett.* **107**, 176809 (2011).

[S13] Giannozzi, P. *et al.* Quantum espresso: a modular and open-source software project for quantum simulations of materials. *J. Phys. Condens. Matter* **21**, 395502 (2009).

[S14] Giannozzi, P. *et al.* Advanced capabilities for materials modelling with quantum espresso. *J. Phys. Condens. Matter* **29**, 465901 (2017).

[S15] Schlipf, M. & Gygi, F. Optimization algorithm for the generation of oncv pseudopotentials. *Comput. Phys. Commun.* **196**, 36–44 (2015).

[S16] Eliashberg, G. M. Interactions between electrons and lattice vibrations in a superconductor. *Sov. Phys. JETP* **11**, 696–702 (1960).

[S17] Penev, E. S., Kutana, A. & Yakobson, B. I. Can two-dimensional boron superconduct? *Nano Lett.* **16**, 2522–2526 (2016).

[S18] Shi, L.-T. *et al.* High-Throughput Screening $MgFe_6Ge_6$-Prototype Bilayer Kagome Family. *Chin. Phys. Lett.* **43**, 030711–030711 (2026).
