# Supplementary material for: Experimental realization of the topologically nontrivial phase in monolayer Si2Te2
Source: Nat Commun. 2026 Sep 11;17:9741. doi: 10.1038/s41467-026-77669-9 (PMC13569488; doi:10.1038/s41467-026-77669-9)
Supplement: Supplementary file 2 [file 41467_2026_77669_MOESM2_ESM.pdf]

nature portfolio

Peer Review File

Experimental realization of the topologically nontrivial phase in monolayer Si_2Te_2

Corresponding Author: Dr Xiaochun Huang

Parts of this Peer Review File have been redacted as indicated to remove third-party material.

This file contains all reviewer reports in order by version, followed by all author rebuttals in order by version.

Version 0:

Reviewer comments:

Reviewer #1

(Remarks to the Author)
This manuscript reports MBE growth of monolayer Si2Te2 on cleaved HfTe2 and the authors argue that the film is strain-free (giving a 1x1 structure), with a bandgap of about 300 meV and edge localized in gap states. Based on these (and theory) the authors declare Si2Te2 a QSH insulator. I think this work is promising and timely, and there is a good combination of STM/STS and DFT. However, the current paper, particularly the QSH claim, requires additional analysis, control experiments, and more internally consistent evidence.
1. The claim of topology rests primarily on the observation of in-gap states at the island boundaries. While I agree that this is consistent with a QSH edge-state picture, edge-enhanced LDOS alone is not unique to QSH systems. Trivial edge resonances, band bending, or reconstruction-induced states can mimic similar phenomenology. The authors should provide additional evidence or a control experiment (beyond edge vs bulk contrast) to justify the claim on the experimental realization of the QSH phase in Si2Te2.
2. The islands are very small, the reported edge-state spatial extent (\sim2-3 nm) takes a substantial fraction of the island lateral size, coupled with tip-convolution this would obstruct of measuring the true bandgap of the flakes. Additionally, edge states might hybridize with each other which could open a bandgap in the edge state spectrum. Ideally, statistics on larger islands (or a systematic scaling analysis) should be included.
3. Related to that there is another inconsistency. The penetration depth of the edges states is typically inversely proportional to the gap size. The authors have a rather large bandgap, which I would anticipate to give rise to very small spatial extend of the edge states, probably much smaller than the 2-3 nm measured here. Can the authors comment?
4. I do not agree with the statement by the authors that the edge state runs uninterrupted along the whole contour of the flake in Figure 5, there is an obvious and big interruption at the bottom of the flake. Can the authors provide additional evidence/more dIdV maps?
5. Another concern that I have is that different terminations/reconstructions could alter the dispersion of the edge states. Given the islands appear to have rough edges, the STS signature should vary along different edges/segments or with this roughness. Can the authors discuss this further?
6. Typically, for topological systems, the edge-bulk correspondence tells that the boundary state is midgap, however the reported edge states are significantly offset from the midgap.
7. The gap assignment from Fig. 4 is not fully convincing and may be internally inconsistent, as in Figure 5 b and c there is no clear bandgap.
8. What causes the dip in the bulk spectrum of SiTe2 in Figure 5, and why there is no bulk bandgap visible in these spectra?
9. Can the authors add a clear structural model in the main text? The paper would also benefit for a better description of the figures as there are parts where arrows and other marks are not explained in the captions.

Reviewer #2

(Remarks to the Author)
Referee report
Experimental realization of the topologically nontrivial phase in monolayer Si2Te2
By Huang et al.

This is an interesting and timely study on the topological properties of two-dimensional van der Waals material (a monolayer Si2Te2 grown on a HfTe2 substrate). The authors made use of the computational 2D materials database to select a substrate for Si2Te2 that provides a high probability to keep the theoretically (DFT) predicted topological properties of monolayer Si2Te2 intact. HfTe2 turns out to be the ideal substrate for the growth of a topological nontrivial monolayer Si2Te2. The authors combine high quality scanning tunneling microscopy and spectroscopy data with state-of-the-art density functional theory calculations. The manuscript is solid, scientifically sound and well-written.

I have several concerns, comments/remarks:

1. The authors show in Figure 2b that the edge state of Si2Te2 exhibits a Dirac-like linear dispersion. A linear dispersion in a 1D system results in a constant density of states (see the supplementary materials section). In the experiments, however, the edge state shows up as a peak. The authors argue in the supplementary materials section that the dispersion relation becomes non-linear away from the Dirac point, resulting in van Hove singularity (resulting in a peak in the density of states). I do not see any evidence for this non-linearity in Figure 2(b) and therefore I suggest another explanation. The tunneling signal is usually dominated by states at the gamma point (k-parallel = 0). If, however, no states are available at the gamma point, the tunneling signal will be dominated by states that have a non-zero parallel momentum (and are closest to the gamma point). With increasing energy (w.r.t. to the Dirac point) the parallel momentum of the edge state increases (resulting in a decrease of the tunneling current). Does the decay of the dI/dV signal w.r.t. tot the Dirac point correspond to the Fermi velocity of the edge state (this Fermi velocity can be estimated from Figure 2(b))? Please comment on this point.
2. The scanning tunneling microscopy and spectroscopy shown in Figure 5(b) reveal that monolayer Si2Te2 hosts a well-defined edge state at an energy of a few meV below the Fermi level. The interior of Si2Te2 show a somewhat smaller state at an energy of about 10 meV below the Fermi level. Both states are also visible in spatially dependent dI/dV traces shown in panel (c) of Figure 5. In the supplementary materials section the authors elaborate on this 'interior' peak. I suggest shifting this discussion (first two paragraphs on page 14 of the supplementary materials section) to the main text.
3. In Figure 5(e)-(g) are dI/dV maps are shown for energies above the Fermi level. Can the authors also show a dI/dV map taken at the peak position of the edge state (a few meV below the Fermi level)?
4. The authors provide compelling evidence that monolayer Si2Te2 hosts an edge state, which seems to be quite robust and independent of the orientation of the edge. Strong evidence that this edge state is topologically protected by time reversal symmetry is, however, lacking. The authors should mention this explicitly in their manuscript. Please comment.

In summary, this is a solid and very interesting manuscript on a timely topic that will probably attract a lot of attention. In its present form, I cannot recommend publication. However, after satisfactory revision this manuscript might be suitable for publication in Nature Communications.

Reviewer #3

(Remarks to the Author)
The manuscript claims experimental realization of a quantum spin Hall (QSH) phase in monolayer Si_2Te_2 grown on $HfTe_2$ through combined DFT and STM/STS evidence. While the experimental dataset is visually compelling, the theoretical justification for the topological classification, substrate influence, and edge-state interpretation remains incomplete and relies heavily on indirect indicators. Several conceptual and methodological assumptions require stronger justification. The manuscript, in its present form, remains inconclusive regarding the topological nature of the grown samples. The following are my main comments.
• The manuscript contains several overly polished and promotional sentences, including parts of the abstract. While the use of automated tools for grammar and formatting is acceptable, some statements are phrased in a manner that appears assertive without sufficient supporting evidence. For example, statements describing continuous edge rings as direct spectroscopic confirmation of topological modes should be written in a more cautious and technically rigorous tone.
• The manuscript relies on Wannier charge center (WCC) evolution to claim $Z_2 = 1$ for both free-standing and substrate-supported systems. However, the calculation is only briefly described and lacks critical methodological details. The WCC results can strongly depend on several computational parameters, including k-mesh density, choice of Wannier orbitals, spread/localization quality, disentanglement window, and the accuracy of Wannier interpolation in reproducing the inverted energy manifold. The authors should clarify convergence with respect to these parameters and demonstrate that the Wannier representation faithfully captures the relevant band topology.
• In Supplementary Fig. S13, the nanoribbon band structure of Si_2Te_2 suggests that topological edge states coexist energetically with bulk states (for example, when considering a horizontal line at E = 0 eV in panels (c) and (d)). This raises concerns regarding the effectiveness of the material for dissipationless transport, since carriers injected into bulk states may strongly scatter with edge channels. Furthermore, phonon-mediated scattering in telluride-based systems is expected to be non-negligible and is not addressed in the manuscript.
• The decay length of approximately 2 nm is inferred indirectly from nanoribbon calculations where edge-state crossings appear only for ribbons exceeding this width. This argument remains indirect. A more convincing demonstration would involve plotting spatially resolved edge-state wavefunctions and explicitly showing their decay behavior from the boundary into the bulk region.
• I am not fully convinced by the interpretation of the dI/dV measurements as definitive evidence of topological edge states. The DFT results indicate that bulk states may also exist near the Fermi level. In addition, contributions from substrate-induced interface states cannot be excluded. The authors should provide more direct verification of the edge-state origin, potentially through momentum-resolved probes such as ARPES.
• The authors employ the HSE hybrid functional for electronic structure calculations. However, hybrid functionals are known

to modify band ordering in certain materials. In heavy-element telluride systems, GW-level quasiparticle corrections have been shown to alter band inversion strength and, in some cases, the topological classification. The authors should discuss the robustness of their conclusions with respect to the choice of exchange-correlation functional.
• Finally, the authors should comment on the structural and electronic stability of the heterostructure under ambient temperature and pressure conditions. The presented stability analysis appears limited to short-time simulations, and practical device applicability requires a clearer discussion of environmental robustness.

Version 1:

Reviewer comments:

Reviewer #1

(Remarks to the Author)
The authors answered all my questions seriously and added substantial material on the theory side, as well as resolved some of the experimental issues. Given that they also tone down a bit their claims, I recommend publication of their work.

Reviewer #2

(Remarks to the Author)
The authors have thoroughly revised their manuscript and addressed all the points that I have raised in my previous report in a satisfactory manner. They have also toned down their conclusion regarding the topological nature of the observed edge state. I recommend to accept this manuscript for publication.

Reviewer #3

(Remarks to the Author)
The authors have sufficiently addressed my concerns. Therefore, I recommend the acceptance of their manuscript without any further changes.

Open Access This Peer Review File is licensed under a Creative Commons Attribution 4.0 International License, which permits use, sharing, adaptation, distribution and reproduction in any medium or format, as long as you give appropriate credit to the original author(s) and the source, provide a link to the Creative Commons license, and indicate if changes were made.
In cases where reviewers are anonymous, credit should be given to 'Anonymous Referee' and the source.
The images or other third party material in this Peer Review File are included in the article’s Creative Commons license,

unless indicated otherwise in a credit line to the material. If material is not included in the article's Creative Commons license and your intended use is not permitted by statutory regulation or exceeds the permitted use, you will need to obtain permission directly from the copyright holder.
To view a copy of this license, visit https://creativecommons.org/licenses/by/4.0/

Reply-to-referees

Title: Experimental Realization of the Topologically Nontrivial Phase in Monolayer Si_2Te_2

Manuscript Number: NCOMMS-25-92789A

We thank all referees for their careful reading of our manuscript and for their valuable comments. Below, we provide a detailed **point-by-point response** to the questions and comments. **All changes made to the main manuscript and the Supplemental Materials have been highlighted.**

Response to Referee #1

This manuscript reports MBE growth of monolayer Si2Te2 on cleaved HfTe2 and the authors argue that the film is strain-free (giving a 1x1 structure), with a bandgap of about 300 meV and edge localized in gap states. Based on these (and theory) the authors declare Si2Te2 a QSH insulator. I think this work is promising and timely, and there is a good combination of STM/STS and DFT. However, the current paper, particularly the QSH claim, requires additional analysis, control experiments, and more internally consistent evidence.

Our response: We thank the referee for the positive assessment of our work and for the constructive comments and suggestions. We have carefully considered all concerns and revised the manuscript accordingly. In particular, we have moderated our statements regarding the quantum spin Hall interpretation and have added further DFT calculations, additional measurements, and expanded analyses to strengthen the evidence presented in the manuscript. Below we provide a detailed point-by-point response to each specific comment.

1. *The claim of topology rests primarily on the observation of in-gap states at the island boundaries. While I agree that this is consistent with a QSH edge-state picture, edge-enhanced LDOS alone is not unique to QSH systems. Trivial edge resonances, band bending, or reconstruction-induced states can mimic similar phenomenology. The authors should provide additional evidence or a control experiment (beyond edge vs bulk contrast) to justify the claim on the experimental realization of the QSH phase in Si2Te2.*

Our response: We thank the referee for this important comment. We agree that edge-enhanced local density of states (LDOS) alone is not, by itself, a unique fingerprint of a quantum spin Hall (QSH) phase. Trivial edge resonances, band bending, or reconstruction-induced states may, in principle, give rise to enhanced LDOS near boundaries. Therefore, additional evidence is required to support the interpretation of the observed edge-state features. Experimentally, the edge-state features in ML-Si_2Te_2 are reproducibly observed on multiple edge segments and different islands. The dI/dV maps reveal a boundary-localized spatial distribution extending approximately 1.5-2.0 nm into the island interior. While STM/STS measurements alone cannot completely exclude all

possible trivial edge-related states, the observed spatial extent and reproducibility are consistent with the behavior expected for the predicted topological edge modes.

To address the referee's request for additional evidence, we performed additional DFT calculations of the real-space charge-density distributions of the topological edge states in ML-Si_2Te_2 nanoribbons with different widths (Fig. R1). The calculations were performed for the Dirac-cone-related edge states within the energy window $E = E_F \pm 50$ meV (see Fig. 5f and Supplementarty Fig. 18 for details). As shown in Fig. R1, for ribbon widths of 4.59 nm and above, the edge-state charge density remains localized within approximately 2.0 nm of the ribbon boundaries, in good agreement with the experimentally observed spatial extent. This quantitative agreement between the STM/STS measurements and the DFT-calculated edge-state distribution provides additional support for the interpretation of the observed edge-state features.

Furthermore, a useful comparison can be made with our previous study of ML-Si_2Te_2 grown on Sb_2Te_3 substrate [*Adv. Funct. Mater. 32, 2208281 (2022)*]. In that system, the large interfacial strain drives ML-Si_2Te_2 into a trivial semiconducting phase, and no in-gap edge states were observed at the ML-Si_2Te_2/Sb_2Te_3 boundaries (see Fig. R2). In contrast, the present work realizes strain-free ML-Si_2Te_2 on $HfTe_2$ together with pronounced in-gap edge states. These comparative results provide an important control experiment supporting the interpretation of the observed edge states as being consistent with the predicted topologically nontrivial phase in ML-Si_2Te_2 grown on $HfTe_2$. We have added the new DFT results and the corresponding discussion to the revised manuscript and the Supplementary Materials.

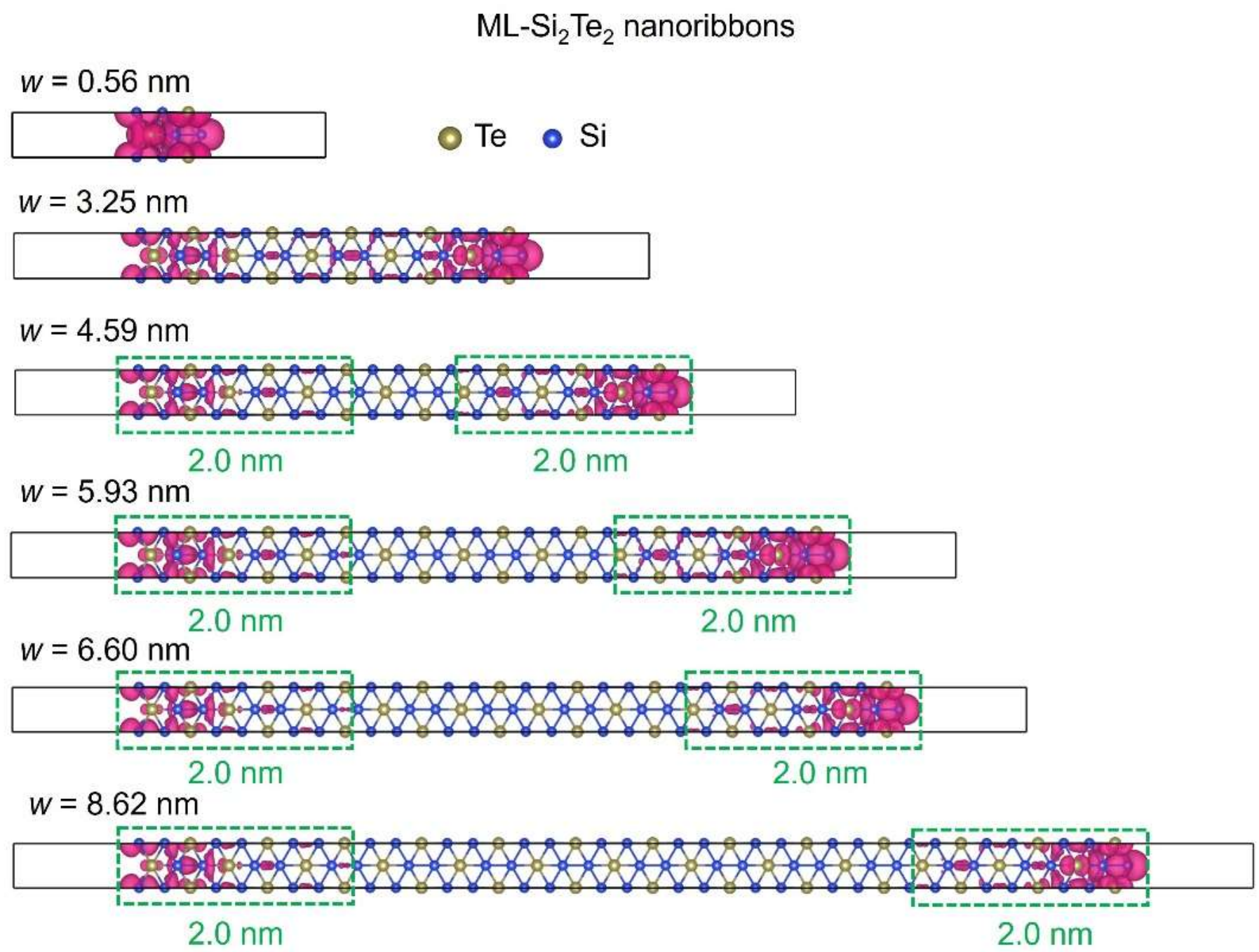

Fig. R1. Real-space charge-density distribution of the topological edge states in free-standing ML-Si_2Te_2 nanoribbons with widths of w = 0.56, 3.25, 4.59, 5.93, 6.60, and 8.62 nm. The charge

density was calculated within an energy window of ± 50meV around E_F. The isosurface value is set to 0.0001e/Å^3. Spin-orbit coupling is included in all calculations. Vacuum spacings of 20 Å were introduced along the lateral direction to suppress spurious interactions between periodically repeated nanoribbon edges. In addition, vacuum spacings of 30 Å were introduced along the out-o f-plane (z) direction to eliminate spurious interactions between periodically repeated ML-Si_2Te_2 la yers. The well-defined, spatially localized edge-state distributions with negligible inter-edge c oupling are highlighted by the green dashed boxes.

[Redacted]

Fig. R2. Reproduced from Fig. 4 of Ref. [*Adv. Funct. Mater. 32, 2208281 (2022)*]. **a** STM image of ML-Si_2Te_2 grown on 1QL-Sb_2Te_3 and corresponding side-view schematic. **b**,**c** dI/dV spectra measured on 1QL-Sb_2Te_3 film and on ML-Si_2Te_2 at the positions marked in (**a**). **d** Strain-dependent phase diagram and corresponding schematic band structures of ML-Si_2Te_2, calculated using the HSE06 hybrid functional. The red arrow indicates the strain regime corresponding to ML-Si_2Te_2 grown on Sb_2Te_3, where the system resides in a trivial semiconducting phase. **e** Upper panel: STM image showing a step between ML-Si_2Te_2 and the 1QL-Sb_2Te_3 film. Lower panel: 2D color map of spatially dependent dI/dV spectra taken along the green dashed line in the upper panel with 0.67 nm spacing. No in-gap edge states are observed at the ML-Si_2Te_2/Sb_2Te_3 boundary.

2. *The islands are very small, the reported edge-state spatial extent (\sim2-3 nm) takes a substantial fraction of the island lateral size, coupled with tip-convolution this would obstruct of measuring the true bandgap of the flakes. Additionally, edge states might hybridize with each other which*

could open a bandgap in the edge state spectrum. Ideally, statistics on larger islands (or a systematic scaling analysis) should be included.

Our response: We thank the referee for this constructive comment. We agree that the relatively small island size requires careful consideration of possible finite-size effects, including edge-state hybridization and tip-related convolution effects. To address the concern regarding the reliability of the band-gap determination, we revisited the statistical analysis of the band-gap measurements performed on 20 ML-Si_2Te_2 islands using 4 different STM tips. For clarity, the corresponding analysis, originally presented in Supplementary Fig. 10, is reproduced in Fig. R3. The band edges were extracted from the inflection points of the corresponding $\ln(dI/dV)$ spectra. The resulting average band gap is 284 ± 51 meV, which is reasonably consistent with the value of approximately 300 meV obtained in Fig. 4 and Supplementary Fig. 12 within the experimental uncertainty.

We further note that the determination of the bulk band edges was performed under stabilization conditions with relatively large bias voltages (±1 to ±1.5 V) and moderate tunneling currents (100–200 pA), corresponding to a comparatively large tip-sample distance. Under these conditions, the tunneling spectra are expected to be dominated by bulk electronic states, whereas the contribution from edge-localized states is expected to be significantly reduced. Therefore, even if residual edge-state contributions remain present owing to the finite lateral dimensions of the islands, the bulk electronic states are expected to dominate the tunneling spectra under the stabilization conditions employed here, enabling a reasonable estimation of the band-edge energies. Moreover, no clear spectroscopic signature that can be unambiguously attributed to finite-size-induced edge-state hybridization is observed in the measured spectra.

In addition, width-dependent DFT calculations of ML-Si_2Te_2 nanoribbons, presented in Fig. R1, show that edge-state hybridization is strongly suppressed once the ribbon width reaches approximately 4.59 nm. A large fraction of the ML-Si_2Te_2 islands included in the statistical analysis shown in Fig. R3 have lateral dimensions comparable to or larger than this value, indicating that finite-size-induced edge-state hybridization is expected to be substantially reduced for a large fraction of the islands included in the statistical analysis and is therefore unlikely to significantly affect the extracted band-gap values.

It is also important to emphasize that ML-Si_2Te_2 is a 2D material without a naturally occurring bulk counterpart, and the present work represents the first realization of strain-free ML-Si_2Te_2. Obtaining significantly larger islands remains experimentally challenging because the crystallization temperature of ML-Si_2Te_2 on $HfTe_2$ (~380°C) exceeds the decomposition temperature of $SiTe_x$ precursors (< 320°C). Although increasing the Te flux can partially compensate for this decomposition, further optimization of the growth process will be required to substantially enlarge the island size.

We have added the corresponding discussion to the revised manuscript with additional details provided in the Supplementary Materials.

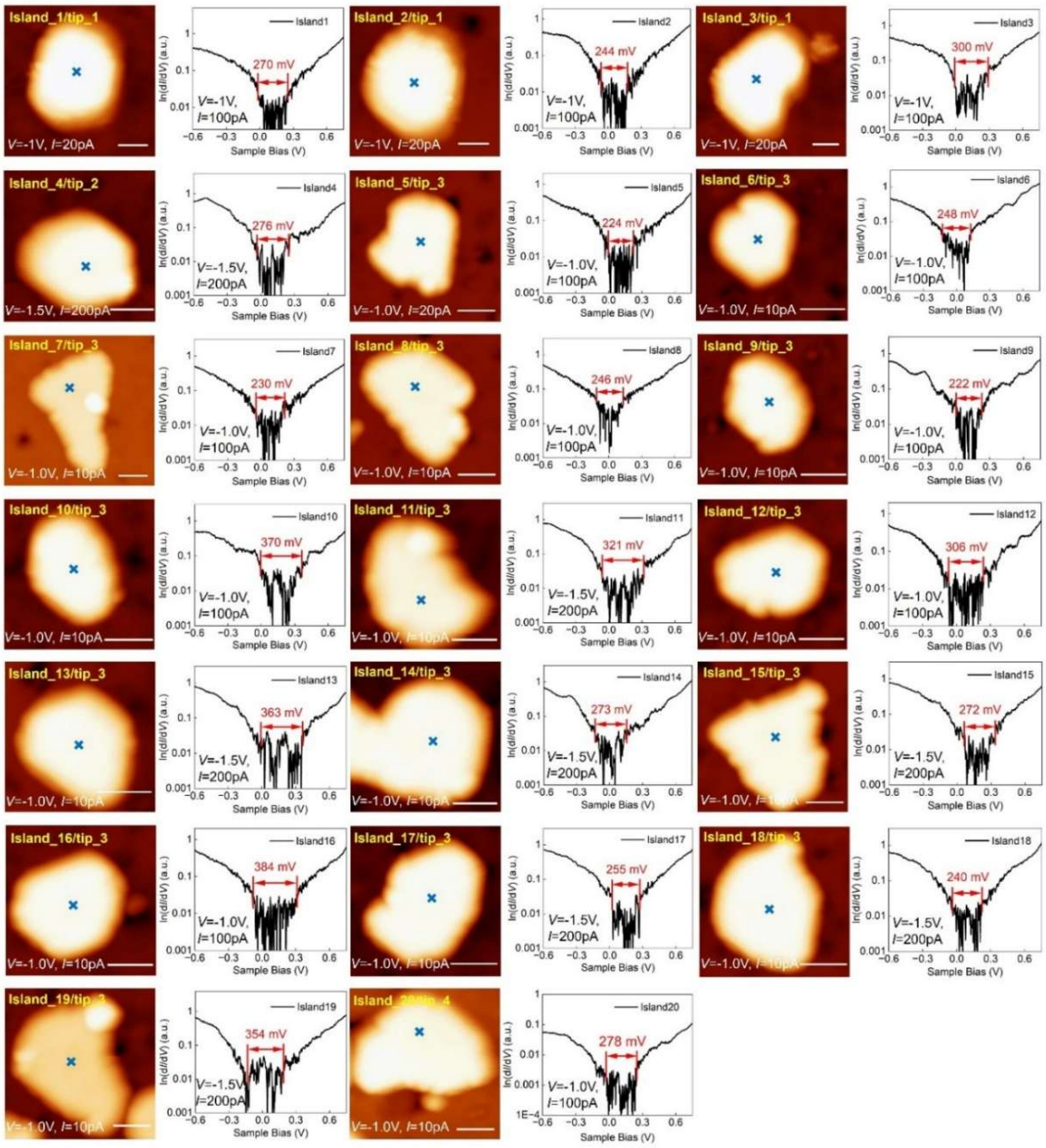

Fig. R3. dI/dV spectra acquired on 20 ML-Si_2Te_2 islands grown on $HfTe_2$ substrates using four different STM tips. Measurement positions are indicated by blue crosses in the corresponding STM images. Scale bars, 3.0 nm. All spectra are plotted on a logarithmic scale. A lock-in voltage modulation (V_{mod}) of 10 mV was used for all measurements. Data reproduced from Supplementary Fig. 10 of the original submission.

3. *Related to that there is another inconsistency. The penetration depth of the edges states is typically inversely proportional to the gap size. The authors have a rather large bandgap, which I would anticipate to give rise to very small spatial extend of the edge states, probably much smaller than the 2-3 nm measured here. Can the authors comment?*

Our response: We thank the referee for raising this insightful comment. We agree that large bulk gaps are often associated with more strongly localized topological edge states. However, the spatial

extent of realistic edge states may also depend on additional factors, including edge-state dispersion and substrate interactions. Additional insight can be obtained from the calculated real-space charge-density distributions of the edge states shown in Fig. R1. As shown, for ribbon widths of 4.59 nm and above, the edge-state wavefunctions are mainly confined within ~2 nm from the ribbon boundaries toward the interior. Furthermore, the inferred spatial extent remains essentially unchanged for ribbon widths of 4.59 nm and above, indicating that the edge-state localization is well converged with respect to ribbon width in this regime. These results are in good agreement with the experimentally observed spatial extent of 1.5-2.0 nm (Fig. 5e and Supplementary Fig. 17) and provide direct theoretical support for the inferred localization length of the edge states. Similar nanometer-scale spatial extent of topological edge states has also been reported experimentally in previous studies. For example, Fig. 3 of Ref. [*Phys. Rev. Lett. 109, 016801 (2012)*] shows that the edge states in Bi(111) bilayers extend over approximately 0.7-2.0 nm from the boundary, despite the presence of a sizable nontrivial bulk gap. We have added the new DFT results and the corresponding discussion to the revised manuscript and the Supplementary Materials.

4. *I do not agree with the statement by the authors that the edge state runs uninterrupted along the whole contour of the flake in Figure 5, there is an obvious and big interruption at the bottom of the flake. Can the authors provide additional evidence/more dIdV maps?*

Our response: We thank the referee for this helpful comment. We agree that our original wording describing the edge-state signal as a continuous ring around the entire island boundary was overly strong. We appreciate the referee for pointing this out. Accordingly, we have revised the related sentences throughout the manuscript. In the revised version, we instead describe the observed edge states as "pronounced edge states with boundary-localized features and a spatial extent of ~2 nm from the island boundary", which more accurately reflects the experimental observations.

To further address the referee's concern, we have also provided additional dI/dV maps acquired at different energies and with improved spatial resolution over the same area shown in Fig. 5d (see Fig. R4). These new data consistently reveal boundary-localized edge-state features around the island boundary and are in good agreement with the spatial distribution observed in Fig. 5e and Supplementary Fig. 17a,b. Although a local suppression of the dI/dV signal remains visible near the lower edge, the measurements consistently reveal a boundary-localized edge-state signal. We attribute this local suppression mainly to variations in local tunneling conditions and residual tip-related effects. More generally, real-space visualization of 1D edge states can be influenced by factors such as tip asymmetry, local edge morphology, and spatial variations in tunneling conditions [*C. Pauly, et al., Nature Physics 11, 338 (2015)*; *I. K. Drozdov et al., Nature Physics 10, 664 (2014)*]. As a result, local variations in dI/dV intensity can occur in restricted regions of STM/STS maps. We have added these additional experimental results and the corresponding discussion to the revised manuscript and Supplementary Materials.

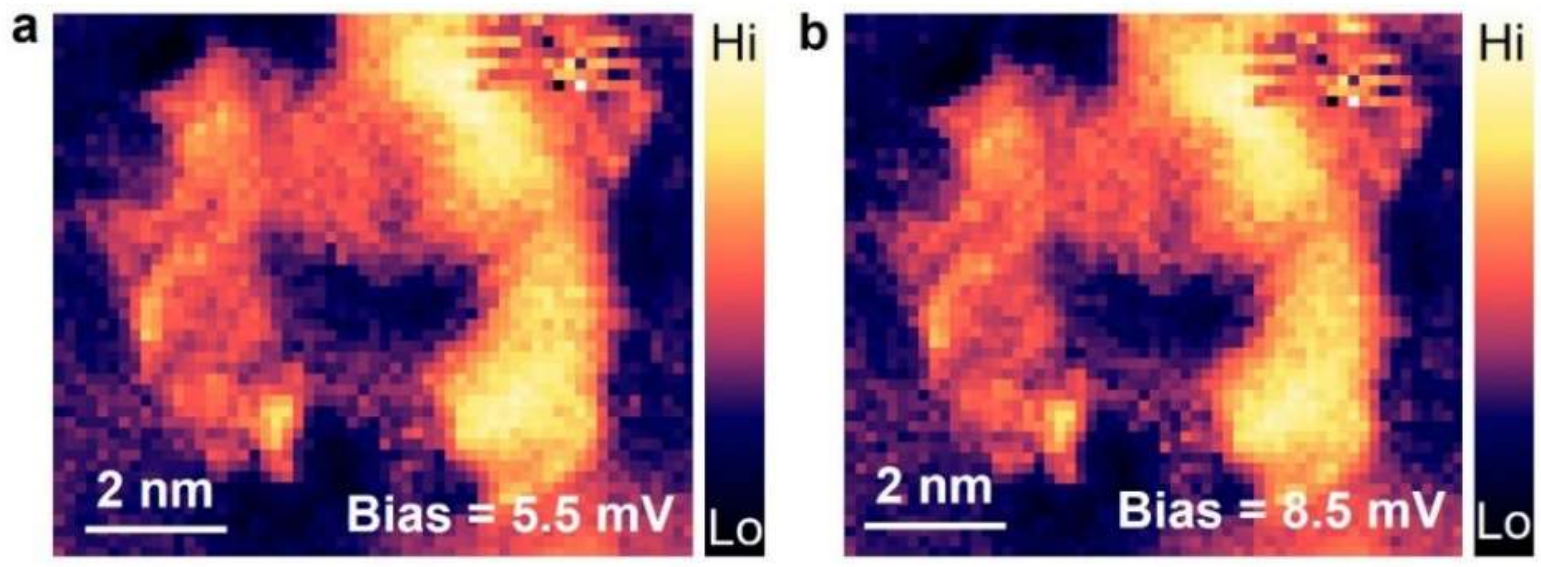

Fig. R4. d*I*/d*V* maps (V_{stab} = 0.2 V, I_{stab} = 100 pA, and V_{mod} = 2.0 mV) acquired at +5.5 mV (**a**) and +8.5 mV (**b**), corresponding to the same area shown in Fig. 5d. Both maps were acquired with twice the spatial resolution of the maps presented in Fig. 5e-g.

5. *Another concern that I have is that different terminations/reconstructions could alter the dispersion of the edge states. Given the islands appear to have rough edges, the STS signature should vary along different edges/segments or with this roughness. Can the authors discuss this further?*

Our response: We thank the referee for this constructive comment. To address the referee's concern, we now present d*I*/d*V* spectra acquired on different edge segments of the island shown in Fig. 5d of the main manuscript. As shown in Fig. R5, the edge-state peak near 7 mV is consistently observed at different edge locations, although its intensity and exact energy position show some spatial variation. Such variations are likely associated with differences in the local edge geometry, atomic-scale structure, and chemical environment. We have added these additional results and corresponding discussion to the revised manuscript and the Supplementary Materials.

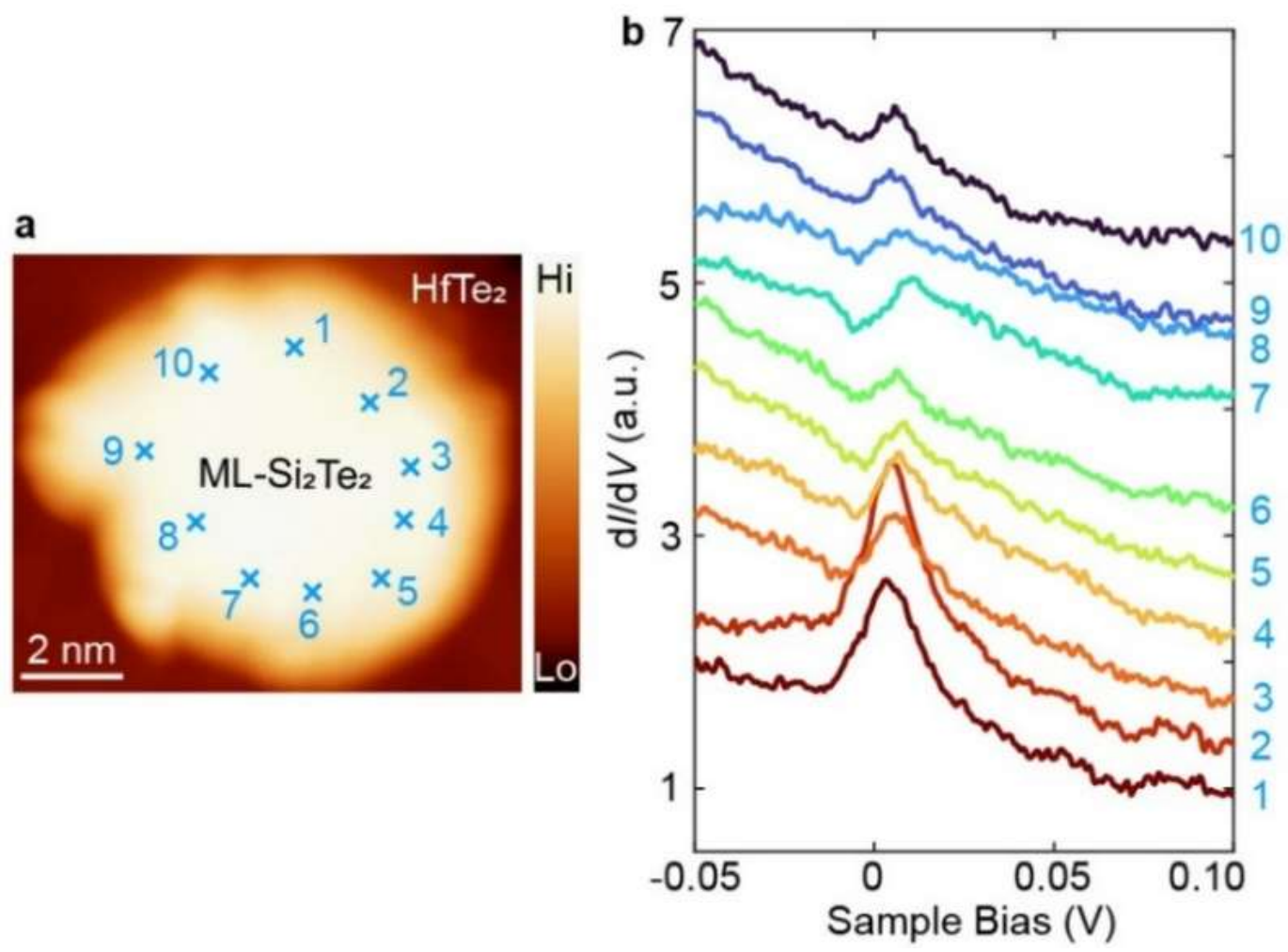

Fig. R5. Edge states measured at different edges of the ML-Si_2Te_2 island shown in Fig. 5d of the main manuscript. a, STM image (V = 1.0 V, I = 10 pA) of the ML-Si_2Te_2 island. **b**, d*I*/d*V* spectra (V_{stab} = -0.05 V, I_{stab} = 100 pA, and V_{mod} = 1 mV) acquired at different edges of the island, with the measurement positions indicated in (**a**).

6. *Typically, for topological systems, the edge-bulk correspondence tells that the boundary state is midgap, however the reported edge states are significantly offset from the midgap.*

Our response: We thank the referee for this insightful comment. We agree that, in QSH materials, the bulk-edge correspondence requires the topological edge states to connect the valence and conduction bands across the bulk gap. However, it does not require the most pronounced spectral feature observed in STM/STS measurements to appear exactly in the middle of the gap. In the present system, the ML-Si_2Te_2 islands are grown on a metallic $HfTe_2$ substrate, and the measured dI/dV spectra reflect the electronic structure of ML-Si_2Te_2 superimposed on a finite background contribution from the substrate. Near the Dirac point, the edge-state dispersion is approximately linear, resulting in a relatively weak and nearly energy-independent DOS contribution, which can be partially obscured by the finite background DOS from the metallic $HfTe_2$ substrate. Consequently, the experimentally observed edge-state signal is expected to be dominated by enhanced DOS features associated with the edge-state electronic structure. Furthermore, in realistic material systems, factors such as edge-dependent band dispersion and substrate interactions can shift the energy position of these enhanced DOS features away from the middle of the bulk gap. Therefore, the experimentally observed edge-state peak does not necessarily coincide with the mid-gap energy. We have clarified this point in the revised manuscript.

7. *The gap assignment from Fig. 4 is not fully convincing and may be internally inconsistent, as in Figure 5 b and c there is no clear bandgap.*

Our response: We thank the referee for raising this insightful comment. In this study, the ML-Si_2Te_2 islands are grown on a metallic $HfTe_2$ substrate. The substrate contributes a finite background DOS to the tunneling spectra, an effect that becomes increasingly pronounced at energies away from E_F. This behavior can be clearly seen in Fig. 4a, where the spectral intensity of $HfTe_2$ increases rapidly with energy. Consequently, a finite dI/dV background remains present throughout the investigated energy range in both Fig. 4 and Fig. 5b,c, including within the energy window associated with the bulk gap. Similar finite dI/dV backgrounds arising from conducting substrates have been reported in STM/STS studies of QSH systems grown on conducting substrates [*Nat. Commun 8, 659 (2017*; *Phys. Rev. Lett. 109, 016801 (2012)*]. Nevertheless, the presence of a finite background DOS does not preclude determination of the bulk band edges. In Fig. 4 and Supplementary Fig. 12, the band edges of ML-Si_2Te_2 grown on $HfTe_2$ can be identified from the broader spectral evolution, with the spectral contrast further enhanced by functionalized STM tips. In addition, the statistical analysis of the band-gap measurements performed on 20 ML-Si_2Te_2 islands using four different STM tips (Fig. R3) yields an average band gap of 284 ± 51 meV, which is in good agreement with the value of approximately 300 meV obtained from Fig.4 and Supplementary Fig. 12, as well as with the DFT-calculated band gap of 319 meV shown in Fig. 2a. Taken together, these results support the reliability of the band-gap assignment in ML-Si_2Te_2.

8. *What causes the dip in the bulk spectrum of SiTe2 in Figure 5, and why there is no bulk bandgap visible in these spectra?*

Our response: We thank the referee for this comment. In this study, the ML-Si_2Te_2 islands are grown on a metallic $HfTe_2$ substrate. As a result, the dI/dV spectra measured near the island center reflect the electronic structure of ML-Si_2Te_2 superimposed on a finite background contribution from the substrate. We therefore interpret the dip-like feature as arising from the finite background contribution of the metallic $HfTe_2$ substrate within the bulk-gap energy window of ML-Si_2Te_2. For the same reason, the tunneling conductance remains finite within the gap region and does not exhibit a fully vanishing hard-gap-like behavior. We further note that similar dip-like spectral feature near the island center was consistently observed on ML-Si_2Te_2 islands (Fig. 5, Fig. S14, and Fig. S15) grown on different $HfTe_2$ single-crystal substrates and measured using different STM heads and STM tips. These observations further confirm the reproducibility of this spectral feature in the ML-Si_2Te_2/$HfTe_2$ system. We have clarified these points in the revised manuscript and Supplementary Materials.

9. *Can the authors add a clear structural model in the main text? The paper would also benefit for a better description of the figures as there are parts where arrows and other marks are not explained in the captions.*

Our response: We thank the referee for this helpful suggestion. A structural model of the ML-Si_2Te_2/ML-$HfTe_2$ heterostructure is presented in Figs. 1b and 1c (top and side views, respectively) of the main text. In addition, we have added two 3D structural views of the ML-Si_2Te_2/ML-$HfTe_2$ heterostructure in Fig. 1e to provide a more intuitive visualization of the atomic stacking configuration (Fig. R6). We have also carefully revised the captions of the relevant figures to clarify the meaning of arrows, dashed lines, and other annotations.

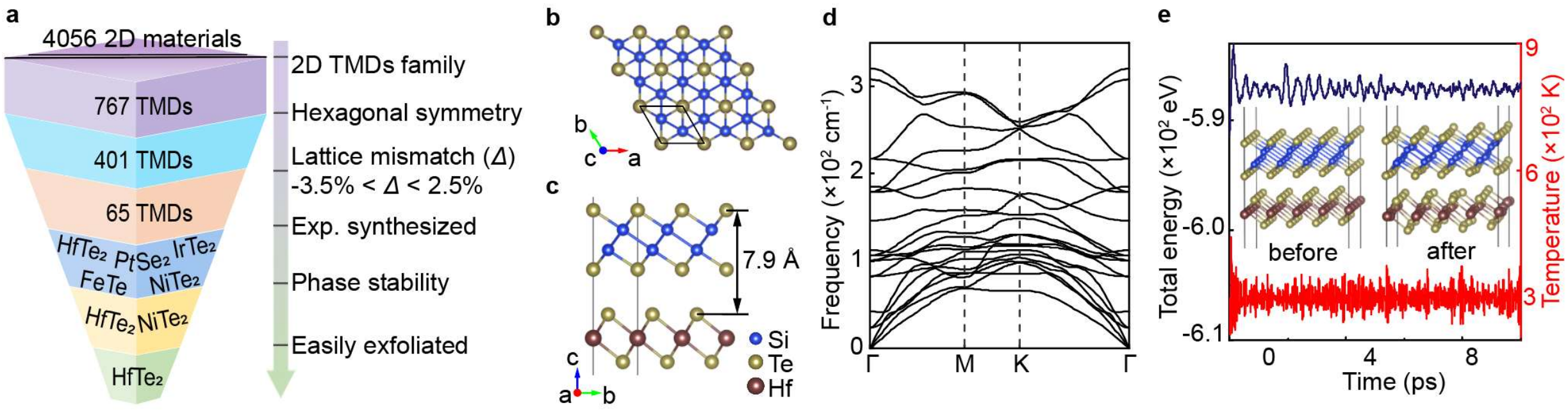

Fig. R6. Revised Fig. 1. Insets of Fig. 1e show 3D schematic views of the ML-Si_2Te_2/ML-$HfTe_2$ heterostructures before and after *ab initio* molecular dynamics simulations at 300 K.

Response to Referee #2

This is an interesting and timely study on the topological properties of two-dimensional van der Waals material (a monolayer Si2Te2 grown on a HfTe2 substrate). The authors made use of the computational 2D materials database to select a substrate for Si2Te2 that provides a high probability to keep the theoretically (DFT) predicted topological properties of monolayer Si2Te2 intact. HfTe2 turns out to be the ideal substrate for the growth of a topological nontrivial monolayer Si2Te2. The authors combine high quality scanning tunneling microscopy and spectroscopy data with state-of-the-art density functional theory calculations. The manuscript is solid, scientifically sound and well-written.

Our response: We thank the referee for the positive assessment of our work and for the thoughtful and constructive comments. We appreciate the referee's recognition of the significance of the material design strategy, the STM/STS experiments, and the DFT analysis. In response to the suggestions raised, we have carefully revised the manuscript and addressed each point in detail, including additional DFT results, new experimental data, and expanded discussions. Below, we provide detailed point-by-point responses to each of the referee's comments.

1. *The authors show in Figure 2b that the edge state of Si2Te2 exhibits a Dirac-like linear dispersion. A linear dispersion in a 1D system results in a constant density of states (see the supplementary materials section). In the experiments, however, the edge state shows up as a peak. The authors argue in the supplementary materials section that the dispersion relation becomes non-linear away from the Dirac point, resulting in van Hove singularity (resulting in a peak in the density of states). I do not see any evidence for this non-linearity in Figure 2(b) and therefore I suggest another explanation. The tunneling signal is usually dominated by states at the gamma point (k-parallel = 0). If, however, no states are available at the gamma point, the tunneling signal will be dominated by states that have a non-zero parallel momentum (and are closest to the gamma point). With increasing energy (w.r.t. to the Dirac point) the parallel momentum of the edge state increases (resulting in a decrease of the tunneling current). Does the decay of the dI/dV signal w.r.t. tot the Dirac point correspond to the Fermi velocity of the edge state (this Fermi velocity can be estimated from Figure 2(b))? Please comment on this point.*

Our response: We thank the referee for this constructive comment and for pointing out this important issue regarding the possible physical origin of the peak-like edge-state signal observed in the d*I*/d*V* spectra in Fig. 5. We agree that the non-linear band dispersion around the Dirac point is not clearly visible in Fig. 2b, which shows the calculated edge-stage dispersion for the ML-Si_2Te_2/ML-$HfTe_2$ heterostructure.

We also thank the referee for suggesting this alternative explanation based on the momentum dependent STM tunneling matrix element. We have added a corresponding discussion to the revised manuscript. In ML-Si_2Te_2, the Dirac point of the edge states is located at the Γ point (Fig. 2b), at the center of the Brillouin zone. Since STM tunneling generally favors states with small

parallel moment, states near the Γ point are expected to have a relatively high tunneling probability. As the energy moves away from the Dirac point, the edge-state momentum increases, which can reduce the tunneling matrix element and naturally lead to a peak-like enhancement of the dI/dV signal near the Dirac-point energy. Based on the linear part of the edge-state dispersion in Fig. 2b, we estimated the Fermi velocity to be $v_F = \frac{1}{\hbar}\frac{dE}{dk} = 5.47 \times 10^5 m/s$, which is a typical value for topological edge states in QSH systems (10^5 - 10^6 m/s). This value suggests that the parallel momentum of the edge state increases appreciably as the energy moves away from the Dirac point, supporting the physical plausibility of the momentum-dependent tunneling scenario proposed by the referee.

At the same time, we note that additional calculations for free-standing ML-Si_2Te_2 nanoribbons real noticeable nonlinear edge-state dispersions away from the Dirac point, around ± 50 meV (Fig.5f). This suggests that van-Hove-singularity-like effects may also provide an alternative possible explanation for the peak-like edge-state feature. Therefore, in the revised manuscript, we discuss both the momentum-dependent tunneling matrix-element effect and the possible van-Hove-singularity-like contribution as plausible explanations for the experimentally observed peak-like edge-state signal in the dI/dV spectra.

2. *The scanning tunneling microscopy and spectroscopy shown in Figure 5(b) reveal that monolayer Si2Te2 hosts a well-defined edge state at an energy of a few meV below the Fermi level. The interior of Si2Te2 show a somewhat smaller state at an energy of about 10 meV below the Fermi level. Both states are also visible in spatially dependent dI/dV traces shown in panel (c) of Figure 5. In the supplementary materials section the authors elaborate on this 'interior' peak. I suggest shifting this discussion (first two paragraphs on page 14 of the supplementary materials section) to the main text.*

Our response: We thank the referee for this helpful suggestion. In the revised manuscript, we have moved the related discussion on the interior peak from the Supplementary Materials to the main text for improved clarity and completeness.

3. *In Figure 5(e)-(g) are dI/dV maps are shown for energies above the Fermi level. Can the authors also show a dI/dV map taken at the peak position of the edge state (a few meV below the Fermi level)?*

Our response: We thank the referee for this helpful suggestion. We would like to clarify that the dI/dV spectra shown in Fig. 5b,c and the dI/dV maps shown in Fig. 5e-g were acquired on two different ML-Si_2Te_2/$HfTe_2$ samples. In Fig. 5b,c, the edge-state peak indeed appears slightly below E_F. However, for the island investigated in Fig. 5d-g, the corresponding dI/dV spectra reveal that the edge-state peak is located near +7 mV. To improve clarity, we now present the corresponding dI/dV spectra together with the dI/dV maps acquired on this island in Fig. R7. The corresponding

spectra show that the dI/dV maps acquired at +5 mV (Fig. R7c) and +7 mV (Fig. R7d) already probe the energy range near the edge-state peak for this island. To further strengthen this point, we now provide two additional dI/dV maps acquired at +5.5 mV and +8.5 mV with improved spatial resolution (Fig. R7f,g), both measured over the same area shown in Fig. R7a. The additional maps show consistent boundary-localized features, in agreement with the spatial features observed in Fig. R7c-e, and further support the reproducibility of the observed edge-state signal. Small variations in the exact edge-state peak position between different islands are expected owing to local differences in the electronic environment. We have added these new results and the corresponding discussion to the revised manuscript and Supplementary Materials.

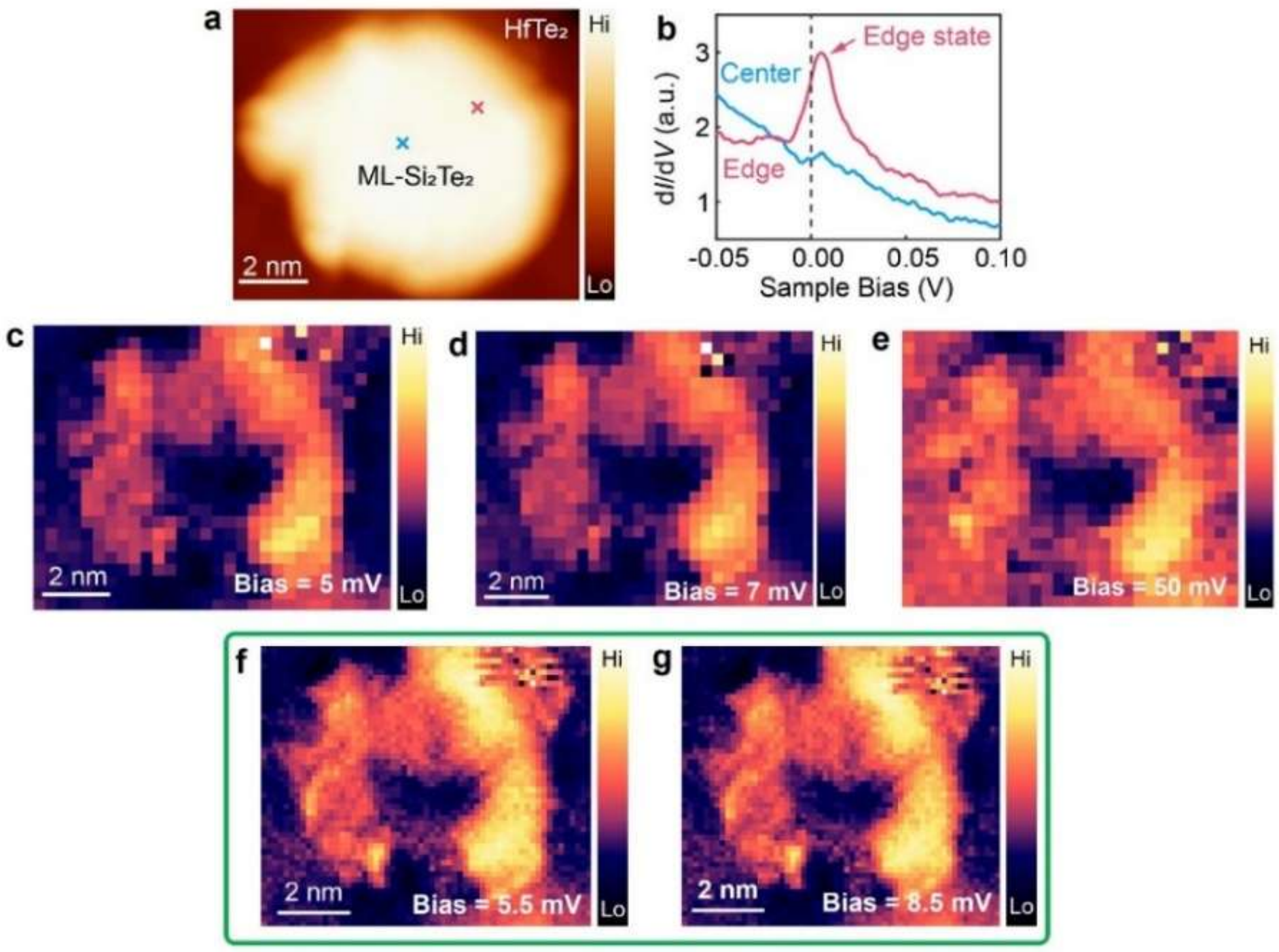

Fig. R7. Additional dI/dV maps of the edge-state features. a,**b**, STM image and dI/dV spectra reproduced from Fig. S15 of the Supplementary Materials. **c**-**e**, dI/dV maps reproduced from Fig.5e and Supplementary 17a,b of the revised manuscript. **f**,**g**, additional dI/dV maps (V_{stab} = 0.2 V, I_{stab} = 100 pA, and V_{mod} = 2.0 mV) acquired at +5.5 mV (**f**) and +8.5 mV (**g**) over the same area shown in Fig. 5d. Both maps were acquired with twice the pixel density of the maps shown in (**c**-**e**).

4. *The authors provide compelling evidence that monolayer Si2Te2 hosts an edge state, which seems to be quite robust and independent of the orientation of the edge. Strong evidence that this edge state is topologically protected by time reversal symmetry is, however, lacking. The authors should mention this explicitly in their manuscript. Please comment.*

Our response: We thank the referee for this important comment. We agree that, although the observed edge states exhibit robust boundary localization and appear largely independent of edge

orientation, the present STM/STS measurements do not directly establish time-reversal-symmetry (TRS) protection of these states. To better reflect the scope of the present evidence, we have thoroughly revised the manuscript to present the central claims in a more cautious and technically rigorous manner. In particular, we now clarify that the observed ~2 nm spatial extent and boundary-localized features of the edge states are consistent with characteristics of topological edge states, rather than direct proof of TRS-protected topological edge states. We further note that additional studies will be important to directly verify the TRS protection of these states. At the same time, the successful realization of strain-free ML-Si_2Te_2 paves the way for such future investigations. To explicitly reflect this point, we have added the following statement to the revised manuscript just before the Conclusions section: “although these states show a characteristic spatial extent and appear along edge segments with different local geometries, their protection by TRS has not been directly established in the present work. Further experiments, for example transport measurements, will be important for directly testing this protection”.

In summary, this is a solid and very interesting manuscript on a timely topic that will probably attract a lot of attention. In its present form, I cannot recommend publication. However, after satisfactory revision this manuscript might be suitable for publication in Nature Communications.

Our response: We thank the referee for the constructive comments and thoughtful suggestions, which have helped improve the clarity and rigor of the revised manuscript.

Response to Referee #3

The manuscript claims experimental realization of a quantum spin Hall (QSH) phase in monolayer Si_2Te_2 grown on $HfTe_2$ through combined DFT and STM/STS evidence. While the experimental dataset is visually compelling, the theoretical justification for the topological classification, substrate influence, and edge-state interpretation remains incomplete and relies heavily on indirect indicators. Several conceptual and methodological assumptions require stronger justification. The manuscript, in its present form, remains inconclusive regarding the topological nature of the grown samples. The following are my main comments.

Our response: We thank the referee for the careful reading of our manuscript and for the positive assessment of our experimental results. We appreciate the referee’s thoughtful and constructive comments. In response, we have carefully addressed the concerns raised by incorporating additional DFT calculations, expanding the relevant discussions, and clarifying several technical points. We have also revised the manuscript to present the central claims of this work in a more cautious and technically rigorous manner. Below, we provide detailed point-by-point responses to each of the referee’s comments.

1. *The manuscript contains several overly polished and promotional sentences, including parts of the abstract. While the use of automated tools for grammar and formatting is acceptable, some statements are phrased in a manner that appears assertive without sufficient supporting evidence. For example, statements describing continuous edge rings as direct spectroscopic confirmation of topological modes should be written in a more cautious and technically rigorous tone.*

Our response: We thank the referee for this important and helpful comment. We agree that several statements in the original manuscript could be interpreted as overly assertive relative to the evidence presented. To better reflect the scope of the present evidence, we have carefully revised the manuscript throughout to adopt a more cautious and technically rigorous tone. In particular, strong expressions such as "providing direct spectroscopic evidence of the predicted topological edge modes" have been revised to wording such as "consistent with the predicted topological edge modes". In addition, the last sentence of the Abstract has been revised to "Our results identify ML-Si_2Te_2 as a promising large-gap two-dimensional topological-insulator platform, offering opportunities for exploring topological phenomena and device concepts at elevated temperatures." We have also moderated related statements throughout the main text where appropriate. All the changes have been highlighted in the revised manuscript.

2. *The manuscript relies on Wannier charge center (WCC) evolution to claim $Z_2 = 1$ for both free-standing and substrate-supported systems. However, the calculation is only briefly described and lacks critical methodological details. The WCC results can strongly depend on several computational parameters, including k-mesh density, choice of Wannier orbitals, spread/localization quality, disentanglement window, and the accuracy of Wannier interpolation in reproducing the inverted energy manifold. The authors should clarify convergence with respect to these parameters and demonstrate that the Wannier representation faithfully captures the relevant band topology.*

Our response: We thank the referee for this important comment. We agree that the reliability of the Wannier charge center (WCC) evolution and the resulting topological invariant (Z_2) depends on the quality of the Wannier representation and the convergence of the relevant computational parameters. In this work, the WCC evolution was calculated using the WannierTools package based on the tight-binding Hamiltonian constructed from Wannier90. To ensure the reliability of the Wannier representation, we constructed maximally localized Wannier functions (MLWFs) from physically motivated atomic orbitals. For free-standing ML-Si_2Te_2, the Bloch states were projected onto the *s* and *p* (p_x, p_y, and p_z) orbitals of Si and Te atoms, yielding 32 MLWFs in total (including spin-orbit coupling). These orbitals accurately capture the valence and conduction bands associated with the topological band inversion. The resulting MLWFs showed good localization quality, with Wannier centers closely aligned with the atomic positions and stable spreads after convergence. Regarding the energy windows, the frozen window was chosen from -17.8 eV to 0.6 eV, ensuring that all occupied states together with the inverted energy manifold were faithfully reproduced by the Wannier interpolation. The outer disentanglement window was extended to 7.8 eV to provide a

sufficiently large Hilbert space for the projection. For the ML-Si_2Te_2/ML-$HfTe_2$ heterostructure, 58 MLWFs were constructed by including Hf-*d* orbitals (d_{z2}, d_{xz}, d_{yz}, d_{x2-y2}, and d_{xy}), together with the relevant *s* and *p* orbitals of Si and Te atoms to account for the hybridized interfacial bands. The frozen window (-15.60 eV to 2.25 eV) was carefully chosen to cover both the hybridized bands and the inverted states near E_F.

Furthermore, we confirmed that the calculated Z_2 index is well-converged with respect to the *k*-point sampling. While an 11×11×1 grid was used for the initial Wannierization, the WCC evolution was calculated using dense sampling of 101 *k*-point strings to ensure smooth Wannier-center evolution and numerical stability. The accuracy of the Wannier interpolation is demonstrated in Fig.R8, where the interpolated bands (red dashed lines) show excellent agreement with the directly calculated HSE+SOC band structures (black circles) within the energy window from -3.0 to 3.0 eV. This high-fidelity Wannier representation supports the reliability of the WCC evolution and the resulting topological analysis. We have added these methodological details and the corresponding discussion to the revised manuscript and Supplementary Materials.

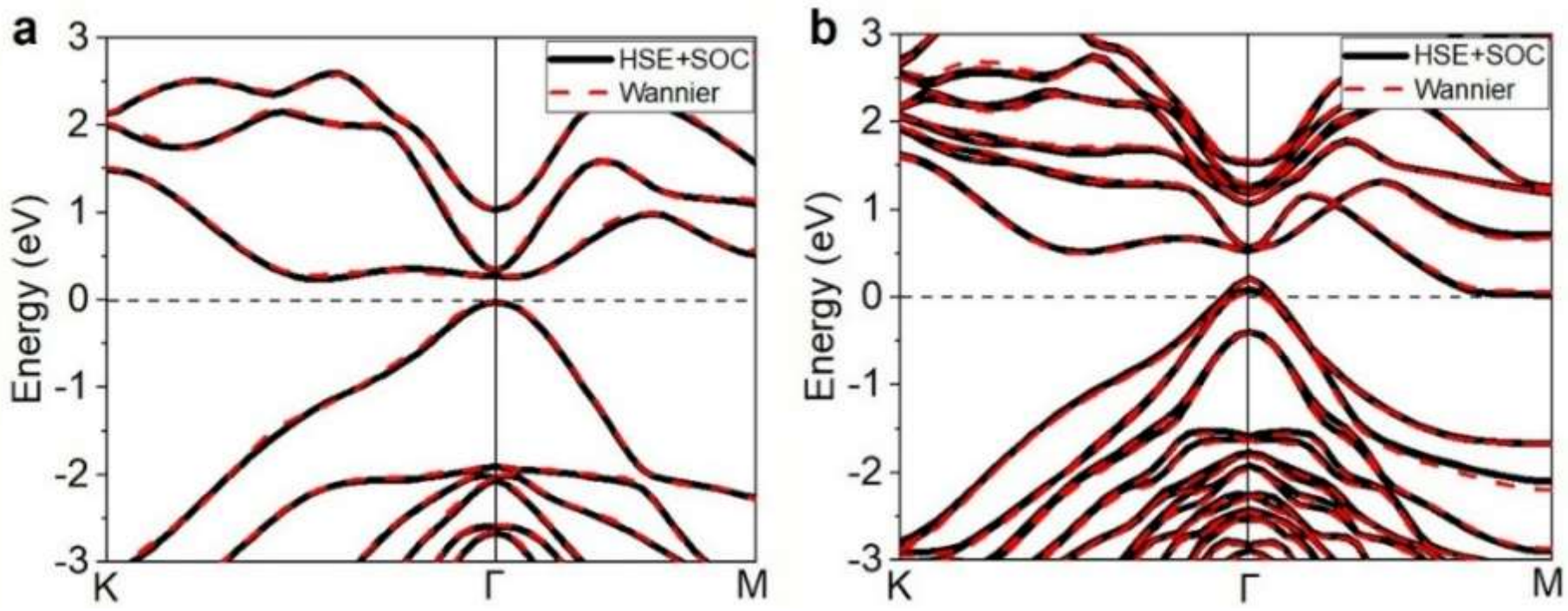

Fig. R8. Comparison of the calculated electronic band structures for (a) free-standing ML-Si_2Te_2 and (b) the ML-Si_2Te_2/ML-$HfTe_2$ heterostructure. SOC is included in all calculations. The black solid lines represent the direct HSE06 band structures, while the red dashed lines denote the Wannier-based tight-binding interpolated bands. The horizontal dashed lines indicate the E_F.

3. *In Supplementary Fig. S13, the nanoribbon band structure of Si_2Te_2 suggests that topological edge states coexist energetically with bulk states (for example, when considering a horizontal line at E = 0 eV in panels (c) and (d)). This raises concerns regarding the effectiveness of the material for dissipationless transport, since carriers injected into bulk states may strongly scatter with edge channels. Furthermore, phonon-mediated scattering in telluride-based systems is expected to be non-negligible and is not addressed in the manuscript.*

Our response: We thank the referee for this insightful and constructive comment. We first address the concern regarding the nanoribbon band structures presented in Supplementary Fig. S13 of the original submission. We agree that, if bulk-derived states overlap energetically with topological edge channels near the E_F, additional edge-to-bulk scattering channels may become possible and could affect edge-state transport.

Following the referee's comment, we therefore systematically re-examined the nanoribbon electronic structures by performing width-dependent calculations over a broader range of ribbon widths, from 0.56 nm to 8.62 nm. The updated results are shown in Fig.R9. These calculations show that the electronic structures of relatively narrow nanoribbons are strongly influenced by finite-size effects. In ribbons with insufficient width, residual inter-edge hybridization perturbs the edge-state dispersion, such that the energetic separation between edge-derived and bulk-derived states near E_F is not yet fully developed. As the ribbon width increases, the inter-edge coupling is progressively suppressed due to the increased separation between the two edges. For ribbon widths of 6.60 nm and larger, the edge states become well separated from each other and form a clear Dirac-like crossing at the Γ point. At the same time, the bulk-derived bands become energetically separated from E_F, resulting in a local energy window around E = 0 eV in which the electronic states are dominated by the topological edge channels. These results indicate that, in sufficiently wide nanoribbons, the topological edge states remain energetically separated from bulk-derived states near E_F, which is favorable for edge-state transport. In addition, as discussed in our response to Comment 4, we further calculated the real-space charge density of the edge states near the Dirac-point energy. These calculations show that, for sufficiently wide ribbons, the electronic states within this energy window are localized at the ribbon edges, with negligible weight in the interior region, indicating minimal overlap with bulk-derived states. We have replaced Supplementary Fig. S13 of the original submission with Fig. R9 and have incorporated the corresponding discussion into the revised manuscript and Supplementary Materials.

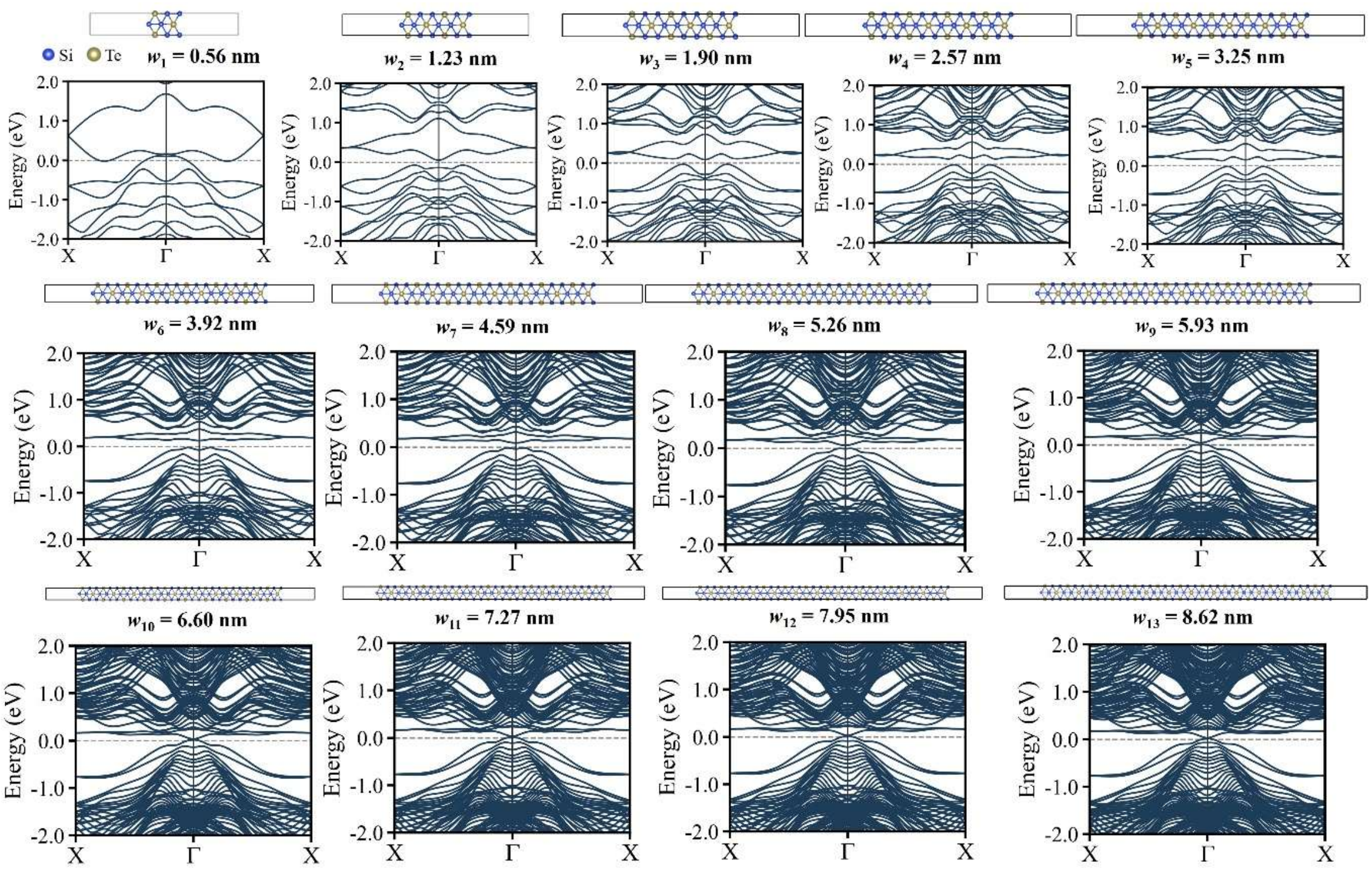

Fig. R9. DFT-calculated electronic band-structure evolution of free-standing ML-Si_2Te_2 nanoribbons with ribbon widths ranging from 0.56 to 8.62 nm. Schematic structural models of

each nanoribbon are shown together with the corresponding calculated electronic band structures. SOC is included in all calculations. Vacuum spacings of 20 Å were introduced along the lateral direction to suppress spurious interactions between periodically repeated nanoribbon edges. In addition, vacuum spacings of 30 Å were introduced along the out-of-plane (z) direction to eliminate spurious interactions between periodically repeated ML-Si_2Te_2 slabs. For d_n, n denotes the number of unit cells along the ribbon-width direction. The horizontal dashed lines indicate the E_F.

We next address the concern regarding phonon-mediated scattering in ML-Si_2Te_2. We agree with the referee that phonon-mediated scattering is an important factor for assessing finite-temperature transport in telluride-based systems. To provide a quantitative assessment of the electron-phonon coupling (EPC) strength, we performed density functional perturbation theory (DFPT) calculations using the Quantum ESPRESSO (QE) package for free-standing ML-Si_2Te_2 [*J. Phys.: Condens. Matter. 21, 395502 (2009)*; *J. Phys.: Condens. Matter. 29, 465901 (2017)*]. The calculated phonon spectra and EPC properties are shown in Fig. R10. The phonon dispersion exhibits no imaginary modes throughout the Brillouin zone, confirming the dynamical stability of ML-Si_2Te_2. The phonon density of states shows that the low-frequency modes are mainly associated with Te vibrations, whereas the higher-frequency modes are primarily related to in-plane Si vibrations.

The mode-resolved EPC constant λ_{qv}, Eliashberg spectral function $\alpha^2F(\omega)$, and the cumulative EPC $\lambda(\omega)$ [*J. Exp. Theor. Phys. 11, 696 (1960).*] are also presented in Fig. R10. The total EPC constant is $\lambda \approx 0.3$, indicating relatively weak EPC [*Nano Lett.* 16, 2522 (2016); *Chin. Phys. Lett. 43, 030711 (2026)*]. Importantly, the dominant EPC contributions originate from optical phonon modes in the range of 140-200 cm^{-1}, whereas the low-frequency acoustic modes, which are typically most relevant for charge-carrier scattering and transport, contribute comparatively weakly. These results indicate that the overall EPC strength in ML-Si_2Te_2 remains relatively moderate. Together with the sizable bulk gap and the energetic separation between edge-derived and bulk-derived states near E_F in sufficiently wide nanoribbons, this supports the expectation that phonon effects are unlikely to qualitatively destroy the topological edge-state character. We have added the DFPT calculations and the corresponding discussion to the revised manuscript and Supplementary Materials.

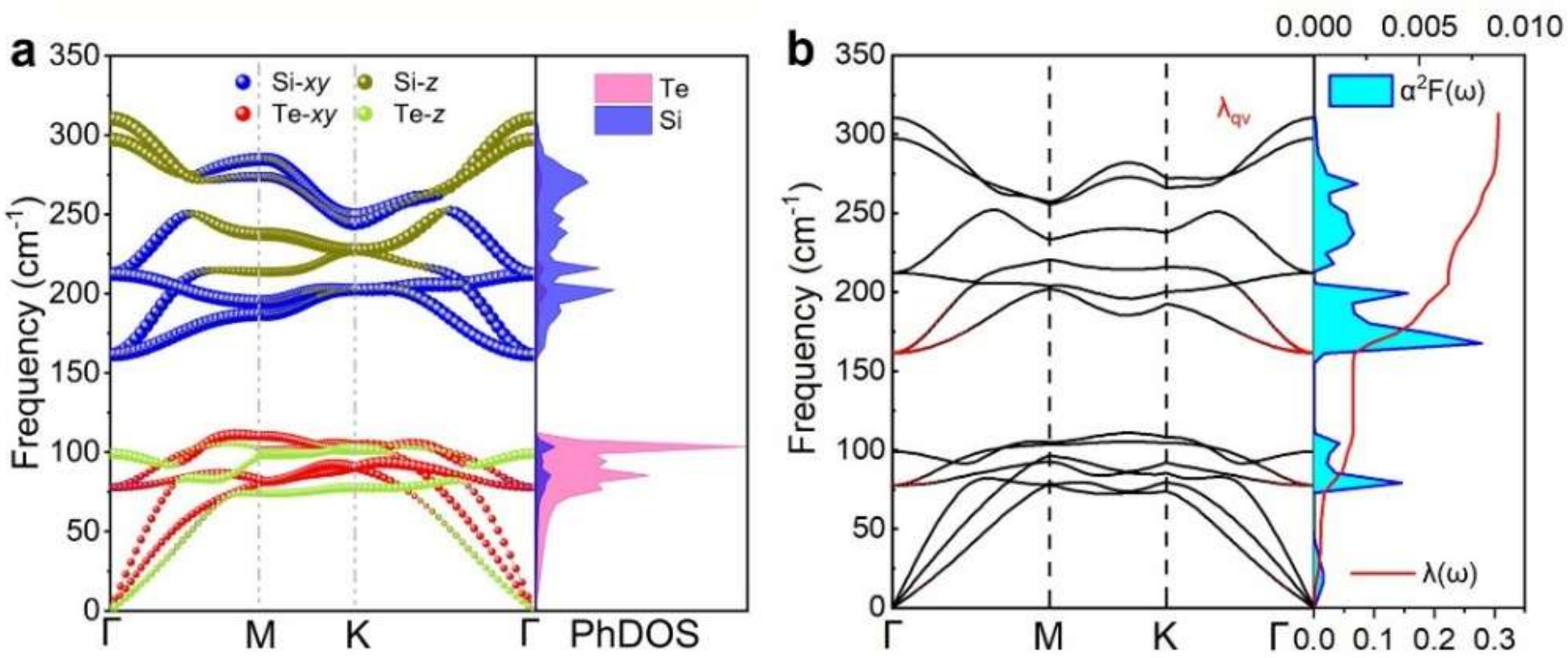

Fig. R10. Phonon spectra and EPC properties of free-standing ML-Si_2Te_2. a, Calculated phonon dispersion (left panel) and projected phonon density of states (PhDOS) (right panel) of

ML-Si_2Te_2. The phonon branches are weighted by the atomic-projected vibrational amplitudes of Si and Te atoms. Blue and red circles correspond to the in-plane vibrational modes of Si and Te atoms, respectively, while olive and light-green circles denote the corresponding out-of-plane vibrational modes. The right panel shows the atom-resolved PhDOS. **b**, Calculated EPC properties of ML-Si_2Te_2, including the mode-resolved EPC strength λ_{qv}, Eliashberg spectral function $\alpha^2F(\omega)$, and cumulative EPC constant $\lambda(\omega)$. The blue shaded area represents $\alpha^2F(\omega)$, while the red curve denotes $\lambda(\omega)$.

4. *The decay length of approximately 2 nm is inferred indirectly from nanoribbon calculations where edge-state crossings appear only for ribbons exceeding this width. This argument remains indirect. A more convincing demonstration would involve plotting spatially resolved edge-state wavefunctions and explicitly showing their decay behavior from the boundary into the bulk region.*

Our response: We thank the referee for this constructive suggestion. We agree that direct visualization of the edge-state wavefunctions provides a more convincing demonstration of their localization behavior. Therefore, we calculated the spatially resolved band-decomposed charge density associated with the topological edge states near E_F (within an energy window of ± 50 meV) for ML-Si_2Te_2 nanoribbons with different widths (w = 0.56, 3.25, 4.59, 5.93, 6.60 and 8.62 nm), as shown in Fig. R11. For narrow nanoribbons (w = 0.56 and 3.25 nm), the charge-density distributions exhibit substantial overlap between opposite edges, indicating strong inter-edge coupling arising from finite-size effects. As the ribbon width increases to 4.59 nm and above, the edge-state charge density becomes strongly localized at the ribbon boundaries and decays rapidly toward the ribbon interior, with negligible amplitude at the ribbon center. Importantly, the calculated spatial distributions show that the edge-state wavefunctions are mainly confined within ~2 nm from the ribbon boundaries toward the interior. Furthermore, the inferred spatial extent remains essentially unchanged for ribbon widths of 4.59 nm and above, indicating that the edge-state localization is well converged with respect to ribbon width in this regime. These results are in good agreement with the experimentally observed spatial extent of 1.5 - 2.0 nm and provide direct theoretical support for the inferred localization length of the edge states.

We have added the new DFT results and the corresponding discussion to the revised manuscript and the Supplementary Materials.

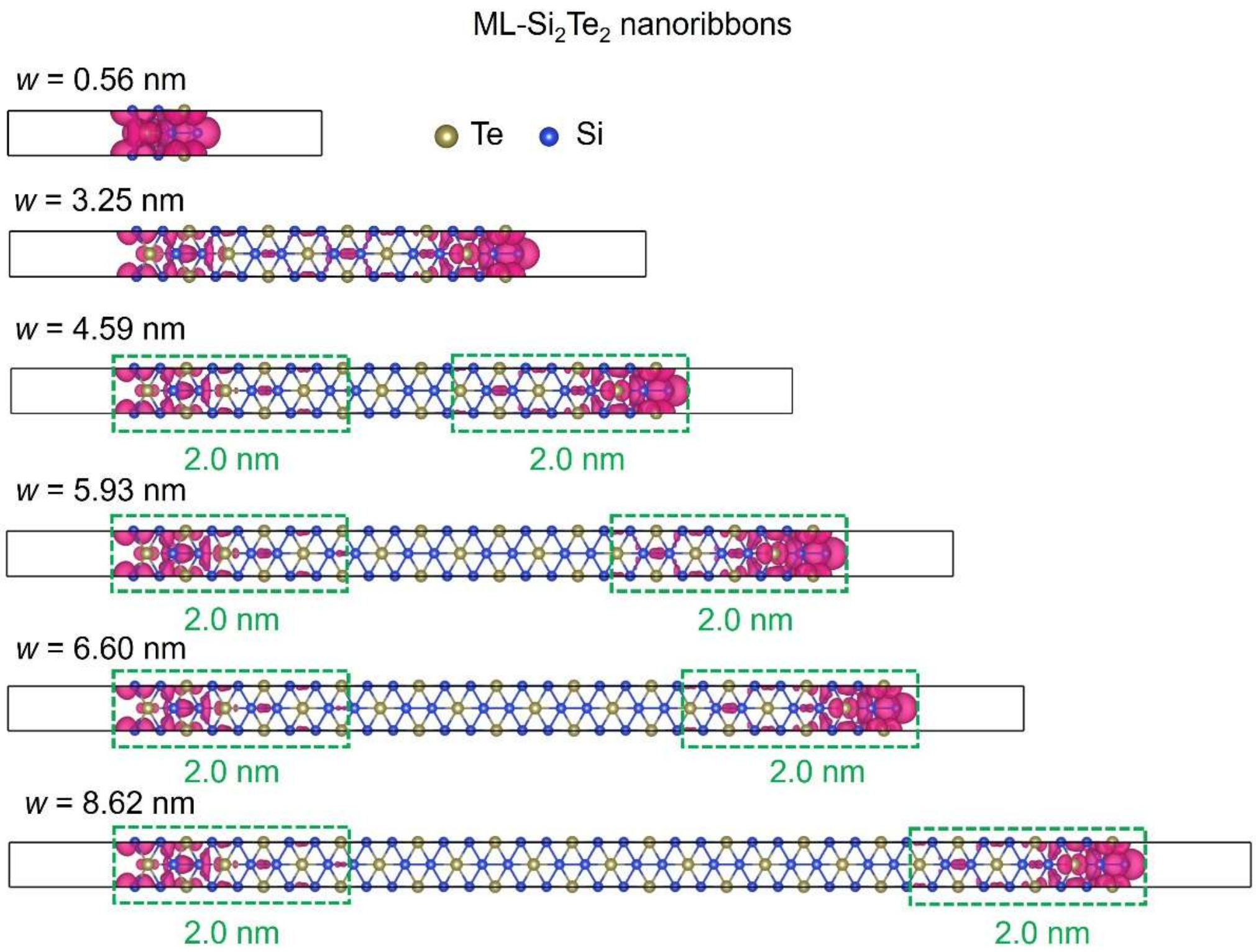

Fig. R11. Real-space charge-density distribution of the topological edge states in free-standing ML-Si_2Te_2 nanoribbons with widths of *w* = 0.56, 3.25, 4.59, 5.93, 6.60, and 8.62 nm. The charge density was calculated within an energy window of ± 50meV around E_F. The isosurface value is set to 0.0001e/Å³. Spin-orbit coupling is included in all calculations. Vacuum spacings of 20 Å were introduced along the lateral direction to suppress spurious interactions between periodically repeated nanoribbon edges. In addition, vacuum spacings of 30 Å were introduced along the out-of-plane (*z*) direction to eliminate spurious interactions between periodically repeated ML-Si_2Te_2 layers. The well-defined, spatially localized edge-state distributions with negligible inter-edge coupling are highlighted by the green dashed boxes. (Same as Fig. R1, shown again here for clarity.)

5. *I am not fully convinced by the interpretation of the dI/dV measurements as definitive evidence of topological edge states. The DFT results indicate that bulk states may also exist near the Fermi level. In addition, contributions from substrate-induced interface states cannot be excluded. The authors should provide more direct verification of the edge-state origin, potentially through momentum-resolved probes such as ARPES.*

Our response: We thank the referee for raising this insightful comment. We agree that d*I*/d*V* measurements alone should not be interpreted as definitive proof of topological edge states. Accordingly, we have revised the manuscript to present these results in a more cautious and

technically rigorous manner. In the present work, our interpretation is based on the combined consistency of STM/STS measurements and DFT calculations.

Experimentally, the edge-state features in ML-Si_2Te_2 are reproducibly observed on multiple edge segments and different islands. The d*I*/d*V* maps reveal a boundary-localized spatial distribution extending approximately 1.5-2.0 nm into the island interior. The DFT-calculated real-space charge-density distribution of the edge states in ML-Si_2Te_2 nanoribbons (Fig. R11) exhibits a comparable spatial extent of approximately 2.0 nm, in reasonable agreement with experiment. Taken together, these observations are consistent with the predicted topological edge-state picture in ML-Si_2Te_2.

Regarding the possible contribution of bulk states near E_F, we note that the recalculated nanoribbon band structures shown in Fig. R9 indicate that, in sufficiently wide nanoribbons, the topological edge states remain energetically separated from bulk-derived states near E_F. This result is also consistent with the calculated electronic structure of free-standing ML-Si_2Te_2, which does not contain intrinsic bulk states near the E_F (see Fig. S3b and S5b). For the ML-Si_2Te_2/ML-$HfTe_2$ heterostructure, the substrate-derived $HfTe_2$ bands contribute a finite background DOS within the gap-energy region of ML-Si_2Te_2 (Fig. 2). However, the d*I*/d*V* maps shown in Fig. 5e and Supplementary Fig. 17 demonstrate that the observed in-gap states are strongly localized near the island boundary, with a pronounced suppression of spectral intensity toward the island center. This spatial behavior is difficult to reconcile with a purely substrate-derived DOS contribution or a simple interfacial-state interpretation, as such states would be expected to extend over the island terrace rather than remain localized near the island boundary.

Regarding the suggestion of momentum-resolved probes such as ARPES, we note that conventional ARPES lacks the spatial resolution required to directly resolve isolated 1D edge states localized at individual ML-Si_2Te_2 island boundaries. In the present work, we therefore rely on the combined evidence from spatially resolved STM/STS measurements and DFT calculations, including the real-space charge-density distribution of the predicted edge states. While momentum-resolved or transport-based studies could provide valuable complementary information in future work, we believe that such investigations are beyond the scope of the present manuscript.

6. *The authors employ the HSE hybrid functional for electronic structure calculations. However, hybrid functionals are known to modify band ordering in certain materials. In heavy-element telluride systems, GW-level quasiparticle corrections have been shown to alter band inversion strength and, in some cases, the topological classification. The authors should discuss the robustness of their conclusions with respect to the choice of exchange-correlation functional.*

Our response: We thank the referee for this insightful comment. We agree that in heavy-element telluride systems, the band ordering and topological properties can be sensitive to the choice of exchange-correlation functional. To examine the robustness of our conclusions, we further performed GW calculations for free-standing ML-Si_2Te_2 and compared the band evolution obtained from both HSE and GW calculations with and without SOC (Fig. R12). In both approaches, the

same SOC-driven band inversion is found at the Γ point. Specifically, without SOC, the conduction- and valence-band-edge states are energetically close and show strong hybridization between Te-p and Si-p orbitals. Upon including SOC, the relative band ordering and orbital character near the band edges evolve in a qualitatively identical manner in both HSE and GW calculations, indicating that the nontrivial topological band inversion is robust against the choice of exchange-correlation functional within the present level of theory. Therefore, our conclusion regarding the nontrivial topological nature of ML-Si_2Te_2 remains unchanged. Moreover, our HSE results are consistent with a previous theoretical study of ML-Si_2Te_2 [*Phys. Rev. B 94, 201104(R), (2016)*]. We have added the GW calculations and the corresponding discussion to the revised manuscript and Supplementary Materials.

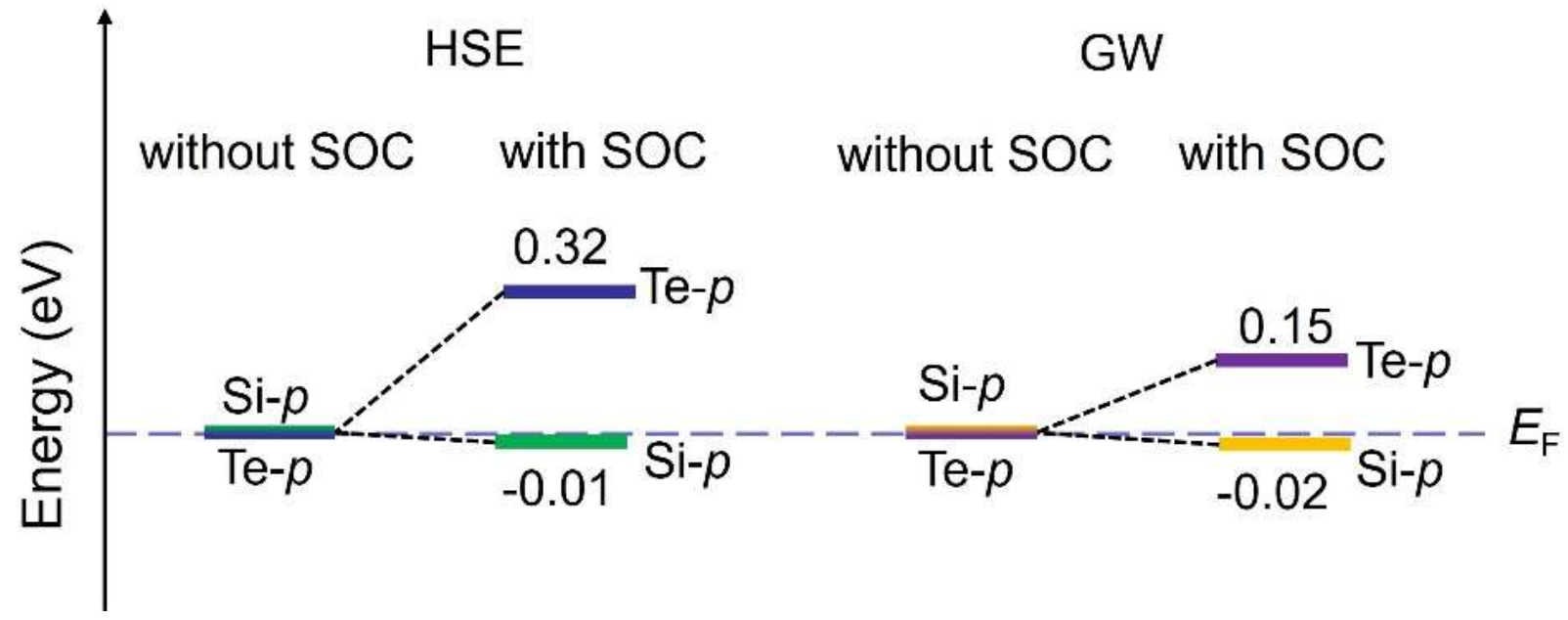

Fig. R12. Schematic illustration of the SOC-induced band evolution and band inversion at the Γ point in free-standing ML-Si_2Te_2, as obtained from HSE and GW calculations. The dashed line indicates the E_F. Colored bars denote the dominant orbital character of the band-edge states.

7. *Finally, the authors should comment on the structural and electronic stability of the heterostructure under ambient temperature and pressure conditions. The presented stability analysis appears limited to short-time simulations, and practical device applicability requires a clearer discussion of environmental robustness.*

Our response: We thank the referee for this important comment. We agree that the initial 10 ps AIMD simulations mainly probe short-time thermal stability and do not directly address long-term structural or electronic robustness under practical ambient conditions. To further examine this issue, we additionally performed 100 ps AIMD simulations (see Fig. R13), which yielded consistent results, with no apparent structural reconstruction or degradation observed throughout the simulation time. We have added these new AIMD results and the corresponding discussion to the revised manuscript and Supplementary Materials.

Experimentally, the structural robustness of ML-Si_2Te_2 grown on the $HfTe_2$ substrate is supported by the growth process itself. During epitaxial growth, the samples were annealed at ~385 °C, and in order to increase the coverage of ML-Si_2Te_2 islands, repeated growth/annealing cycles were performed on the same substrate. The persistence of ML-Si_2Te_2 islands under these repeated high-temperature processing steps suggests substantial structural stability of the heterostructure. In

addition, after growth, many samples were kept in the UHV chamber at room temperature for more than 24 hours before being transferred to the low-temperature STM system, while maintaining stable morphology and reproducible spectroscopic characteristics. This further supports room-temperature structural stability under vacuum conditions. From the electronic perspective, both the experimental results and DFT calculations identified that the bulk gap of strain-free ML-Si_2Te_2 remains larger than 220 meV, which is significantly greater than the thermal energy scale at room temperature (~ 25 meV), suggesting that thermal broadening is unlikely to close the bulk gap or fundamentally alter the nontrivial topological phase. We have added the corresponding discussion to the revised manuscript and Supplementary Materials.

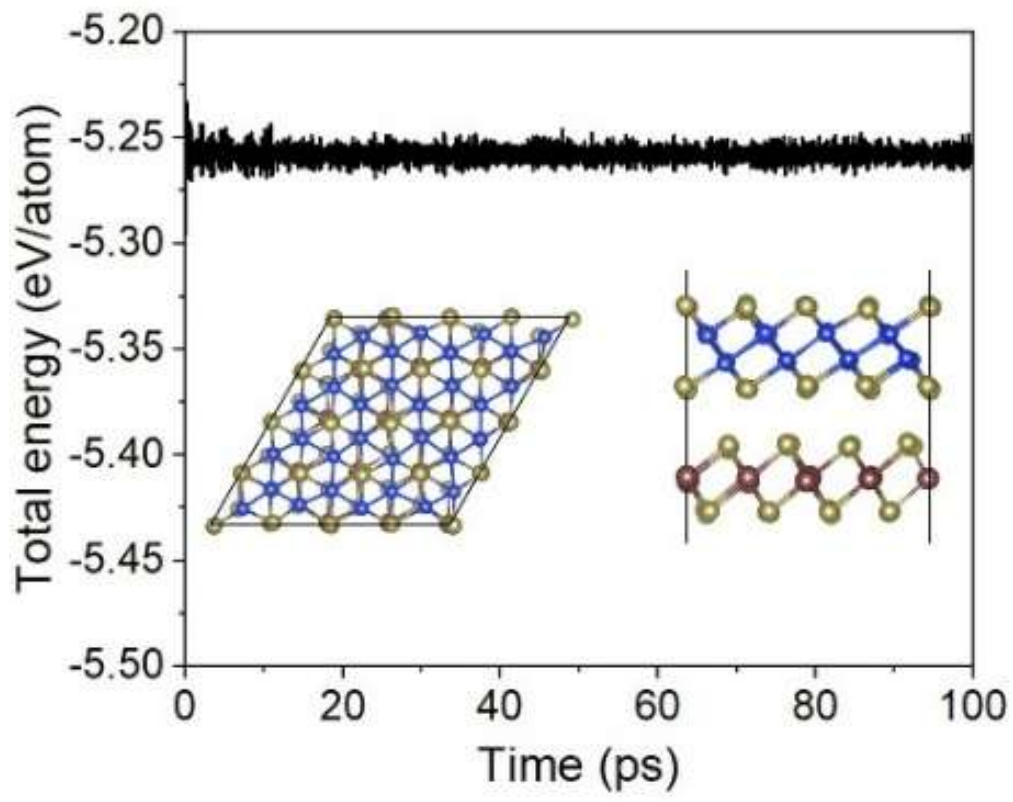

Fig. R13. Time evolution of the total energy during AIMD simulations of the ML-Si_2Te_2/ML-$HfTe_2$ heterostructure using a 4×4 supercell. The simulations were performed for 100 ps with a time step of 1 fs. Insets: Structural snapshots of the heterostructure after 100 ps of AIMD simulation.
